# Biologically informed deep learning to query gene programs in single-cell atlases

**DOI:** 10.1038/s41556-022-01072-x

**Published:** 2023-02-02

**Authors:** Mohammad Lotfollahi, Sergei Rybakov, Karin Hrovatin, Soroor Hediyeh-zadeh, Carlos Talavera-López, Alexander V. Misharin, Fabian J. Theis

**Affiliations:** 1 Institute of Computational Biology, Helmholtz Center Munich, Munich, Germany; 2 Wellcome Sanger Institute, Cambridge, UK; 3 Department of Mathematics, Technical University of Munich, Munich, Germany; 4 TUM School of Life Sciences Weihenstephan, Technical University of Munich, Munich, Germany; 5 Bioinformatics Division, WEHI, Melbourne, Victoria Australia; 6 Division of Infectious Diseases and Tropical Medicine, Ludwig-Maximilian-Universität Klinikum, Munich, Germany; 7 Division of Pulmonary and Critical Care Medicine, Feinberg School of Medicine, Northwestern University, Chicago, IL USA

**Keywords:** Computational biology and bioinformatics, Gene expression analysis

## Abstract

The increasing availability of large-scale single-cell atlases has enabled the detailed description of cell states. In parallel, advances in deep learning allow rapid analysis of newly generated query datasets by mapping them into reference atlases. However, existing data transformations learned to map query data are not easily explainable using biologically known concepts such as genes or pathways. Here we propose expiMap, a biologically informed deep-learning architecture that enables single-cell reference mapping. ExpiMap learns to map cells into biologically understandable components representing known ‘gene programs’. The activity of each cell for a gene program is learned while simultaneously refining them and learning de novo programs. We show that expiMap compares favourably to existing methods while bringing an additional layer of interpretability to integrative single-cell analysis. Furthermore, we demonstrate its applicability to analyse single-cell perturbation responses in different tissues and species and resolve responses of patients who have coronavirus disease 2019 to different treatments across cell types.

## Abstract (web summary)

Lotfollahi et al. present ExpiMap, a deep-learning model enabling interpretable reference mapping of RNA sequencing data using biologically defined entities, offering end-to-end analysis from dataset integration to functional interpretation.

## Main

The progress and development of experimental technologies^1–4^ and computational tools^5–9^ for single-cell genomics have enabled the construction of atlases with millions of cells serving as high-resolution coordinate systems^10^ for biological and therapeutic discoveries^11–14^. However, leveraging existing atlases poses a computational challenge known as reference mapping enabling rapid integration of newly generated datasets, denoted as a query. The transfer of knowledge from the reference to the query allows the rapid annotation of the query data^7^, imputation of missing modalities in the query^8,15^ and the discovery of novel populations^8,15^.

Single-cell reference mapping is growing in popularity^8,15–18^ to map query datasets by minimal modification of the reference atlas^19^. Existing reference mapping methods embed new query data into a reference latent space by removing technical differences, such as batch effects between the reference and the query, without access to reference data. However, the implicitly used latent dimensions for joint data representation are not directly interpretable.

An important trend in machine learning is the development of interpretable models, for example, by adding statistical assumptions to learned latent spaces or including prior information from validated mechanisms or other data^20^. As the former disentanglement approaches have not yielded sufficiently useful latent spaces in our context^21–23^, we hypothesize that using prior information may help identifiability. In particular, we aim to leverage known or newly learned gene programs (GPs) to contextualize query data by answering various questions, including ‘which GPs are disturbed in a disease query data compared with the healthy reference?’ and ‘which biological programs explain a novel population in the query?’ By thus making reference mapping interpretable, it can move beyond mere data alignment between query and reference and be used for further interpretation of query data for example, in the case of disease perturbation versus a healthy atlas. Currently, the standard approach for identifying biological programs in query cells compared with a reference atlas is to test for differentially expressed genes and downstream gene set enrichment. However, the differential expression on an atlas consisting of cells from an arbitrary number of studies with variable degrees of biological and technical heterogeneity represents a challenge for statistical analysis. The currently accepted best practices^24,25^ suggest that differential expression should be performed on non-integrated expression data and not on the corrected expression values after integration; hence, statistical models should account for complex experimental designs and adjust for batch effects, which is further hampered by modelling constraints such as parameter identifiability. Instead of using simpler non-parametric statistical tests, both biologically relevant and irrelevant genes may be captured, which may compromise the accuracy of enriched gene set terms.

Collectively, it may be useful to have interpretable embeddings directly associated with validatable GPs in the context of atlas-wide comparisons to capture the relevant biological signals while accounting for nonlinear batch effects. This end-to-end approach is common in deep learning and has been shown to outperform classical approaches that use sequential regularization and analysis^20^. Interpretable reference mapping requires incorporating domain knowledge^20^, such as curated GPs, into the representation learning model to guide interpretation and exploration. Including domain knowledge to design ‘domain-informed’ deep learning architectures has been shown to improve the performance on challenging prediction tasks, from tumour type^26^ to protein structure^27^. Earlier works proposed incorporating regularized linear decodes to include domain knowledge into autoencoders for single-cell data^28^, with scalable and expressive embeddings compared with existing factor models, such as f-scLVM^29^. Recent approaches such as VEGA^30^, scETM^31^ and pmVAE^32^ also feature variational autoencoders with linear decoders or training separate VAEs for each GP yet connected via a global loss in the case of pmVAE. Yet, accounting for the incompleteness of domain knowledge and learning new knowledge de novo from the data, rather than being locked into prior-based feature design, are not fully addressed by existing methods. Finally, going beyond single dataset analysis towards large-scale data integration and reference mapping while injecting domain knowledge remains challenging.

In this Technical Report, to address these challenges, we propose to build a machine learning system that exploits the knowledge of the underlying biological phenomenon for single-cell representation learning (as outlined more generally in the idea of ‘differential programs’^20^ recently). We construct an ‘explainable programmable mapper’ (expiMap) as an interpretable conditional variational autoencoder^7,33,34^ that allows the incorporation of domain knowledge by performing ‘architecture programming’, that is, constraining the network architecture to ensure that each latent dimension captures the variability of known GPs. We apply an attention-like mechanism^35^ to select the relevant GPs for each reference dataset. This helps with the prioritization of essential gene sets but also allows the inclusion of genes not initially included in annotated GPs, thereby addressing the incomplete nature of the knowledge database. To identify new variations unique to the query data, such as disease effects, we identify de novo GPs in addition to the known GPs in knowledge bases by learning disentangled latent representations. The framework can be used to automatically identify and explore biological processes in normal and disease states when mapping new query datasets to the atlas while maintaining comparable integration performance to existing data integration methods.

## Results

### Interpretable single-cell reference mapping using expiMap

Linear methods, such as principal component analysis (PCA)^36,37^ or matrix factorization^38,39^, learn a representation of the data where each dimension of the latent space can be explained using a weighted combination of the input, such as gene expression. This interpretability comes at the cost of the model’s limited capacity (for example, only capturing linear relationships) to fit the data. In contrast, nonlinear methods using deep neural networks^40,41^ come with a larger capacity at the expense of reduced model interpretability.

**Fig. 1:**
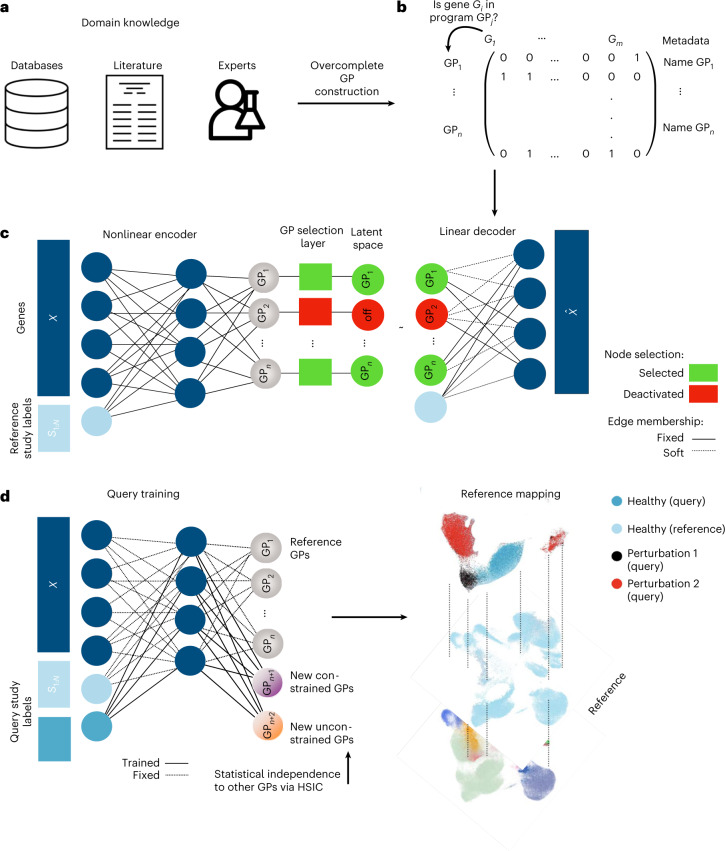
Biologically informed reference mapping using expiMap. **a**,**b**, Domain knowledge from databases, articles and expert knowledge (**a**) is used to construct a binary matrix of GPs (**b**). **c**, The model is trained on reference data, received gene expression and study labels for each cell to encode a set of latent variables representing GPs. The GPs are pruned and enriched by the model using a group lasso and gene-level sparsity regularization, respectively, and fed into a linear decoder. The GP matrix is then used to program the neural network architecture by wiring the model parameters of the decoder to learn a specific GP for each latent dimension. **d**, The reference model is expanded and fine-tuned upon mapping query data using architecture surgery, whereas new learnable latent GPs are added and trained with the query data. The decoder architecture equals **c** with the difference that only highlighted weights of newly added GPs are trainable in the encoder and decoder. To make sure these newly learned unconstrained GPs do not overlap with reference GPs, we employ statistical independence constraints.

Here we aim to design a system that can provide biologically interpretable answers to queries of an integrated representation of multiple (denoted by *N*) reference single-cell datasets and custom GPs. These can be gene lists from existing curated databases^42,43^, lists extracted from literature^44^ or individually curated gene sets (Fig. 1a). This knowledge is transformed into a binary GP matrix, in which each row is a GP. Each column denotes the membership of a gene in that program (Methods and Fig. 1b).

We wire the network weights using the GP matrix such that each latent variable contributes to the reconstruction of a set of genes defined by the GP similar to^28,30^. The model receives a gene expression matrix from *N* different single-cell studies (*X*) and an additional vector for corresponding one-hot encoded study labels (*S*_1:*N*_) for each cell, for example, the experimental laboratories or sequencing technologies (Fig. 1c). The adopted variational autoencoder architecture^7,40^ leverages a nonlinear encoder for flexibility and a linear decoder^45^ for interpretability. The latent space dimension chosen is equal to the number of GPs. The weights from each latent dimension (that is, latent GP) to output are programmed according to the GP matrix so that a latent GP can only contribute to the reconstruction of genes in a particular GP (denoted as ‘fixed membership’ in Fig. 1c). As annotated GPs are often incomplete, we allow the inclusion of other genes in each GP by applying L1 sparsity regularization to genes not initially labelled to belong to that GP (denoted as ‘soft membership’ in Fig. 1c). This enables the model to leverage the sparse selection of other genes, which helps in the reconstruction and therefore accounts for incomplete domain knowledge, to refine ontologies and pave the way towards a data-driven alternative means to learn GPs (see later results).

However, the number of GPs may be very large, and potentially redundant, and not all are relevant for every atlas. To select only informative GPs, an attention-like mechanism is implemented with a group lasso regularization layer in latent space (Methods), which de-activates GPs that are redundant or do not contribute to the reconstruction error of the model. The model is trained end to end and can thus be used to construct reference atlases with interpretable embedding dimensions, which we can leverage to analyse integrated datasets.

On the basis of this pre-trained, interpretable reference model, we propose employing transfer learning, as outlined in architectural surgery^15^ (Methods), to map new datasets into the reference. We modify the strategy of fine-tuning conditional weights in scArches allowing the model to learn new GPs that are not included in the reference model. This is achieved by adding new latent space dimensions, that is, nodes with trainable weights in the bottleneck layer of the model (Fig. 1d and Methods), while keeping the rest frozen. We implement two ways of learning these new GPs: either by learning GPs confined to pre-defined genes (denoted as ‘new constrained’ in Fig. 1d) that were not present or those that have been de-activated in the reference model. In addition, the model may also learn de novo GPs as realized by an L1-regularized gene before capture of new variations in the query data without pre-defined gene sets (denoted as ‘new unconstrained’ in Fig. 1d). The limited learning capacity of the model at the reference mapping stage, due to frozen weighting, enforces an information bottleneck (that is, a reduced capacity to learn and store information), encouraging the new nodes to learn important and potentially disentangled^46^ sources of variations in the query data. We further employ the Hilbert–Schmidt independence criterion (HSIC)^22,47^, a kernel-based measure of latent variable independence^47^, to further enforce independence between old and new unconstrained GPs learned during query optimization (Fig. 1d).

The probabilistic representation learned by expiMap as a Bayesian model allows the performance of hypothesis testing on the integrated latent space of the query and the reference accounting for technical factors (Methods). The hypothesis testing is performed at the GP level, identifying differential GPs between two groups of cells by sampling from the group’s posterior distribution of the latent variables. The ratio between two hypothesis probabilities is reported by the Bayes factor. Later, we demonstrate how this ability helps to identify GPs associated with a perturbation in the query data compared with the healthy reference. When talking about the results of the expiMap Bayes test, we call the GPs ‘enriched’ if their absolute logarithmic Bayes score is greater than or equal to 2.3.

Collectively, through expiMap, we propose an approach to learning interpretable, domain-aware representations of single-cell datasets for the integrative analysis of reference and query data. Further, we propose a modified version of architecture surgery that goes beyond pre-defined domain knowledge while retaining interpretability. This allows contextualizing the query data with the reference data within a specific GP to answer the user’s biological questions.

### expiMap parses transcriptional response to IFN-β

**Fig. 2:**
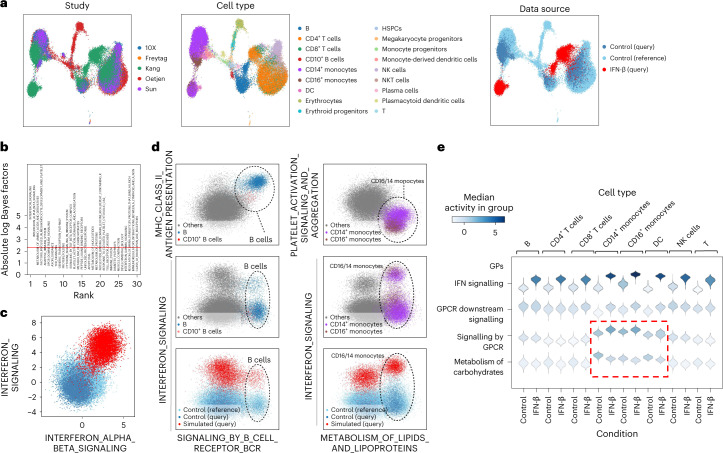
ExpiMap resolves GPs after IFN-β perturbation. **a**, UMAP representation of the query control and IFN-β-stimulated cells from eight patients (*n* = 13,576 cells) mapped onto a healthy immune reference from four different studies (*n* = 32,484 cells) using expiMap. Colours demonstrate study (left), harmonized cell type (middle) and data source (right). HSPCs, haematopoietic stem and progenitor cells. **b**, Differential GP analysis results between query IFN-β and control cells from the query and reference. The *x* axis shows the ranking of GPs; the *y* axis denotes the significance (absolute log-Bayes factor) of each GP. **c**, Visualization of both the reference and query data in the context of the top two most significant expiMap latent GPs in **b**. Each dot shows the latent GP score of each cell. **d**, Visualization of the query and reference in various GPs, delineating cell types or perturbation states for B cells and CD14^+^/16^+^ monocytes. **e**, The activity of the most differentially active GP terms in CD14^+^ monocytes after IFN-β stimulation. Each violin plot demonstrates the distribution of latent GP values across different cell types. The dashed square highlights GPs characterizing the myeloid-specific response to IFN-β. Source data

One of the ultimate goals in building large, single-cell atlases is studying the effect of perturbations (for example, disease) and contextualizing it within a given healthy reference. To demonstrate the applicability of our model in this scenario, we constructed a human immune cell atlas from four studies of bone marrow^48^ and peripheral blood mononuclear cells (PBMCs)^49–51^. We then mapped a query PBMC dataset of samples from eight patients diagnosed with systemic lupus erythematosus whose cells were either untreated (control) or treated with interferon (IFN)-β, a potent cytokine inducing a strong transcriptional response in immune cells^52^. Successful mapping should align untreated cells to matching cell types in the healthy reference while preserving the strong effect of IFN-β. The expiMap model trained with GPs extracted from the Reactome^42,43^ pathway knowledgebase successfully mapped the query untreated cells to the healthy reference while forming clusters indicative of the IFN-β-treated cells (Fig. 2a).

**Extended Data Fig. 1:**
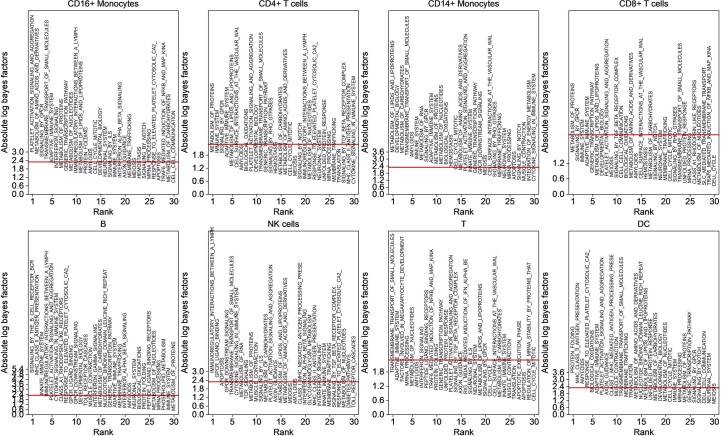
Differential GP analysis results for cell types from the integrated query and reference PBMCs with five datasets. Differential GP analysis results between cell types (one vs all test) for cell types in the query data. The x-axis is the ranking of GPs; the y-axis denotes the significance (absolute log-Bayes factor) of each GP. Source data

By testing between IFN-β and control conditions, we identified the top differential GPs, matching to previously reported GPs^53,54^ including IFN-related pathways (Fig. 2b), which also separates the control reference and query cells from stimulated query cells (Fig. 2c). Following up with a cell-type-specific analysis, we identified differential GPs across cell types (that is, one versus all) or cell-type-specific IFN-β effects (that is, IFN-β versus control within a cell type). In particular, we detected a group of population-specific GPs that separated one cell type from the rest (Fig. 2d, first row). The population-specific GPs can be used together with perturbation-associated GPs (that is, obtained from IFN-β query cells versus control cells in both query and reference for that cell type) to resolve the heterogeneity of cell state for that cell type (Fig. 2d, second row; for all cell types, see Extended Data Fig. 1 and Supplementary Figs. 1–3). We found that the general IFN GPs (for example, IFN signalling) are always induced in all cell types (Fig. 2e and Supplementary Figs. 2 and 3). In contrast, some GPs (for example, GPCR-related programs; their genes are provided in Supplementary Tables 1 and 2), including genes from the CXC chemokine family (for example, CXCL10), are present only in the myeloid lineage (for highlighted GPs, see Fig. 2e; for all extended figures, see Supplementary Figs. 2 and 3). Additionally, we detected carbohydrate metabolism activity in CD14^+^ and CD16^+^ monocytes and dendritic cells (DCs), and active amino acid metabolism in CD14^+^ monocytes after IFN-β stimulation (Supplementary Figs. 2 and 3). This is in agreement with previous observations in cancer and viral infection showing that amino acid, lipid and carbohydrate metabolic pathways contribute to the immune response^55,56^. Specifically, it is known that IFN-β engages with the amino acid metabolic pathway to produce polyamines and clear viral infections^57^. Still, a direct link to myeloid cells, as revealed by expiMap, has not been reported elsewhere.

**Extended Data Fig. 2:**
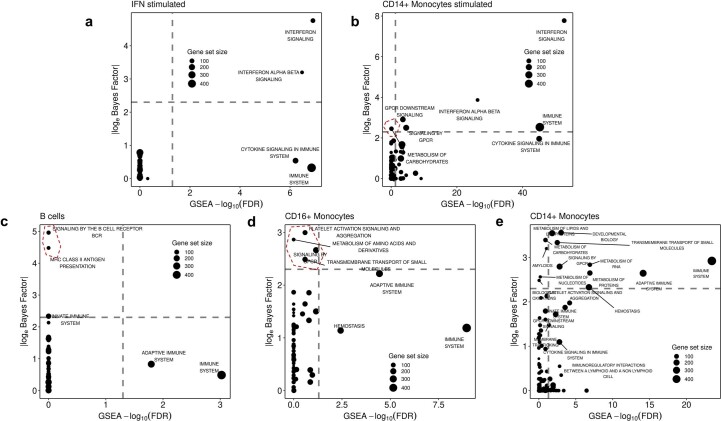
Comparison of expiMap Bayes Factors with GSEA –log_10(FDR). For comparison, we show Bayes factors from expiMap and FDR from fry. **(a)** Overall stimulated vs control tests (all cell types are pooled together). **(b)** Results for CD14^+^ monocytes stimulated vs control tests. **(c)** B cells vs the rest of the cell types. **(d)** CD16^+^ monocytes vs the rest of the cell types, **(e)** CD14^+^ monocytes vs the rest of the cell types. The x-axis shows the negative logarithm of the false discovery rate (-log10FDR) from fry; the y-axis shows the absolute value of the logarithm of the Bayes Factor (|loge(Bayes Factor)|) from the expiMap test. The size of the circles is proportional to the size of the gene set. We observed an overall agreement between expiMap and conventional GSEA results; however, expiMap detected more specific gene programs in some comparisons, while being computationally efficient with regard to runtime as the differential gene expression and enrichment testing steps no longer have to be repeated for every individual comparison. Source data

Differential expression analysis on atlases is challenging due to the complex experimental designs and the probable presence of nonlinear batch effects that cannot be modelled by linear approaches. Gene set enrichment analysis (GSEA) is a classical approach for inferring the activity of GPs and involves the sequential pipeline of differential expression analysis and gene set enrichment test. To evaluate the robustness of expiMap’s integrated GP test, we hence compared it with the classical GSEA via limma-fry^58,59^ (Supplementary Note 1 and Extended Data Fig. 2). In our comparisons, we observed that, unlike conventional gene set testing, which tends to detect general, non-specific terms, expiMap was able to identify specialized GPs. For example, in the B-cell population of both IFN-β-treated and control cells, expiMap detected B-cell receptor signalling and antigen presentation activity, which are more descriptive of B-cell biology than the general terms such as ‘adaptive immune response’ or ‘immune response’ that were found to be enriched in these cells by limma-fry (Extended Data Fig. 2c). We postulate that the increased variability in gene expression measurements hinders the detection of specialized biological signals by standard gene set testing on cell atlases. This indicates that expiMap can extract biologically relevant GPs from a single-cell atlas consisting of many datasets while accounting for technical variations such as batch effects, which may not always be feasible with existing pipelines, owing to the presence of nonlinear batch effects.

**Extended Data Fig. 3:**
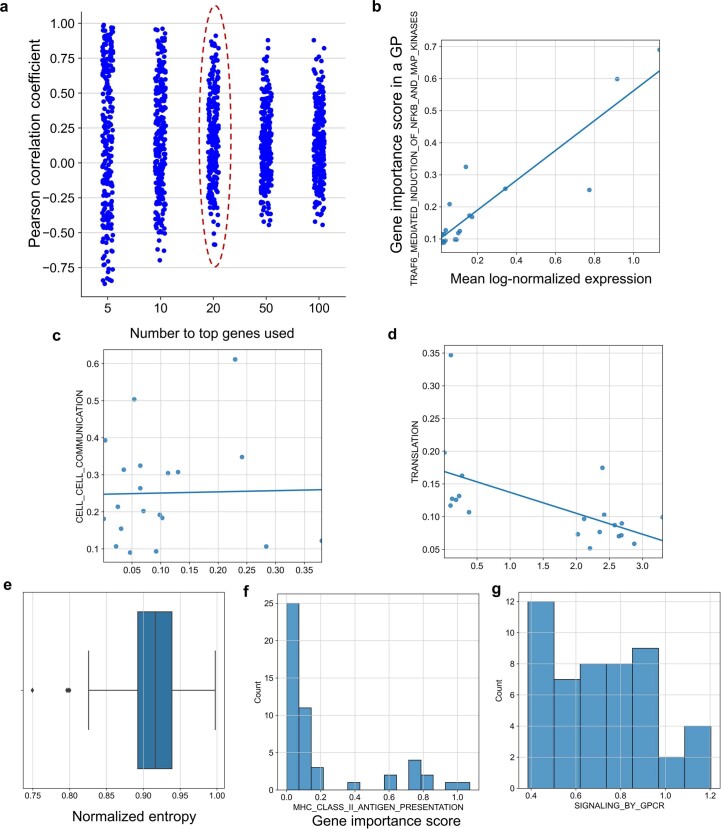
Analysis of GPs characteristics and their relationship to genes. **(a)** Distribution of correlation between mean expression value and gene importance score for each gene. X-axis: number of top-scored genes, and the Y-axis denotes the Pearson correlation of mean log-normalized expression for top n genes denoted in x-axis in each GP with their importance scores (each dot represents an active GP, n = 247). **(b-d)** - Scatter plots demonstrating the relation between mean gene expression and gene importance scores from expiMap for GPs, selected from the highlighted group in **(a)**. Example of a GP with a high positive correlation **(b)**, a GP with a correlation near zero **(c)**, and a GP with a negative correlation **(d)**. The X-axis shows the mean log-normalized expression of genes, y - gene importance score in a GP. Correlations are shown for the top 20 genes by gene importance scores. Each dot is a gene. **(e)** Box plot for the entropies of normalized importance scores of the top 50 genes for each active GP (n = 247) divided by the maximal entropy (of uniform distribution). The normalized entropy scale is from 0 (absolutely concentrated) to 1 (uniformly spread weights). Box plot statistics: lower quartile = 0.89, upper quartile = 0.94, median = 0.916, lower whisker = 0.83, upper whisker = 0.998, min = 0.75, max = 0.998. **(f)** Histogram of importance score for top 50 genes in MHC II ANTIGEN PRESENTATION, this GP has normalized entropy 0.799. **(g)** Histogram of importance score for top 50 genes in SIGNALING BY GPCR, this GP has normalized entropy of 0.988. Source data

**Extended Data Fig. 4:**
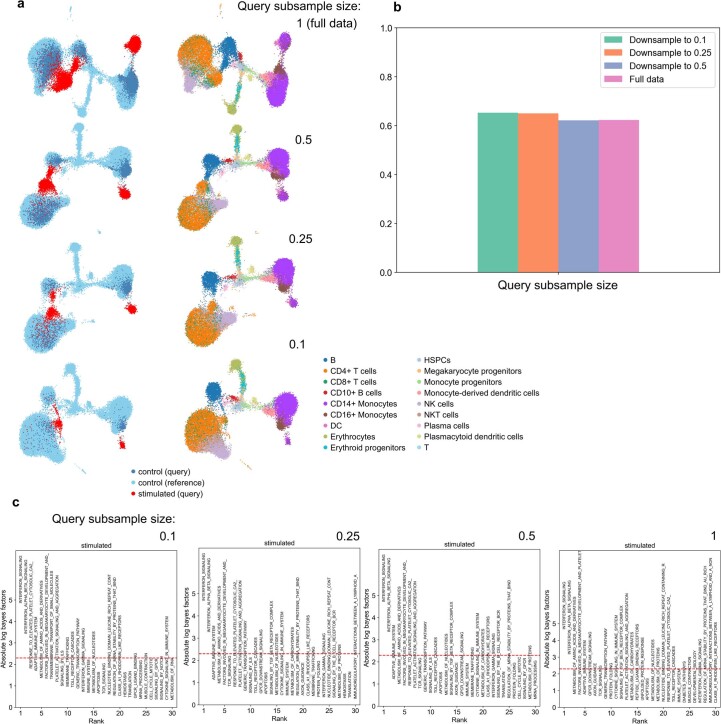
Results for expiMap model trained with downsampled query dataset (IFN-β dataset). **(a)** UMAP plot for the latent spaces of reference and downsampled query across different query dataset sizes. **(b)** Average of the integration metrics assessing the integration of reference and control query for all query sizes. **(c)** Results of the expiMap Bayes tests between control (reference + query) and IFN-β stimulated (in query). Source data

**Extended Data Fig. 5:**
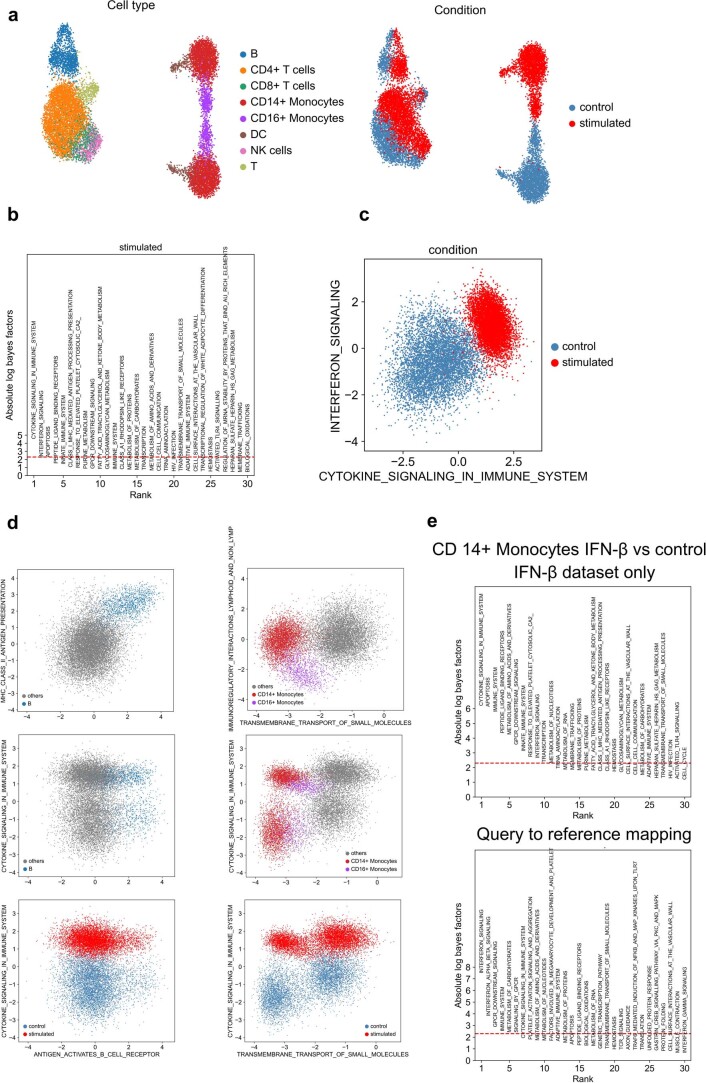
Results for expiMap model trained on IFN-β dataset alone. **(a)** UMAP plot of expiMap latent space control and IFN-β stimulated cells from eight patients (n = 13,576 cells), used before as a query dataset. Colors demonstrate cell type (left), and condition (right). **(b)** Differential GP analysis results between IFN-β stimulated and control cells. The x-axis shows the ranking of GPs; the y-axis denotes the significance (absolute log-Bayes factor) of each GP. **(c)** Scatter plot of the scores of the top two most significant expiMap GPs in **(b)**. Each dot shows the latent score of each cell. **(d)** Visualization of the scores for various GPs, delineating cell types or perturbation states for B cells and CD14 + /16+ monocytes. **(e)** Differential GP analysis results for CD14 + Monocytes only between IFN-β stimulated and control CD14+ monocyte cells, for both IFN-β dataset only and reference mapping. Source data

To further analyse the contribution of individual genes in each GP, we introduce the gene importance score: the absolute value of decoder weights for genes in GPs (see also Methods), which can measure the comparative importance of genes within each GPs. Using the importance score, we analysed the dependence between the expression levels of genes and their importance scores in various GPs (Supplementary Note 2 and Extended Data Fig. 3). We also confirmed the robustness of the model under different data query dataset sizes (Supplementary Note 3 and Extended Data Fig. 4). Finally, we compared reference mapping and individual analysis of query data by applying expiMap on IFN-β dataset alone and repeated analogous analysis as shown in Fig. 2. We found the results similar (Supplementary Note 4 and Extended Data Fig. 5).

### Biologically informed modelling improves the performance

**Fig. 3:**
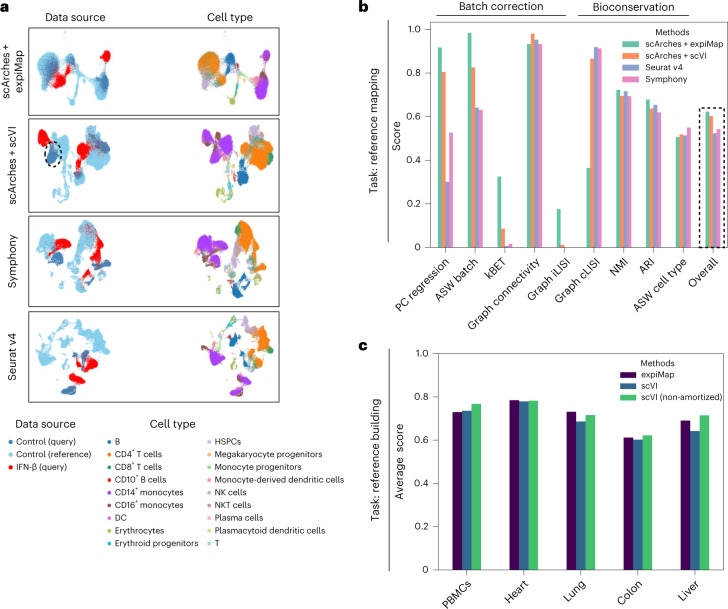
Domain awareness improves performance in downstream tasks. **a**, UMAP representation of integrated healthy immune reference with query interferon IFN-β data from eight patients for expiMap and existing reference mapping methods. Colours denote the data source and cell type. The dotted circle highlights query control monocytes that scArches + scVI failed to integrate into the control reference. **b**, Comparison of integration accuracy for mapping control query cells (excluding IFN-β cells) onto healthy atlases across different models. The metrics measure batch correction and bioconservation. The dotted line is the overall score calculated on the basis of the mean of all metrics. **c**, expiMap retains the expressiveness of an unconstrained reference model, as shown by the comparison of reference building performance through benchmarking in five different tissues, including PBMCs (*n* = 161,764, *n*_batches_ = 8), heart (*n* = 18,641, *n*_batches_ = 4), lung (*n* = 65,662, *n*_batches_ = 19), colon (*n* = 34,772, *n*_batches_ = 12) and liver (*n* = 113,063, *n*_batches_ = 14) across three different methods. The *y* axis is the average score of the nine metrics detailed in **b**. PC regression, principal component regression. Source data

As a means to benchmark the performance of expiMap’s reference mapping component, we compared it with scArches + scVI^7^, Seurat v4^8^ and Symphony^18^. Although expiMap and scVI both leverage scArches for reference mapping, scVI did not mix the untreated monocytes from the query data with healthy monocytes in the reference (dotted circle in Fig. 3a; for mixing of studies, see Supplementary Fig. 4), whereas expiMap successfully integrated them into the healthy reference (0.68 versus 0.47 average batch correction scores; see further for a description of the metrics) while preserving the effect of IFN-β treatment in cells that should not be integrated with the rest. We attribute this to the explicit incorporation of the IFN-β-related GPs in the expiMap model, which helps differentiate the perturbed and control states while resolving the transcriptional similarities between control cells, leading to better mixing of control states. We investigated this by removing the top five GPs obtained from the IFN-β versus control comparison (Fig. 2b) and retraining the model. We observed that this led to the incorrect mixing of control and stimulated cells with the reference (Supplementary Fig. 5). In this example, both scArches + scVI and expiMap had better performance than Seurat v4 and Symphony for integrating control query cells into control cells from the reference (Fig. 3b). We also quantitatively evaluated the integration of query control cells into the healthy reference using nine different metrics of biological preservation and mixing^60^.

**Extended Data Fig. 6:**
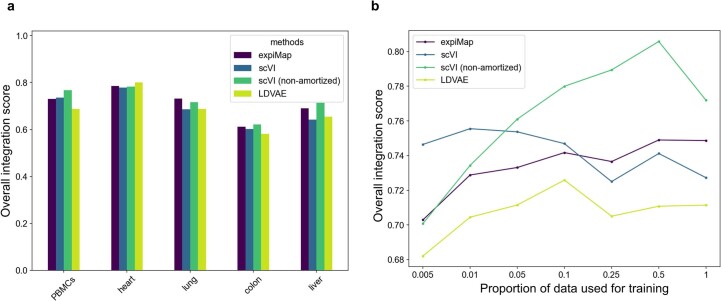
Benchmarking the reference building and assessing subsampling effects on data integration. **(a)** Comparison of the reference building performance by benchmarking across five different tissues, PBMCs (n = 161,764), heart (n = 18,641), lung (n = 65,662), colon (n = 34,772), and liver (n = 113,063), and four different methods. **(b)** Subsampling effect on data integration. The overall integration accuracy for different subsamples of PBMCs (n = 161,764)^8^ data across different models. The x-axis denotes the proportion of the data used for training each model; the y-axis is the overall average score across nine integration metrics measuring both biological preservation and batch removal, as introduced in Fig. 3b. Source data

We further benchmarked expiMap in de novo integration against scVI and non-amortized scVI (Fig. 3c), and linear-decoded variational autoencoder (LDVAE)^45^, a variation of scVI with a linear decoder (Extended Data Fig. 6a). Overall, we found that additional domain knowledge distilled into expiMap compensates for the lower model capacity compared with nonlinear models enabling it to achieve competitive performance (Supplementary Note 5). This is aligned with recent results^2,20^ demonstrating the improved performance of deep learning-based models by integrating domain knowledge into modelling.

### Learning new GPs

Leveraging domain knowledge is crucial for the rapid and interpretable analysis of new query datasets within the context of a reference atlas. However, domain knowledge is not always comprehensive, complete and up to date for a novel phenomenon (for example, a new disease). Thus, the ability to learn new GPs to analyse query data containing new variations, such as new states or cell populations, is pivotal. We address this by allowing expiMap to learn novel GPs associated with the query data that exist in the knowledge base but are not detected previously in the reference model, as well as de novo programs that are not described in the knowledge base (Methods).

**Fig. 4:**
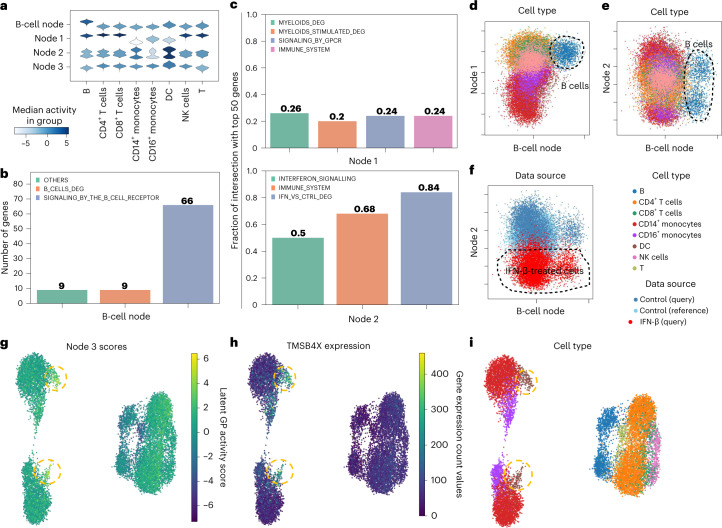
Learning new GPs from query data. **a**, Distribution of single-cell latent representation values across newly learned GPs across different query data cell types for query IFN-β-treated cells and control cells. **b**,**c**, Comparison of overlap of the most influential genes dominating the variance in newly learned constrained B-cell nodes (**b**) and unconstrained nodes (**c**) with genes in existing related GPs and top genes obtained from the differential testing analysis. The terms ‘MYELOIDS_DEG’ and ‘B_CELLS_DEG’ refer to genes obtained from one versus all Wilcoxon rank-sum tests in the query control cells for each population, respectively. The myeloid population consists of CD14^+^ monocytes, CD16^+^ monocytes and DC populations. ‘INF_VS_CTRL_DEG’ denotes differentially expressed genes comparing IFN-β-treated and control cells. The existing GPs for **c** are those with maximal overlap with at least 12 genes with newly learned GPs. **d**–**f**, Visualization of newly learned GPs (for cells from the reference and query datasets with cell types present in the query dataset) discriminating specific cell types and states from the rest, such as B cells and myeloids with the effect of IFN removed (**d**) or B cells with the effect of IFN preserved (**e**,**f**). **g**–**i**, UMAP of expiMap’s latent space for the query dataset coloured by node 3 latent representation values (**g**), TMSB4X gene expression counts (**h**) and cell types (**i**). The dotted circle highlights DCs. Source data

**Extended Data Fig. 7:**
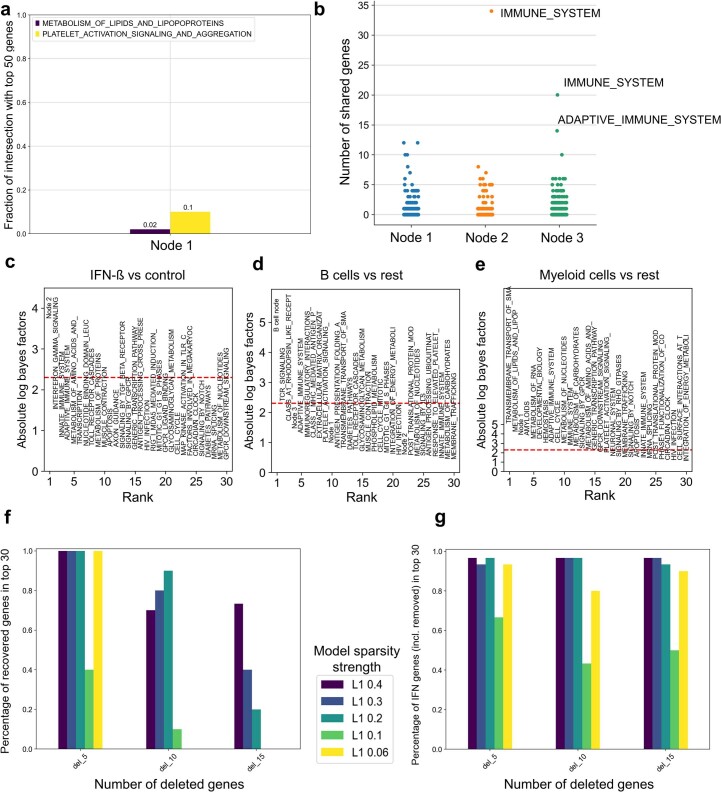
Learning new GPs for query data and benchmarking expiMap for enriching predefined GPs. **(a)** Comparison of the top influential genes dominating the variance in node 1 with genes from the top GPs for CD14 + /16+ monocytes from Fig. 2d**. (b)** Distribution of the number of overlapping genes between top 50 influential genes of new unconstrained nodes and the reference GPs for Fig. 4. Y-axis - number of shared genes between the top 50 genes of the unconstrained new nodes indicated in the x-axis and the reference GPs (n = 276). Each point is the number of shared genes with one reference GP. **(c-e)** Quantifying separations in Fig. 4. **(c)** Results of the differential expiMap test between IFN-β stimulated cells and control cells and B cells and the rest **(d)**. **(e)** Results of the differential expiMap test between Myeloid cells and the rest. In **(c-e)** x-axis - is the rank of the GP, y-axis - is the absolute value of the log Bayes score. **(f-g)** Benchmarking expiMap for enriching predefined GPs. The expiMap model was trained on the PBMCs dataset from Kang et al. (n = 13,576), with [CYTOKINE_SIGNALING_IN_IMMUNE_S’, ‘INTERFERON_ALPHA_BETA_SIGNALING’, ‘ANTIVIRAL_MECHANISM_BY_IFN_STI’, ‘INTERFERON_GAMMA_SIGNALING’, ‘IMMUNE_SYSTEM’] removed from GPs obtained from the Reactome database and only ‘INTERFERON_SIGNALING’ was kept for training. The x-axis shows the number of deleted top genes in the ‘INTERFERON_SIGNALING’ program, while the y-axis shows the percentage of those genes added to the top 30 genes in the ‘INTERFERON_SIGNALING’ program after training. The colors show the different values of L1 sparsity for each experiment. **(f)** The x-axis is the same as in **(g);** the y-axis demonstrates the percentage of the original interferon-related genes among the top 30 genes in the ‘INTERFERON_SIGNALING’ program after training. When the y-axis value is smaller than 1.0, it means that a 1–y percentage of false-positive genes was added to the GP. Source data

To evaluate the success of this strategy, we sought to remove GPs and cells containing information about IFN signalling and B cells during reference training and assess if the model could de novo learn GPs of that type if the query data contain B cells and IFN-β-treated cells. To this end, we removed the general IFN-related GPs, including IFN, IFN-*αβ* (and GPs containing a superset of those) and cytokine signalling in the immune system, from Reactome. We also removed B cells in the reference and the top two B-cell GPs containing information about B-cell receptor signalling and antigen presentation, as shown in Fig. 2d. Next, we trained the healthy reference PBMC model, as before, with the same studies as Fig. 2a, in which the model did not see GPs related to IFN pathway activity, B cells and their GPs in reference training. Further, we added a set of new nodes along with trainable weights at the query training stage; one was set with fixed gene membership to learn B-cell receptor signalling GP, and the other three were flexible and able to learn other variations in the data. In practice, we suggest initializing ten (as default) newly initialized unconstrained nodes for more complicated query datasets, as redundant nodes will be switched off (all decoder weights set to zero) by L1 regularization. Ideally, we would like the model to learn GPs containing information about new variations in the query. We examined the distribution of the latent space values across different cell types (Fig. 4a). The node constrained with the B-cell GP learned the variations specific to B cells (Fig. 4a, first row). The B-cell node had 84 active genes, of which 66 genes are from the B-cell receptor signalling GP (Fig. 4b). While expiMap learned the pre-defined GP, it also added nine B-cell markers (for the full gene list, see Supplementary Table 3) obtained using differential testing (Wilcoxon rank-sum test in scanpy^61^) owing to the soft membership features in the model that were not initially in the pre-defined GP, demonstrating the ability of the model to incorporate extra information and enrich incomplete domain knowledge (Fig. 4b). Further, by looking at distribution plots, one of the newly learned nodes after in query training displayed a different distribution for myeloid cells/lineage (denoted as node 1 in Fig. 4a). In contrast, other cells had uniformly similar values. Another node (node 2 in Fig. 4a) had a bimodal distribution across all cell types, suggesting that the variation between control and IFN-β-stimulated cells is captured. We then sought to uncover the variations in the de novo learned nodes by comparing the top 50 genes influencing that node using gene importance score (for details about gene importance score, see Methods) with GPs with a maximum number of overlapping genes and those from differentially expressed genes. We found that node 1 and node 2 learned variations related to myeloid and IFN-β (Fig. 4c). Specifically, node 1 is a new GP with minimal overlap with the top two previously identified programs (Extended Data Fig. 7a). This newly learned program also had a maximum gene overlap of 24% with the top 50 genes influencing the GP with other existing GPs in Reactome (GPs that had maximum gene overlap are shown on the first row in Fig. 4c). The full distribution of overlaps between reference GPs and new unconstrained GPs is shown in Extended Data Fig. 7b. The only significantly overlapping GP is IMMUNE_SYSTEM, a very large and general GP with 491 genes. This demonstrates that the model learned a new program distinguishing myeloid cells from other cell types. Node 2 also captured the program describing the IFN response observed only in the query data. When plotted against each other, we observe the separation of B cells and myeloid cells (Fig. 4d), IFN-β-treated cells and B cells (Fig. 4e,f). We also quantified GPs specificity with classification and statistical metrics corroborating visual and qualitative comparisons (Extended Data Fig. 7c–e and Supplementary Table 4). Finally, the last node (node 3 in Fig. 4a) was de-activated for most of the training but started to capture the signal related to DC cells (Fig. 4g–i). The most important gene for node 3 is TMSBX4 (for a ranked list of important genes in node 3, see Supplementary Table 5), which has higher expression levels for DC cells (Fig. 4h). The scores of node 3 also have comparatively higher values for DC cells (Fig. 4g).

We further confirmed the independence of newly learned GPs (Supplementary Note 6 and Supplementary Table 6). Finally, we showed our model is robust in learning new GPs and existing GPs across different data subsampling scenarios and model hyperparameters (for detailed analysis, see Supplementary Note 6 and Supplementary Tables 7–12). Overall, we demonstrated that expiMap can learn pre-defined GPs not in the reference GP matrix for populations present only in the query data during query training while having the ability to enrich the pre-defined GPs with new genes (see also Supplementary Note 7 and Extended Data Fig. 7f, g), not in the program. In addition, we demonstrated that expiMap is not restricted to pre-defined GPs and can learn de novo GPs without any user supervision or prior knowledge.

### Interpreting treatment responses of patients with COVID-19

**Fig. 5:**
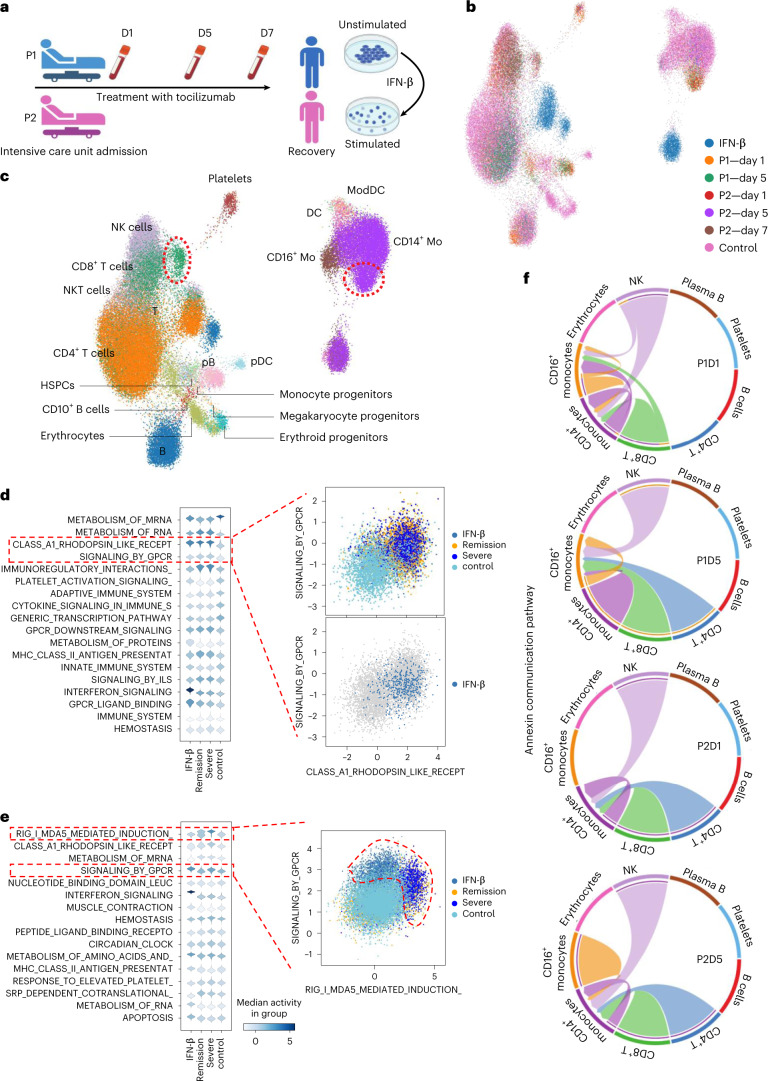
expiMap analysis highlights the importance of the annexin gene family communication pathway during moderate and severe COVID-19. **a**, Illustration of the integrated datasets from PBMCs of healthy controls, patients with severe COVID treated with tocilizumab, and patients in the remission stages, and in vitro IFN-stimulated PBMCs. Figure made with BioRender. **b**, Integrated manifold using expiMap showing combined healthy PBMCs (*n* = 32,484), two query datasets including two patients with COVID-19 (*n* = 18,752) and the IFN-β dataset (*n* = 13,576) (ref. ^18^). **c**, Detailed cell type annotation of the integrated PBMC datasets. Red circles highlight cells not merged with the healthy PBMC cell atlas. ModDC, monocyte-derived dendritic cells; CD14^+^ Mo, CD14^+^ monocytes; CD16^+^ Mo, CD16^+^ monocytes; pDC, plasmacytoid dendritic cells; pB, plasma B cells. **d**,**e**, Distribution plots for differential GP activities were obtained using expiMap for CD8^+^ T cells and CD14^+^ monocytes, highlighting the antiviral transcriptional programs for *RIG-I*/*MDA5* and *GPCRs* in each population. ILS, interleukins. Scatter plots are latent GPs representations of highlighted GPs for each cell type. **f**, Annexin communication pathways in different stages of COVID. In the severe stage (P1D1), CD14^+^ and CD16^+^ monocytes participate in a dynamic communication activity via annexins with NK and CD8^+^ T cells. This circuit converges to focused signalling to CD16^+^ monocytes during COVID remission (P1D5). In P2, CD14^+^ monocytes receive focused annexin signalling from NK, CD8^+^ and CD4^+^ T cells in the severe stage (P2D1), and later converge to signalling to CD14^+^ monocytes from the same lymphoid effectors during remission (P2D5). Source data

To demonstrate the medical use of interpretable atlas querying, we focused on the cellular response to infection during coronavirus disease 2019 (COVID-19) and the effect of immunosuppressive interventions. We leveraged the integrated immune PBMC atlas to map IFN-β dataset (as in Fig. 2a) and a new dataset from two patients (P1 and P2) at different COVID stages (severe disease and during the remission process: D1, severe COVID-19 on day 1; D5 and D7, remission on days 5 and 7, respectively). Both patients were treated with tocilizumab, an immunosuppressive drug targeting the interleukin-6 receptor^62^. The integrated dataset (Fig. 5b,c) was re-annotated using canonical markers identifying 20 cell states from the myeloid and lymphoid compartments, including rare populations such as megakaryocytes and erythroid progenitors, as well as a population of *CD10*^*+*^ B cells (Fig. 5c). From the integrated embedding produced by expiMap, we could observe that some cellular states are associated with disease severity, which may be related to differences in the cellular response to tocilizumab.

Our analyses pointed us towards CD8^+^ T cells and monocytes (Fig. 5c) in both severe and remission stages that did not integrate into the healthy reference, unlike other populations from the same patients. We investigated this by performing a differential GP test between severe query cells and control cells to identify GPs that could explain this separation. We identified transcriptional programs of antiviral response at different clinical stages of COVID-19 and in specific PBMC cell types. Pathogen recognition receptor (PRR) *RIG-I*/*MDA5* and GPCR pathways displayed differential behaviour in CD8^+^ T cells (Fig. 5d) and CD14^+^ monocytes (Fig. 5e) in severe COVID-19 (D1) and during remission (D5 and D7). *RIG-I*/*MDA5* and GPCR pathways initiate the innate immune response and modulate the adaptive immune responses during viral infections^63^ and are reported to coordinate the inflammatory dynamics during COVID-19 (refs. ^64,65^). These findings suggest that a complex cellular communication circuit may be differentially activated in both patients and may be related to the differences in treatment response at the cellular level.

Next, we estimated underlying cellular communication pathways using CellChat^66^ and compared them at different clinical stages in our integrated Immune atlas. This analysis revealed that the annexin pathway displayed differential transcriptional behaviour in the severe and remission stages of P1 and P2, involving CD14^+^ and CD16^+^ monocytes, natural killer (NK) cells and CD8^+^ T cells (Fig. 5f and Supplementary Fig. 6). Annexins are structural proteins that participate in the regulation of inflammatory responses and homeostasis^67^ and have been associated with disease severity in COVID-19 (refs. ^68,69^). In this circuit, CD14^+^ and CD16^+^ monocytes show the potential to receive signals from NK cells and CD8^+^ T cells for P1D1. In P1D5, the annexin pathway switches completely to signalling between CD16^+^ monocytes and CD4^+^ T cells. In stark contrast, P2D1 is characterized by the annexin pathway focusing on CD14^+^ monocytes, which continues throughout the remission stage (P2D5), with the addition of CD16^+^ monocytes persisting towards D7 of remission (P2D7) (Supplementary Fig. 6).

**Extended Data Fig. 8:**
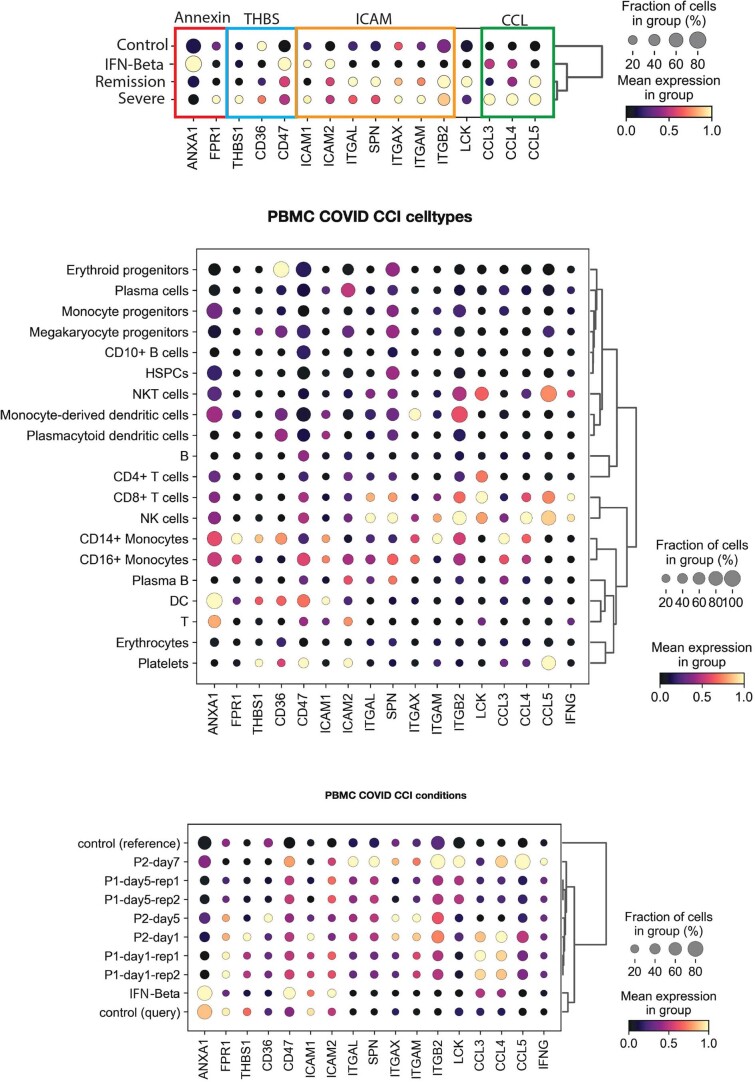
Transcriptional activity of cellular communication circuits occurring in severe COVID. Transcriptional activity of genes associated with cell-cell communication pathways and interferon-gamma *(IFNG)* in different cell types and conditions. The cell-cell communication pathways represented are annexins (*ANXA1, FPR1)*, THBS *(THBS1, CD36, CD47)*, ICAM *(ICAM1, ICAM2, ITGAL, SPN, ITGAX, ITGAM, ITGB2)*, LCK, and CCL *(CCL3, CCL4, CCL5)*. Source data

Although expression levels of annexins have been described as biomarkers for the prediction of disease severity^69^, our analysis using expiMap is the first to describe the expression of ligand–receptor pairs from the annexin pathway at the cellular level with the potential to interact between monocytes (CD14^+^ and CD16^+^), NK cells and T cells in COVID. The differences observed between patients in the expression of annexin-related interaction circuits may be related to the capability of viral clearance in each patient^62^ and the early expression of *FPR1* by CD16^+^ monocytes, which is associated with the early detection of pathogenic molecules and tissue damage^70^. Interestingly, our analysis shows the expression of *IFNG* for P2D7 by NK and CD8^+^ T cells (Extended Data Fig. 8), which may indicate a more complex antiviral response than in P1, independent of the symptomatic resolution attained by tocilizumab. Moreover, when contrasting our results with the annexin circuit in the data from IFN-stimulated cells, we observed that the inferred cell–cell interactions using the annexin pathway were dominated by the expression of *ANXA1* in DCs rather than *FPR1* in CD14^+^ monocytes (Extended Data Fig. 8). Our results do not illustrate the same circuit; however, this may indicate a lung-specific interaction operating in the lungs after the monocytes migrate to the affected lung tissue.

Although both these patients recovered after treatment with tocilizumab, clinical studies demonstrate that this behaviour is not consistent, and other factors, such as tocilizumab posology, may affect the clinical outcome^71^. At the cellular level, expiMap identifies transcriptional and cell–cell interaction circuits with the potential to be druggable, such as RIG-I/MDA5 and annexins, to help suppress cytokine storm syndrome in patients with COVID-19, which results in hospitalization.

### expiMap resolves disease heterogeneity in Pancreas

**Fig. 6:**
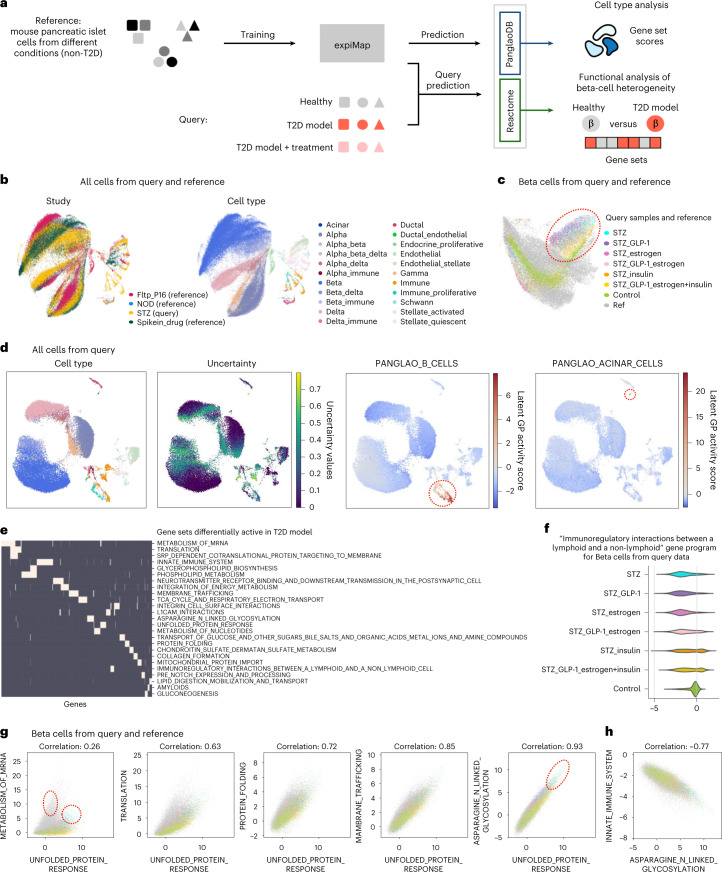
Reference mapping of pancreatic islet cells using expiMap. **a**, Pancreatic islet cell analysis. The expiMap model was trained on heterogeneous non-T2D mouse pancreatic islet cells from different datasets. A dataset containing healthy and treated T2D-model cells was mapped to this reference. expiMap was trained with GPs from PanglaoDB to evaluate cell type annotation and scores from Reactome to determine metabolic differences between healthy and T2D-model beta cells. **b**, expiMap-integrated UMAP coloured by dataset shows three reference Pancreas datasets (45,178 cells) and one query dataset (36,899 cells). **c**, Healthy and T2D-model beta cells from the reference and query separate on UMAP. **d**, The expiMap score for immune B cells highlights a subpopulation of cells previously annotated under the umbrella term of immune cells. The score for acinar cells helps annotate the small acinar cell type cluster, which was not annotated in automatic cell type transfer owing to low classifier certainty. **e**, Low redundancy of the top differential Reactome pathways between healthy and T2D-model query beta cells. Genes (columns) associated with each GP (rows) are marked in white; the absence of a gene in a GP is indicated by dark colour. The displayed matrix was clustered both by genes and GPs. **f**, The immune interaction GP scores in insulin-treated T2D-model beta cells from the query are bimodally distributed. **g**,**h**, Beta-cell scores of selected GPs differentially active in T2D-model beta cells. Comparison of UPR and protein synthesis and processing GP activites (**g**) and comparison of *N*-linked glycosylation and immune GP activity (**h**). Legend is shown in **c**. On the first subplot, circles mark the T2D-model population with relatively high scores in UPR and mRNA metabolism compared with healthy control from the query, whereas other non-T2D cells from the reference show high mRNA metabolism without a high UPR score. The circle indicates the T2D-model population with extreme UPR and asparagine *N*-linked glycosylation scores. ref: reference datasets, other samples are from the query; STZ: streptozotocin T2D model; STZ_GLP-1: STZ treated with GLP-1; STZ_estrogen: STZ treated with oestrogen; STZ_GLP-1_estrogen: STZ treated with GLP-1-oestrogen conjugate; STZ_insulin: STZ treated with insulin; STZ_GLP-1_estrogen+insulin: STZ treated with GLP-1-oestrogen conjugate and insulin; control: healthy control. Source data

**Extended Data Fig. 9:**
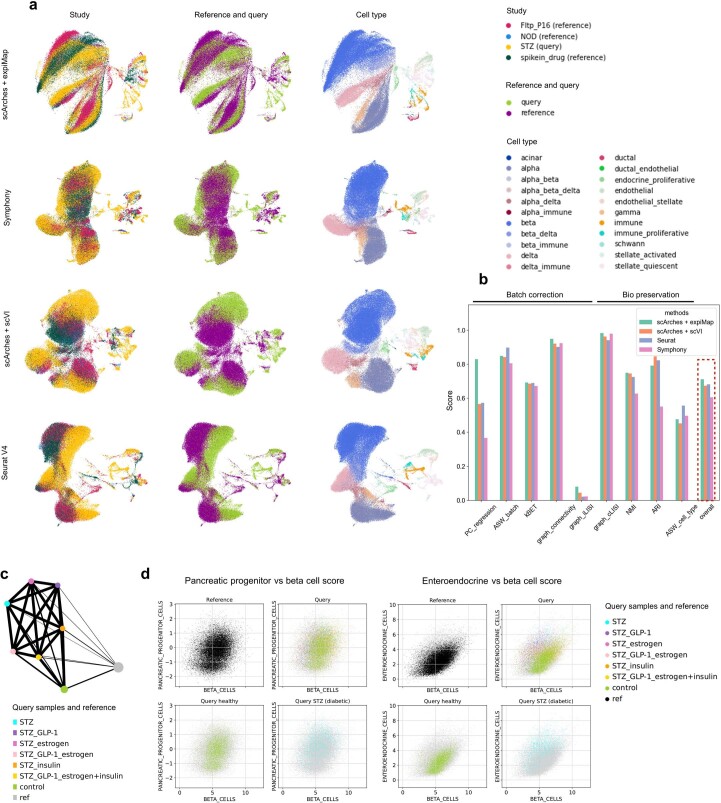
Comparison of integration results across different integration methods. **(a)** UMAPs of integrated embeddings obtained with different integration methods, **(b)** comparison of integration quality across methods, **(c)** PAGA of integrated beta cells indicates that the connection of reference cells with query control cells is the strongest, the connection of T2D-model query cells treated with insulin is moderate, and the connection with other T2D-model cells is the weakest. **(d)** expiMap term scores in beta cells correspond to the known loss of beta cell identity, dedifferentiation, and transdifferentiation in diabetes. Left, loss of beta cell identity (x-axis) vs dedifferentiation-related (y-axis) expiMap terms; right, loss of beta cell identity (x-axis) vs transdifferentiation-related (y-axis) expiMap terms. Source data

As a final use case, we asked whether expiMap could assist with interpretable cell type annotation and the analysis of cell state heterogeneity. We used expiMap to integrate three non-type 2 diabetes (T2D) pancreatic datasets^72–75^ (Methods and Supplementary Note 8) differing in multiple biological factors, including sex, age and stress status, using PanglaoDB marker sets to enable cell type identification^76,77^ and Reactome pathways to identify molecular processes^78^ differentially active between biological conditions. Before integration, we removed immune cells from the reference to assess whether new cell types in the query could be successfully integrated. We projected a new dataset (query) that included healthy and T2D cells into this reference (Fig. 6a,b). On the integrated embedding (Fig. 6b), a separation between studies is observed. This is expected due to biological differences between the integrated mouse models, such as disease state and age. We also performed integrations with scArches + scVI, Seurat V4 and Symphony (Extended Data Fig. 9a) and assessed the integration quality using scIB metrics (Extended Data Fig. 9b), showing that expiMap is one of the top-performing methods.

Next, we automatically transferred cell type annotations from reference to query (Fig. 6d, Supplementary Fig. 7 and Methods). Analysing expiMap-generated scores of pancreatic cell type-associated PanglaoDB GPs (Supplementary Fig. 7c) helped with the annotation of ambiguous cell clusters (Supplementary Fig. 7b). For example, expiMap scores helped to resolve potential doublets (for example, immune–endocrine doublets) and small cell populations (for example, acinar cells) that were marked as unknown or wrongly annotated (Fig. 6d and Supplementary Fig. 7b,c). As automated cell type annotation methods often produce unreliable results in challenging cell populations, such as doublets, rare cell types or transitional cell states, manual assessment of marker expression is still required. However, expression can be affected by batch effects^76^, while expiMap scores are directly comparable. Furthermore, when specific cell types are missing from the reference, the annotation transfer cannot be performed, such as for the immune cells that were present only in the query (Fig. 6a and Supplementary Fig. 7a). In such a case, expiMap enables GP-enrichment analysis to provide insights into cell types. Similarly, expiMap scores can resolve coarsely annotated cell types. We show that the GP cell type scores for the immune cell subpopulations (Fig. 6d) provide similar information as the manually curated markers (Supplementary Fig. 8). As online marker databases often contain multiple putative markers, often of insufficient quality, manual selection of markers becomes challenging^79,80^. Indeed, we tried to use the top PanglaoDB markers for B-cell annotation (*Ebf1*, *Cd74* and *Cd52* out of 110 markers). However, they lacked sufficient specificity and sensitivity, while expiMap score based on all PanglaoDB markers correctly annotated B-cell lineage, corresponding to the B-cell lineage marker *Cd79a*^81^ as well as non-activated B cell (*Cd19* and *Ms4a1*) and activated plasma B cell (*Jchain*) markers (Supplementary Fig. 8). This can be explained by the prioritization of informative genes within expiMap for data-specific cell reconstruction to create a single batch-corrected GP score, helping to resolve ambiguities and challenges of automatic reference-based classifiers^79^. We also show that expiMap scores explain why the diabetes model and healthy beta cells do not overlap in integration, as indicated by differential activity of identity and maturation GPs (Fig. 6c, Extended Data Fig. 9 and Supplementary Note 8).

To search for molecular changes between the healthy control and T2D-model beta cells from the STZ study, we used the expiMap Bayes test with the Reactome GPs (Supplementary Table 13). We demonstrate that there is only a small overlap between the genes of the enriched GPs (Fig. 6e), simplifying the interpretation. We observed differences in energy metabolism, unfolded protein response (UPR) and islet communication, as previously reported in the original study (Supplementary Note 9 and Supplementary Table 14). To identify whether the enriched GPs separate cells into multiple populations within samples, we analysed the distributions of Reactome GP scores. The score of interactions between lymphoid and non-immune cells (Fig. 6f) had a multimodal distribution within T2D-model beta cells treated with insulin, potentially indicating the presence of multiple cell states within individual samples. For scores from other enriched GPs, we did not observe such distinct multimodal patterns within individual samples.

One of the key dysfunction processes in T2D, also identified in our enrichment analysis, is the UPR, which results from pro-insulin synthesis rate that exceeds the protein processing capacity of cells, leading to beta-cell dysfunction and death^82,83^. Thus, we compared scores of enriched GPs associated with UPR and protein synthesis and processing across individual cells (Fig. 6g). As expiMap produces batch-corrected GP scores we could also perform cross-study comparison with reference. We observed a high correlation between the UPR and asparagine *N*-linked glycosylation GP scores (absolute correlation coefficient of 0.93) across all datasets with extreme GP scores in T2D-model cells (Fig. 6g). An increase in *N*-linked glycosylation had been previously implicated in diabetes, although the regulatory background is not clear^84,85^. We further support the implication of N-linked glycosylation in T2D and its potential association with immune response (Fig. 6h, Supplementary Fig. 9 and Supplementary Note 10). We also assessed how multiple genes contribute to GP scores and how GP rather than gene-level comparison reduces noise (Supplementary Note 10).

**Extended Data Fig. 10:**
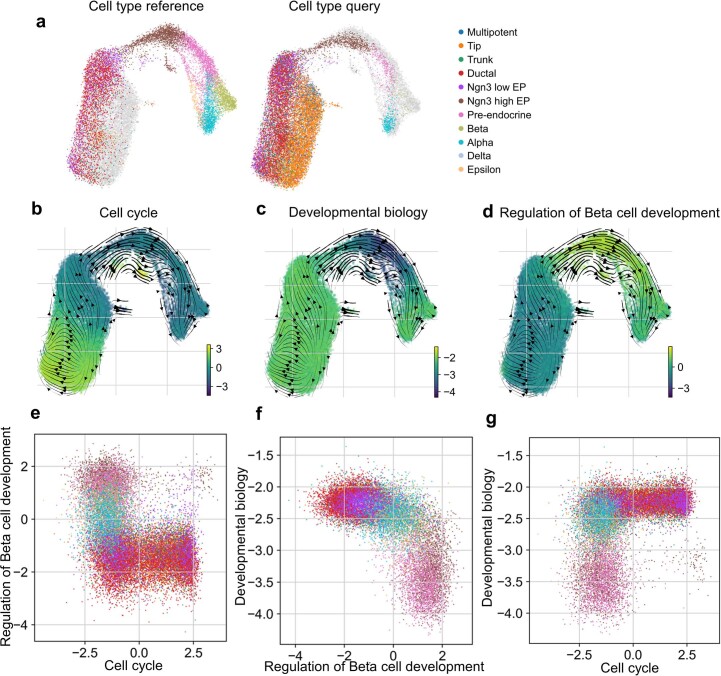
Developmental dataset from mouse endocrinogenesis. **(a)** UMAP plot of the latent space of expiMap for mouse endocrinogenesis dataset (n = 25,919) when mapping embryonic day (E) 12.5 and E13.5 to reference containing population from E14.5 and E15.5 colored by cell types. **(b)** UMAP plot of the latent space colored by the latent scores corresponding to the Reactome GP Cell Cycle (**b**), Developmental Biology (**c**), and regulation of Beta cell development (**d**). Developmental Biology and Regulation of Beta cell development GPs were inferred from differential GP analysis across cell types. **(e-g)** Scatter plots of different latent GP scores highlight the separability of different populations. Source data

Finally, we applied expiMap on another Pancreas dataset capturing mouse endocrinogenesis^86^ to demonstrate the model’s applicability on continuous developmental processes (Supplementary Note 11 and Extended Data Fig. 10). Overall, our results demonstrate that the expiMap GP activity analysis captures a complex differentiation and perturbation process in Pancreas.

## Discussion

We introduced expiMap for interpretable single-cell reference mapping. Our model embeds domain knowledge in the form of GPs into the deep learning architectures used for reference mapping and can further complement these GPs with newly discovered unconstrained GPs for query datasets. The interpretability of the model allows the users to generate immediate inferences about the query once mapped to a reference within the context of GPs. This contrasts with the existing analysis pipelines, which involve multiple steps and, without end-to-end learning, necessarily aggregate processing errors from previous steps. Interestingly, in a comparison across five different organ atlases, we found that the constrained expiMap model did not lose expressiveness versus an unconstrained conditional variational autoencoder model; indeed, prior constraints appeared to improve reference mapping and de novo data integration performance, confirming the earlier concepts of adding ‘differentiable programs’^20^. Through various applications, we demonstrated the interpretability of the model.

Reference mapping with expiMap provides a new perspective on data integration and reference mapping. In scenarios with significant differences in the datasets, such as cross-species mapping, the query data might not be fully aligned in the reference owing to the substantial biological and technical differences dominating the overall representation obtained by existing methods. This phenomenon makes it challenging to distinguish shared and unique signals between datasets. expiMap enables the integration of datasets along the axes of variations explained by a single or multiple GPs, where the datasets share variations and are mixed. This mixing stems from the commonality of the datasets in those programs. Such insights could not be obtained by, for example, looking at the overall uniform manifold approximation and projection (UMAP), which would be influenced by all genes, might be misleading and could obscure such commonalities. As we demonstrated when mapping COVID-19 patient data, CD8^+^ T cells from patients with COVID-19 were separate from IFN-β-treated CD8^+^ T cells in the global representation obtained from all GPs in UMAP (Fig. 5b,c). At the same time, they are integrated within specific GPs, capturing shared signals in two different cell states (Fig. 5d). Overall, expiMap can provide more insights into data integration by contextualizing it within GPs.

Our model leverages domain knowledge to improve the interpretability of deep learning models useful for single-cell genomics. With increasing availability^87,88^ of curated domain knowledge, expiMap can be trained on multiple databases while pruning irrelevant information. However, selecting the relevant knowledge to include in the model can affect the model’s performance. As we demonstrated, including IFN-related knowledge can improve the performance in reference mapping (Fig. 2), while excluding it can lead to poor mapping of the query (Supplementary Fig. 4). Another limitation concerns the interpretation and validation of newly learned GPs that capture new variations in the query data. As we demonstrated, looking at distribution plots and visualizing the embedding can decipher the variations. However, the validation at the gene level requires further expert knowledge for each biological system. Another limitation is the modelling hierarchies in unsupervised settings, starting from single genes to GPs and to higher-level biological processes. Previous work, such as knowledge-primed neural networks^89^, P-net^26^ and visible neural networks^90^ employed hierarchical modelling, but in supervised settings, to predict tumour type or cell states. Using a similar strategy in an unsupervised model would add another layer of analysis to mapping data, not only to GPs but also to biological processes, and potentially improve the performance. A final limitation of general deep learning models may be data hunger. To determine the sensitivity of our model to dataset size, we trained models of increasing quality by incrementally including more training samples in the reference building task (Extended Data Fig. 6b). We observed that expiMap outperformed the linear baseline of a non-biologically informed linear decoder model (LDVAE) in a low-data regime. The more complex non-amortized scVI achieved the best results with increased number of training samples, while expiMap outperformed scVI and LDVAE. Overall, these results suggest that incorporating prior knowledge leads to more sample-efficient learning in the presence of fewer samples than non-biologically informed models with similar complexity (for example, LDVAE). Further, when more training samples are available to learn GP activities efficiently, expiMap performs with complex nonlinear models.

Although we demonstrated expiMap by using single-cell RNA sequencing data, the model is naturally extendable to multimodal^91–93^ datasets. Recent technological advances in single-cell biology allow the simultaneous capture of chromatin accessibility, gene expression and protein levels in single cells^4^. This makes it possible to learn the hierarchy of connected representations by distilling domain knowledge about regulatory elements, transcription and translation, covering multiple cellular processes into the representation learning methods. Another potentially exciting direction is the combination of the expiMap architecture with in vitro perturbation modelling approaches^5,6,33^ to model in vitro perturbations of GPs. Finally, given the availability of spatial transcriptomics data^94^, it is possible to adapt expiMap to include information about cell-to-cell communication^95^ in the learned representations to gain further insights into cellular communications and signalling.

Researchers in the field of single-cell genomics are moving towards using reference mapping to analyse new query datasets. We envision that expiMap will further advance the applicability of reference mapping methods by bringing a new layer of interpretability and mechanistic understanding to integrative single-cell data analysis facilitating biological hypothesis generation and discovery.

## Methods

### expiMap model

Our model builds upon the framework of (conditional) variational autoencoders^34,96^. The log-likelihood of the data for expiMap can be written as

1 $${{{\mathrm{log}}}}p_\theta \left( {{\mathbf {X}}|W,{\mathbf {C}}} \,\right)p\left( W \,\right) = {{{\mathrm{log}}}}{\displaystyle\mathop{\int}\limits_{\mathbf {Z}}} {p_\theta } \left( {\mathbf {X}}|{\mathbf {Z}},W,{\mathbf {C}}\, \right)p\left( {\mathbf {Z}} \,\right)p\left( W \,\right)d{\mathbf {Z}}$$

2 $$p_{\theta} \left( {\mathbf{X}|\mathbf{Z},W,\mathbf{C}}\, \right) = {{{\mathrm{NB}}}}\left( {g\left( {\left[ {\mathbf{Z},\mathbf{C}} \right]\left[ {W,L} \right]^{\mathrm{T}}} \right),\mathbf{CD}} \right),$$

where $g\left( x \right) = {{{\mathrm{softmax}}}}\left( x \right) \times S$ is a softmax function that is multiplied by the library size *S* of each cell. Alternatively, *g*(*x*) could also be a softplus or exponential function. Further, **X** is a random variable representing gene expression, **C** indicates conditions (for example, batch ID) and $p_\theta \left( {\mathbf X\mid \mathbf Z,W,\mathbf C} \right)$ is the output distribution, also called a decoder in the setting of variational autoencoders, used to model **X** given the latent variable **Z***.*

${{{\mathrm{NB}}}}\left( { \cdot , \cdot } \right)$ in equation (2) denotes the mean and dispersion parametrized negative binomial distribution, $\left[ { \cdot , \cdot } \right]$ means a column-stacked matrix, *W* and *L* are matrix parameters for latent variables **Z** and one-hot encoded conditions **C**, respectively; and *D* is a matrix of condition-specific dispersion parameters for each gene. *W* is a *n* × *m* matrix with *n* corresponding to the number of genes and *m* corresponding to the number of GPs provided as an input.

The prior *p*(*w*) in equation (1) is defined as:

$$\begin{array}{l} {{{\mathrm{log}}}}p\left( {W_{:,\,j}} \right) = {{{\mathrm{log}}}}\mathop {\int}\limits_{\tau ^2} p \left( {W_{:,j}|\tau ^2} \right)p\left( {\tau ^2|\alpha } \right)d\tau ^2 = - \alpha \left\| {W_{:,j}} \right\|_2,\\{{{\mathrm{log}}}}p\left( W \,\right) = - \alpha{\displaystyle{ \mathop {\sum}\limits_j}} {\left\| {W_{:,\,j}} \right\|_2} \\p\left( {W_{:,j}|\tau ^2} \right) = {{{\mathcal{N}}}}\left( {0,\tau ^2I} \right),p\left( {\tau ^2|\alpha } \right) = {{{\mathrm{Gamma}}}}\left( {\frac{{n + 1}}{2},\frac{{\alpha ^2}}{2}} \right)\end{array}$$

The constants were omitted because they do not affect the optimization. We use a hierarchical Bayesian prior on the columns of *W* with the parameter *τ*^2^ integrated out as in oi-VAE^97^, resulting in the lasso regularization term. The lasso regularization allows the model to de-activate the GPs that do not contribute to the reconstruction loss in the model. *α* is a hyperparameter specifying the strength of the group lasso regularization.

The evidence lower bound (ELBO) is a part of our total loss to train the model. During the model training, the posterior distribution $p_{\theta} \left( {\mathbf Z|\mathbf X,\mathbf C} \right)$ is approximated by the variational distribution $q_\phi \left( {\mathbf Z|\mathbf X,\mathbf C} \right)$, which includes a deep neural network parameterized with *ϕ*; it is also called an encoder. The ELBO can be written as:

3 $$\begin{array}{l} {\mathrm{log}}{\displaystyle{\mathop {\int}\limits_{\mathbf Z}}} {p_{\theta} } ( {\mathbf{X}|\mathbf{Z},W,\mathbf{C}} )p(\mathbf{Z})p(W)d \mathbf{Z} \ge {\displaystyle{\mathop {\int}\limits_{\mathbf Z}}}{q_{\phi} } ( {\mathbf{Z}|\mathbf{X},\mathbf{C}} ){\mathrm{log}}\frac{{p_{\theta} ( {\mathbf{ X}|\mathbf{Z},W,\mathbf{C}} )p( \mathbf{Z} )p( W\, )}}{{q_{\phi} ( {\mathbf{Z}|\mathbf{X},\mathbf{C}} )}}d\mathbf{Z}\\={\Bbb E}_{q_{\phi} ( {\mathbf{Z}|\mathbf{X},\mathbf{C}} )}\left[ {{{\mathrm{log}}}}p_{\theta} \left( {\mathbf{ X}|\mathbf{Z},\mathbf{W},\mathbf{C}} \right) \right] - {\Bbb K}{\Bbb L}\left( {q_{\phi} \left( {\mathbf{Z}|\mathbf{X},\mathbf{C}} \right)\parallel p\left( \mathbf{Z}\, \right)} \right)+ {{{\mathrm{log}}}}p\left( W\, \right)\\={{{\mathrm{ELBO}}}}\left( {\theta ,\phi ,W} \right)\end{array}$$

where *θ* and *ϕ* are parameters of the decoder and the encoder, respectively.

### GP matrix

We use tab-delimited text files where the rows represent gene sets as an input to construct masks for *W* (see the previous section). The first column is reserved for the name of the gene sets and the other columns should contain the names of genes. Gene matrix transposed files (.gmt file format) could be directly used in our API as an input.

A database could be also passed to the model in the form of a binary matrix *B* with columns corresponding to GPs and rows corresponding to genes, with $B_{i,j} = 1$ if the *i*th gene is in the *j*th GP and 0 otherwise. Such a matrix is actually always constructed from the files described above before passing to the model. We refer to matrix *B* as the GP matrix.

### Defining hard/soft gene membership

The decoder network in equation (2) consists of a linear layer $\mathbf H = \left[ {\mathbf Z,\mathbf C} \right]\left[ {W,L} \right]^T$, in which the output is then transformed to a negative binomial means by the nonlinear function *g*(*H*). The GP matrix *B* specifies GPs and the gene memberships for these programs. The matrix *B* is used as a mask for the matrix of the decoder weights *W*, where the parameters for inactive genes in each GP are set to zero and do not change during training if the hard mask is used.

4 $$W_{i,j} = \left\{ {\begin{array}{*{20}{l}} {0\,{{{\mathrm{if}}}}\,B_{i,j} = 0,} \hfill \\ {w_{i,j}\,{{{\mathrm{otherwise}}}}} \hfill \end{array}} \right.$$

In the case of a soft mask, we add a regularization term that forces gene weights for genes that are not originally part of a GP to become zero, but also allows them to become active (non-zero) if they contribute to the reconstruction:

5 $$R_\gamma \left( W \right) = \gamma {\displaystyle{\mathop {\sum}\limits_j}} {\left\| {W_{:,\,j} \odot M_{:,\,j}} \right\|_1}$$

6 $$M_{i,j} = \left\{ {\begin{array}{*{20}{l}} {1\,{{{\mathrm{if}}}}\,B_{i,j} = 0,} \hfill \\ {0\,{{{\mathrm{otherwise}}}}} \hfill \end{array}} \right.$$

Some columns of M can be set to a vector of ones $M_{:,k} = \vec 1$ by setting $B_{:,k} = \vec 0$ to allow the introduction of sparse GPs.

Both variants (hard and soft masks) force the elements of *Z* to correspond to the GPs encoded in *W*.

### Learning new GPs

To allow new GPs to be learned, the model can be extended with additional nodes in reference training or query projection. For this, the last layer of the encoder is expanded with additional nodes connected to the existing nodes from the previous layer and producing the new vector **Z**_new_; in the decoder, the additional matrix *W*_new_ is concatenated to *W* (now denoted by *W*_old_), resulting in:

$$p\left( {\mathbf{X}|\mathbf{Z}_{{{{\mathrm{old}}}}},\mathbf {Z}_{{{{\mathrm{new}}}}},W_{{{{\mathrm{old}}}}},W_{{{{\mathrm{new}}}}},\mathbf{C}} \right)={{{\mathrm{NB}}}}\left( {g\left( {\left[ {\mathbf{Z}_{{{{\mathrm{old}}}}},\mathbf{ Z}_{{{{\mathrm{new}}}}},\mathbf{C}} \right]\left[ {W_{{{{\mathrm{old}}}}},W_{{{{\mathrm{new}}}}},L} \right]^T} \right),\mathbf CD} \right)$$

In addition, L1 regularization is added to *W*_new_, which is equivalent to the Laplace before this matrix. In addition, for each element of the vector **Z**_new_ the sample estimate of HSIC between the element and the other elements of **Z**_old_ and **Z**_new_ is added as a regularization term to the loss^22^. Also *W*_new_ can be constrained with hard gene membership or regularized with soft gene membership (see the previous section) as *W*_old_ using an additional GP database. In this case we do not use HSIC regularization for these new constrained nodes.

### Training

We use the stochastic proximal gradient descent to optimize the ELBO loss (equation (3)) with additional regularization terms. We also multiply the Kullback–Leibler (KL) divergence in the ELBO loss by the regularization coefficient *β*. Excluding the group lasso $R_\alpha \left( W \right) = - {{{\mathrm{log}}}}p\left( W \right)$ and soft mask term *R*_*γ*_(*W*) that appear in the proximal update step (discussed further), the loss function of the model can be written as:

7 $$\begin{array}{*{20}{l}} {F\left( {\theta ,\phi ,W} \right)} & = & {\frac{1}{N}{\displaystyle{\mathop {\sum}\limits_{i}^{N}}} {{\Bbb E}_{q_{\phi} \left( {\mathbf{ Z}_i|\mathbf{X}_i,\mathbf{C}} \right)}} \left[ { - {{{\mathrm{log}}}}p_{\theta} \left( {\mathbf{X}_i|\mathbf{Z}_i,W,\mathbf C} \right)} \right] + \beta {\Bbb K}{\Bbb L}\left( {q_{\phi} \left( {\mathbf{Z}_i|\mathbf{X}_i,\mathbf{C}} \right)\parallel p\left( {\mathbf{Z}_i} \right)} \right)} \hfill \\ {} \hfill & {} \hfill & { + \nu R_{\phi} ^{{{{\mathrm{HSIC}}}}}\left( {\mathbf{Z}_{{{{\mathrm{old}}}}},\mathbf{Z}_{{{{\mathrm{new}}}}}} \right)} \hfill \end{array}$$

where $R_{\phi} ^{{{{\mathrm{HSIC}}}}}\left( {\mathbf{Z}_{{{{\mathrm{old}}}}},\mathbf{ Z}_{{{{\mathrm{new}}}}}} \right)$ is a sample estimate of the HSIC regularization term. In addition, **Z**_new_ and **Z**_old_ are the old (existing in reference model) and new (learned in query training) unconstrained programs, respectively.

Then, to minimize the objective function we use the update scheme

8 $$\begin{array}{*{20}{l}} {\theta ^{\left( {t + 1} \right)}} \hfill & = \hfill & {\theta ^{\left( t \right)} - \eta \nabla _\theta {\hat {F}}\left( {\theta ,\phi ,W} \right)} \hfill \\ {\phi ^{\left( {t + 1} \right)}} \hfill & = \hfill & {\phi ^{\left( t \right)} - \eta \nabla _\phi {\hat {F}}\left( {\theta ,\phi ,W} \right)} \hfill \\ {W^{\left( {t + 1} \right)}} \hfill & = \hfill & {\begin{array}{*{20}{c}} {{{{\mathrm{Prox}}}}} \\ {\eta R_\alpha + \eta R_\gamma } \end{array}\left( {W^{\left( t \right)} - \eta \nabla _W{\hat {F}}\left( {\theta ,\phi ,W} \right)} \right)} \hfill \end{array}$$

where ${\hat {F}}\left( {\theta ,\phi ,W} \right)$ denotes an estimate of the function (equation (7)) over a mini-batch of samples (as in the standard stochastic gradient descent algorithm), *t* is the step in the gradient descent algorithm and *η* is the learning rate.

$$\left( {R_\alpha + R_\gamma } \right)\left( W\, \right) = \alpha{\displaystyle{ \mathop {\sum}\limits_j}} {\left\| {W_{:,\,j}} \right\|_2} + \gamma {\displaystyle{\mathop {\sum}\limits_j}} {\left\| {W_{:,\,j} \odot M_{:,\,j}} \right\|_1}$$

is the lasso and soft mask regularization term and its proximal operator is

9 $$\begin{array}{*{20}{c}} {{{{\mathrm{Prox}}}}} \\ {\eta R_\alpha + \eta R_\gamma } \end{array}\left( V\, \right) = {{{\mathrm{arg}}\,{\mathrm{min}}}}_L\left[ {\frac{1}{2}\Vert {L - V}{\Vert}_F^2 + \eta \alpha {\displaystyle{\mathop{\sum}\limits_{j}}} {\Vert {L_{:,\,j}} {\Vert}_2} + \eta \gamma {\displaystyle{\mathop {\sum}\limits_{j}}} {\Vert {L_{:,\,j} \odot M_{:,\,j}} {\Vert}_1} } \right]$$

The hard mask variant implies *γ* = 0. The proximal operator above has a closed-form expression (see the next section for the derivation), so it is easy to apply it after the stochastic gradient descent update. The gradient for the expectation terms is obtained with the reparametrization trick, as is common in the VAE framework^96^.

### Proximal operators for expiMap

To derive the closed form of the proximal operator described in the previous section, we need two theorems.

#### Theorem 1

(Proximal operator of separable functions.)

Suppose that $f:E_1 \times E_2 \times \ldots \times E_m \to ( - \infty ,\infty ]$ is given by

$$\begin{array}{*{20}{l}} {f\left( {\mathbf{x}_1,\mathbf{x}_2, \ldots ,\mathbf{x}_m} \right) ={\displaystyle{ \mathop {\sum}\limits_{i = 1}^m}}\, {f_i\left( {\mathbf{x}_i} \right)} } \hfill \\ {\mathbf{x}_i \in E_i,i = 1,2, \ldots ,m} \hfill \end{array}$$

Then for any $x_1 \in E_1,x_2 \in E_2, \ldots ,x_m \in E_m$,

$$\begin{array}{*{20}{c}} {{{{\mathrm{Prox}}}}} \\ f \end{array}\left( {\mathbf{x}_1,\mathbf{x}_2, \ldots ,\mathbf{x}_m} \right) = \begin{array}{*{20}{c}} {{{{\mathrm{Prox}}}}} \\ {f_1} \end{array}\left( {\mathbf{x}_1} \right) \times \begin{array}{*{20}{c}} {{{{\mathrm{Prox}}}}} \\ {f_2} \end{array}\left( {\mathbf{x}_2} \right) \times \ldots \times \begin{array}{*{20}{c}} {{{{\mathrm{Prox}}}}} \\ {f_m} \end{array}\left( {\mathbf{x}_m} \right)$$

where *E*_*i*_ denotes a vector space and × is a Cartesian product. The proof of this theorem can be found in ref. ^98^.

#### Theorem 2

(Decomposition of the proximal operator.)

A sufficient condition for $$\begin{array}{*{20}{c}} {{{{\mathrm{Prox}}}}} \\ {f + g} \end{array} = \begin{array}{*{20}{c}} {{{{\mathrm{Prox}}}}} \\ f \end{array} \circ \begin{array}{*{20}{c}} {{{{\mathrm{Prox}}}}} \\ g \end{array}$$ is

$$\forall \mathbf{x} \in H\,\partial g\left( {\begin{array}{*{20}{c}} {{{{\mathrm{Prox}}}}} \\ f \end{array}\left( \mathbf{x} \right)} \right) \supseteq \partial g\left( \mathbf{x} \right)$$

where *f* and *g* are closed (or, equivalently here, continuous), convex functions; *H* denotes a Hilbert space; and $\partial g$ stands for a subgradient of *g*. The proof of the theorem can be found in ref. ^99^.

We use the two theorems above to find the closed form of the proximal operator (equation (9)). The explicit form of the regularization function is:

10 $$R_{\alpha ,\gamma }\left( W \,\right) = \left( {R_\alpha + R_\gamma } \right)\left( W \right) = \alpha{\displaystyle{ \mathop {\sum}\limits_j}} {\left\| {W_{:,\,j}} \right\|_2} + \gamma{\displaystyle{ \mathop {\sum}\limits_j}} {\left\| {W_{:,\,j} \odot M_{:,\,j}} \right\|_1}$$

The sums in the regularization function are made over columns of *W*; thus, this function is clearly separable in columns, and the theorem 1 is applicable here. This means that we only need to calculate the proximal operator for a column, as we can find the full proximal operator as a Cartesian product of the proximal operators for different columns. This is the same as using its own proximal operator for each column of *W* separately.

The regularization summand for a separate column *k* of *W* can be written as

11 $$R_{\alpha ,\gamma }^k\left( W\, \right) = \left( {R_\alpha ^k + R_\gamma ^k} \right)\left( W\, \right) = \alpha \left\| {W_{:,\,k}} \right\|_2 + \gamma \left\| {W_{:,\,k} \odot M_{:,k}} \right\|_1$$

The regularization summand (equation (11)) has the form of a sum, so the theorem 2 has to be used.

For the group lasso part $\alpha \parallel \cdot \parallel _2$ the proximal operator can be immediately obtained (from ref. ^98^) as

12 $$\begin{array}{*{20}{c}} {{{{\mathrm{Prox}}}}} \\ {\eta R_\alpha ^k} \end{array}\left( \mathbf{v} \right) = \left\{ {\begin{array}{*{20}{l}} {\mathbf{v} - \eta \alpha \frac{\mathbf{v}}{{\left\| \mathbf{v} \right\|_2}},} \hfill & {{{{\mathrm{if}}}}\,\left\| \mathbf{v} \right\|_2 > \eta \alpha } \hfill \\ {0,} \hfill & {{{{\mathrm{if}}}}\,\left\| \mathbf{v} \right\|_2 \le \eta \alpha } \hfill \end{array}} \right.$$

It should be noted that in the case when the mask’s column equals a vector of ones $M_{:,\,k} = \vec 1$ the proximal operator for the second summand in equation (11) $\gamma \parallel \cdot \parallel _1$ is just a proximal operator for a standard L1 regularization and can be written^98^ as

13 $$\begin{array}{*{20}{l}} {{{{\mathcal{T}}}}_\gamma \left( y \right)} = {\left\{ {\begin{array}{*{20}{l}} {y - \gamma ,} \hfill & {{{{\mathrm{if}}}}\,y \ge \gamma } \hfill \\ {0,} \hfill & {{{{\mathrm{if}}}}\,\left| y \right| < \gamma } \hfill \\ {y - \gamma ,} \hfill & {{{{\mathrm{if}}}}\,y \le \gamma } \hfill \end{array}} \right.} \hfill \\ {\begin{array}{*{20}{c}} {{{{\mathrm{Prox}}}}} \\ {\gamma \parallel \cdot \parallel _1} \end{array}\left( \mathbf{v} \right)} = {{{{\mathcal{T}}}}_\gamma \left( {v_1} \right) \times {{{\mathcal{T}}}}_\gamma \left( {v_2} \right) \times \ldots \times {{{\mathcal{T}}}}_\gamma \left( {v_G} \right)} \hfill \end{array}$$

In addition, the subgradient $\partial \left( {\gamma \parallel \mathbf{v}\parallel _1} \right)$ (from ref. ^98^, rewritten) is

14 $$\begin{array}{*{20}{l}} {{{{\mathrm{sgn}}}}_\gamma \left( y \right)} \hfill & = \hfill & {\left\{ {\begin{array}{*{20}{l}} {\gamma ,} \hfill & {{{{\mathrm{if}}}}\,y > 0} \hfill \\ {\left[ { - \gamma ,\gamma } \right],} \hfill & {{{{\mathrm{if}}}}\,y = 0,} \hfill \\ { - \gamma ,} \hfill & {{{{\mathrm{if}}}}\,y < 0} \hfill \end{array}} \right.} \hfill \\ {\partial \left( {\gamma \parallel \mathbf{v}\parallel _1} \right)} \hfill & = \hfill & {{{{\mathrm{sgn}}}}_\gamma \left( {v_1} \right) \times {{{\mathrm{sgn}}}}_\gamma \left( {v_2} \right) \times \ldots \times {{{\mathrm{sgn}}}}_\gamma \left( {v_G} \right)} \hfill \end{array}$$

The proximal operator $$\begin{array}{*{20}{c}} {{{{\mathrm{Prox}}}}} \\ {\alpha \parallel \cdot \parallel _2} \end{array}\left( \mathbf{v} \right)$$ is equal to equation (12) (without *η*). By direct calculation for $$\mathbf{v}^ \ast = \begin{array}{*{20}{c}} {{{{\mathrm{Prox}}}}} \\ {\alpha \parallel \cdot \parallel _2} \end{array}\left( \mathbf{v} \right)$$ the following holds $\forall i = 1, \ldots ,G$: if $v_i = 0$, then $v_i^ \ast = 0$; if $v_i < 0$, then $v_i^ \ast \le 0$; if $v_i > 0$, then $v_i^ \ast \ge 0$.

This basically means that ${{{\mathrm{sgn}}}}_\gamma \left( {v_i^ \ast } \right) \supseteq {{{\mathrm{sgn}}}}_\gamma \left( {v_i} \right)$. It immediately follows from the form of the subgradient (equation (14)) that

$$\begin{array}{*{20}{l}} {\mathbf{v}^ \ast = \begin{array}{*{20}{c}} {{{{\mathrm{Prox}}}}} \\ {\alpha \parallel \cdot \parallel _2} \end{array}\left( \mathbf{v} \right)} \hfill \\ {\partial \left( {\gamma \parallel \mathbf{v}^ \ast \parallel _1} \right) \supseteq \partial \left( {\gamma \parallel \mathbf{v}\parallel _1} \right)} \hfill \end{array}$$

Using this and the theorem 2 we can conclude that

15 $$\begin{array}{*{20}{c}} {{{{\mathrm{Prox}}}}} \\ {\gamma \parallel \cdot \parallel _1 + \alpha \parallel \cdot \parallel _2} \end{array}\left( \mathbf{v} \right) = \begin{array}{*{20}{c}} {{{{\mathrm{Prox}}}}} \\ {\alpha \parallel \cdot \parallel _2} \end{array}\left( {\begin{array}{*{20}{c}} {{{{\mathrm{Prox}}}}} \\ {\gamma \parallel \cdot \parallel _1} \end{array}\left( \mathbf{v} \right)} \right)$$

Therefore, the closed form of the proximal operator for the case $M_{:,\,k} = \vec 1$ is:

16 $$\begin{array}{*{20}{c}} {{{{\mathrm{Prox}}}}} \\ {\eta R_{\alpha ,\gamma }^k} \end{array}\left( \mathbf{v} \right) = \begin{array}{*{20}{c}} {{{{\mathrm{Prox}}}}} \\ {\eta \alpha \parallel \cdot \parallel _2} \end{array}\left( {\begin{array}{*{20}{c}} {{{{\mathrm{Prox}}}}} \\ {\eta \gamma \parallel \cdot \parallel _1} \end{array}\left( \mathbf{v} \right)} \right)$$

Moreover, the closed forms of $$\begin{array}{*{20}{c}} {{{{\mathrm{Prox}}}}} \\ {\eta \alpha \parallel \cdot \parallel _2} \end{array}\left( \mathbf{v} \right)$$ and $$\begin{array}{*{20}{c}} {{{{\mathrm{Prox}}}}} \\ {\eta \gamma \parallel \cdot \parallel _1} \end{array}\left( \mathbf{v} \,\right)$$ are given in equations (12) and (13), respectively.

For the case $M_{:,\,k} \ne \vec 1$, similar reasoning can be applied. First, the closed form of the proximal operator $$\begin{array}{*{20}{c}} {{{{\mathrm{Prox}}}}} \\ {\gamma \parallel \, \cdot \, \odot \,M_{:,\,k}\parallel _1} \end{array}\left( \mathbf{v} \right)$$ for L1 norm of the vector of genes (gene weights in the factor) that are inactive in the annotation for the factor *k* can be written as

17 $$\begin{array}{l} {{{{\mathcal{A}}}}_\gamma ^{g,k}\left( y \right)} ={\left\{ {\begin{array}{lll} {y,}&{{{{\mathrm{if}}}}\,M_{g,k} = 0}\\ {{{{\mathcal{T}}}}_\gamma \left( y \right),}&{{{{\mathrm{if}}}}\,M_{g,k} = 1}\end{array}} \right.} \hfill \\ {\begin{array}{*{20}{c}} {{{{\mathrm{Prox}}}}} \\ {\gamma \parallel \cdot \odot M_{:,\,k}\parallel _1} \end{array}\left( \mathbf{v} \right)}= {{{{\mathcal{A}}}}_\gamma ^{1,k}\left( {v_1} \right) \times \ldots \times {{{\mathcal{A}}}}_\gamma ^{G,k}\left( {v_G} \right)} \hfill \end{array}$$

where ${{{\mathcal{T}}}}_\gamma \left( y \right)$ is the same as in equation (13).

The subgradient $\partial \left( {\gamma \parallel \,\mathbf{v}\, \odot \,M_{:,\,k}\parallel _1} \right)$ can be written as

18 $$\begin{array}{l} {{{{\mathcal{S}}}}_\gamma ^{g,k}\left( y \right)}={\left\{ {\begin{array}{*{20}{l}} {0,} \hfill & {{{{\mathrm{if}}}}\,M_{g,k} = 0} \hfill \\ {{{{\mathrm{sgn}}}}_\gamma \left( y \right),} \hfill & {{{{\mathrm{if}}}}\,M_{g,k} = 1} \hfill \end{array}} \right.} \hfill \\ {\partial \left( {\gamma \parallel \mathbf{v} \odot M_{:,\,k}\parallel _1} \right)}={{{{\mathcal{S}}}}_\gamma ^{1,k}\left( {v_1} \right) \times \ldots \times {{{\mathcal{S}}}}_{\lambda _1}^{G,k}\left( {v_G} \right)} \hfill \end{array}$$

where ${{{\mathrm{sgn}}}}_\gamma \left( y \right)$ is the same as in equation (14).

Using the same reasoning as in the derivation of the proximal operator for sparse unannotated factors, we see that

$$\begin{array}{*{20}{l}} {\mathbf{v}^ \ast = \begin{array}{*{20}{c}} {{{{\mathrm{Prox}}}}} \\ {\alpha \parallel \cdot \parallel _2} \end{array}\left( \mathbf{v} \right)} \hfill \\ {\partial \left( {\gamma \parallel \mathbf{v}^ \ast \, \odot \,M_{:,\,k}\parallel _1} \right) \supseteq \partial \left( {\gamma \parallel \mathbf{v}\, \odot \,M_{:,\,k}\parallel _1} \right)} \hfill \end{array}$$

This means that we again can use the theorem 2 and obtain the closed form of the proximal operator (with the learning rate *η*) for the column *k* of *W*, which corresponds to the annotated factor *k*

19 $$\begin{array}{*{20}{c}} {{{{\mathrm{Prox}}}}} \\ {\eta R_{\alpha ,\gamma }^k} \end{array}\left( \mathbf{v} \right) = \begin{array}{*{20}{c}} {{{{\mathrm{Prox}}}}} \\ {\eta \alpha \parallel \cdot \parallel _2} \end{array}\left( {\begin{array}{*{20}{c}} {{{{\mathrm{Prox}}}}} \\ {\eta \gamma \parallel \cdot \, \odot \,M_{:,\,k}\parallel _1} \end{array}\left( \mathbf{v} \right)} \right)$$

In addition, the closed forms of $$\begin{array}{*{20}{c}} {{{{\mathrm{Prox}}}}} \\ {\eta \alpha \parallel \cdot \parallel _2} \end{array}\left( \mathbf{v} \right)$$ and $$\begin{array}{*{20}{c}} {{{{\mathrm{Prox}}}}} \\ {\eta \gamma \parallel \, \cdot \, \odot \,M_{:,\,k}\parallel _1} \end{array}\left( \mathbf{v} \right)$$ are given in equations (12) and (17), respectively.

Theorem 1 allows calculation of the output of the joint proximal operator $$\begin{array}{*{20}{c}} {{{{\mathrm{Prox}}}}} \\ {\eta R_\alpha + \eta R_\gamma } \end{array}\left( \cdot \right)$$ in equation (8) by applying the proximal operators (equations (12), (16) or (19)) on each column of the input of the joint operator independently.

### Reference mapping

The projection of a query dataset to a reference dataset is performed using the single-cell architectural surgery (scArches) approach^15^. After training a conditional VAE model for multiple batches of the reference dataset, the trained weights are transferred to a new model with additional conditional nodes used to map new query batches to the reference. Further, additional nodes for new learnable GPs can be added at this stage (see the learning new GP section). During the training of the expanded model for query projection, only the conditional weights connecting new batches and the weights for new GPs (if any) in both encoder and decoder are tuned; the rest of the weights are frozen. Projecting with scArches preserves the latent representation of the reference and projects the query data to the same latent space while correcting for batch effects between the query and data.

### Differential testing for GPs

To test the hypothesis $H_0:Z_{i,a} > Z_{i,b}$ versus $H_1:Z_{i,a} \le Z_{i,b}$, where $Z_{i,a},Z_{i,b}$ are the dimension *i* of the latent variables for the cells from the groups *a* and *b* respectively, we use the logarithm of the Bayes factor:

20 $${{{\mathrm{log}}}}K = {{{\mathrm{log}}}}\frac{{p\left( {H_0} \right)}}{{p\left( {H_1} \right)}} = {{{\mathrm{log}}}}\frac{{p\left( {H_0} \right)}}{{1 - p\left( {H_0} \right)}}$$

where $p\left( {H_0} \right)$ and $p\left( {H_1} \right)$ are the probabilities of the hypotheses *H*_0_ and *H*_1_, respectively.

We can compute $P\left( {H_0} \right)$ as:

21 $$\begin{array}{l}p\left( {H_0} \right) = p\left( {Z_{1,i} > Z_{2,i}|G_1 = a,G_2 = b} \right)\\\qquad\quad = {\Bbb E}_{p\left( {\mathbf X_1,\mathbf C_1|G_1 = a} \right)p\left( {\mathbf X_2,\mathbf C_2|G_2 = b} \right)}\left[ {p\left( {Z_{1,i} > Z_{2,i}|\mathbf X_1,\mathbf X_2,\mathbf C_1,\mathbf C_2} \right)} \right]\end{array}$$

where *G*_1_ and *G*_2_ denote the independent group variables for **X**_1_ and **X**_2_, respectively.

The probability $p\left( {Z_{1,i} > Z_{2,i}|\mathbf X_1,\mathbf X_2,\mathbf C_1,\mathbf C_2} \right)$ inside the expectation in equation (21) can be estimated with the approximate posteriors $q_\phi \left( {Z_{1,i}|\mathbf X,\mathbf C} \right)$ and $q_\phi \left( {Z_{2,i}|\mathbf X,\mathbf C} \right)$, as follows:

22 $$p\left( {Z_{1,i} > Z_{2,i}|\mathbf X_1,\mathbf X_2,\mathbf C_1,\mathbf C_2} \right) \approx {\Bbb E}_{q_\phi \left( {Z_{1,i}|\mathbf X_1,\mathbf C_1} \right)q_\phi \left( {Z_{2,i}|\mathbf X_2,\mathbf C_2} \right)}\left[ {I\left( {Z_{1,i} > Z_{2,i}} \right)} \right]$$

where the expectation could be approximated by sampling or calculated from the closed form. When $q_\phi \left( {Z_i|\mathbf X,\mathbf C} \right)$ is Gaussian, we can calculate the expectation by

23 $$\begin{array}{l}{\Bbb E}_{q_\phi \left( {Z_{1,i}|\mathbf X_1,\mathbf C_1} \right)q_\phi \left( {Z_{2,i}|\mathbf X_2,\mathbf C_2} \right)}\left[ {I\left( {Z_{1,i} > Z_{2,i}} \right)} \right] = \frac{1}{2}{{{\mathrm{erfc}}}}\left( { - \frac{{\mu _i\left( {\mathbf X_1,\mathbf C_1} \right) - \mu _i\left( {\mathbf X_2,\mathbf C_2} \right)}}{{\sqrt {2\left( {\sigma _i^2\left( {\mathbf X_1,\mathbf C_1} \right) + \sigma _i^2\left( {\mathbf X_2,\mathbf C_2} \right)} \right)} }}} \right)\end{array}$$

The probabilities (equation (22)) can be averaged over many cells from both groups as in scVI^40^, to obtain the approximate value for equation (21).

Through the examples in the paper, we refer to the results obtained as ‘expiMap test results’ and at the threshold $\left| {{{{\mathrm{log}}}}K} \right| \ge 2.3$ as the ‘enriched results’, and call such GPs ‘differential GPs’ in the comparison of interest in this work.

### Gene importance score

Gene importance score for a gene in a GP is the absolute value of the decoder weight for the gene in the GP. Each column of the weight matrix *W* in the decoder (2) corresponds to a GP and each row corresponds to a gene. Because of the linearity of the decoder, a change in the latent score of the *i*th GP *Z*_*i*_ affects the reconstruction of gene counts more for those genes with higher absolute values of the weights in *W*_:,*i*_. Consequently, we can rank genes in each GP by the absolute values of their weights in *W*. This ranking reflects the relative importance of a given GP for each gene; a higher ranking means that this gene is affected more by the GP.

### Latent scores directions

The signs of latent scores of GPs do not necessary correspond to up- or downregulation of these GPs. However, in some cases, it is possible to determine whether an increase in a latent score corresponds primarily to an increase or decrease in the expression of genes of a corresponding GP. This can be determined by analysing the decoder gene weights in the column corresponding to the GP (as described in the previous section). If most of the gene weights in the column of *W* corresponding to the GP are positive, then the higher positive latent score implies upregulation; in the opposite case of mostly negative weights, a lower negative score also means upregulation.

For the *j*th GP, the direction *D*_*j*_ of predominant upregulation (negative or positive) can be calculated heuristically by several methods. We use two methods:

$$\begin{array}{*{20}{l}} {{{{\mathrm{sum:}}}}D_j} \hfill & = \hfill & {{{{\mathrm{sign}}}}\left({\displaystyle{ {\mathop {\sum }\limits_i W_{i,j}}}} \right)} \hfill \\ {{{{\mathrm{counts:}}}}D_j} \hfill & = \hfill & {{{{\mathrm{sign}}}}\left({\displaystyle{ {\mathop {\sum }\limits_i }}{{{\mathrm{sign}}}}\left( {W_{i,j}} \right)} \right)} \hfill \end{array}$$

Then, we can multiply the latent score of the GP by this direction $\tilde Z_j = Z_j \times D_j$, so that a higher positive value of $\tilde Z_j$ always corresponds to predominant upregulation of the GP and a lower negative value to downregulation. These normalized scores can then be used for plotting or testing.

### Metrics for integration and evaluation

Integrations were evaluated with methods implemented in scIB. We evaluated biological conservation through graph cLISI, normalized mutual information (NMI), adjusted Rand index (ARI) and average silhouette width (ASW) for cell type; and batch correction through principal component regression, ASW for batch, kBET, graph connectivity and graph iLISI. All metrics are further described in the scIB paper^60^. The overall score was computed as the average of all scores.

We assessed the dominance of genes in a GP for Extended Data Fig. 3e by normalized entropy. The normalized entropy is calculated by dividing the entropy of the distribution of gene importance scores of the GP by the entropy of the uniform discrete distribution of the same size. The distribution of gene importance scores is obtained by dividing each score by the total sum of the scores. The normalized entropy scale is from 0 (absolutely concentrated) to 1 (uniformly spread weights).

### Choice of hyperparameters for expiMap training

#### Reference training and integration

The main hyperparameter that affects the quality of integration for the reference training is **alpha_kl**, the value of which is multiplied by the kl divergence term in the total loss. If the visualized latent space looks like a single blob after the reference training, we recommend to decrease the value of **alpha_kl**. If the visualized latent space shows bad integration quality, we recommend to increase the value of **alpha_kl**. The good default value in most cases is **alpha_kl** = **0.5**. The required strength of group lasso regularization (**alpha**) depends on the number of used GPs and the size of the dataset. For 300–500 GPs, we recommend to use **alpha** = **0.7** and increase for larger numbers of GPs.

#### Reference mapping

We recommend to use 200 epochs and **early_stopping** = **True** for the query to reference mapping. Smaller datasets tend to require more epochs of training to map the query into the reference well. If you observe that the query is not integrated into the reference, we recommend to try longer training for the query mapping.

When using new unconstrained GPs, we recommend to start with ten of them or more. This ensures that all new significant sources of variation in the query would be covered by the new GPs. We also recommend to use L1 regularization for the new GPs (the **gamma_ext** parameter), it will make them sparser, and thus more interpretable, and also can de-activate redundant new GPs completely, which is important when the number of new unconstrained GPs is high.

If you use new constrained GPs with soft masks, it is important to monitor share of de-activated inactive genes of the soft masks in the constrained GPs. Set **print_stats** = **True** during the training, and check that at the end of the training process ‘Share of de-activated inactive genes in extension terms’ log show a number higher than 0.9. If this number is lower, it means that some of the constrained GPs lost their specialization given by the soft mask and added a lot of irrelevant genes. If this happens, it is better to increase the **alpha_l1** parameter and retrain the model.

### Non-amortized scVI

We compared the integration performance of expiMap with scVI and non-amortized scVI. Non-amortized scVI is a VAE model similar to scVI, where the neural network encoder was replaced by a per cell vector of parameters for each cell in a dataset.

For each cell *i* there are vectors $\mathbf \mu _i \in {\Bbb R}^Z$ and $\mathbf \sigma _i^2 \in {\Bbb R}^Z$ with the size of the latent space. The *j*th latent variable for the cell *i* is obtained by sampling independently from the Gaussian distribution $Z_{i,j} \sim {{{\mathcal{N}}}}\left( {\mu _{i,\,j},\sigma _{i,\,j}^2} \right)$. The decoder is the same as in the standard scVI model. The non-amortized scVI model is trained by minimizing the negative ELBO in batches with a gradient descent algorithm as a standard VAE model.

### GSEA using limma-fry

Read counts were normalized using the trimmed mean of M-values (TMM)^100^ with singleton pairing implemented in edgeR^101^ to account for sparsity in the single cell RNAseq data. The fry test (Fast Approximation to ROAST)^59^ in limma^58^ R/Bioconductor package was applied to log counts per million (logCPM) values obtained by voom transformation^102^ to test for the enrichment of the gene set terms in the Reactome pathway database^78^. The Reactome database was obtained from the Molecular Signature Database (MSigDB)^103,104^.

### Datasets and pre-processing

All the cell type labels and metadata were obtained from original publications unless specifically stated below.

#### Immune healthy atlas

The immune dataset includes samples from bone marrow cells and peripheral blood cells from different human samples. The bone marrow data were collected from Oetjen et al.^48^ and PBMC samples were obtained from 10x Genomics (https://support.10xgenomics.com/single-cell-gene-expression/datasets/3.0.0/pbmc_10k_v3), Freytag et al.^49^ and Sun et al.^50^. The detail of the retrieval path and the pre-processing can be found in Luecken et al.^60^ and Lotfollahi et al.^15^ We used the Reactome pathway database for annotations^78^ from MsigDB^103,104^; we also removed all pathways with fewer than 12 genes. The genes that were not in the GPs database were filtered out, reducing the total number of genes from approximately 11,000 to 3,690. Then 2,000 highly variable genes were selected for training.

#### PBMC IFN-β

This dataset contains cells from eight patients with Lupus treated with IFN-β or left untreated for 6 h (ref. ^105^). The pools from the IFN-β and control cells were mixed together and loaded to a 10x kit. The dataset was obtained from the Seurat tutorial (https://satijalab.org/seurat/articles/integration_introduction.html). We have used the same genes as in the reference (Immune atlas).

#### PBMC COVID-19

The dataset^62^ contains five peripheral blood samples from two patients with severe COVID-19 at three different timepoints, consisting of severe remission during treatment with tocilizumab. The blood samples were collected on day 1, within 12 h of tocilizumab treatment, and on day 5 for both patients. An additional blood sample was collected from patient 2 because the patient remained COVID-19 positive. The cell types were annotated using markers provided by the authors in the original study. We have used the same genes as in the reference (Immune atlas). The dataset is available on Gene Expression Omnibus (GEO); the accession number is GSE150861.

We used the integrated dataset to analyse cell–cell interactions using the CellChat package^66^. For this analysis, we used the non-integrated shared gene space between all the integrated datasets after removing those genes supported by fewer than five counts, for a total of 10,851 genes ready for analysis. We then ran CellChat on each subset using the curated database for interactions in human samples. The gene expression of each ligand–receptor pair was visualized using a dotplot generated using Scanpy 1.8.1 (ref. ^61^) and anndata 0.7.6 (ref. ^106^). The scripts for the analyses, as well as the package version used in the analysis, can be found in the ‘covid’ section of the repository.

#### Pancreas

The datasets are publicly available on GEO and further described in Supplementary Table 15. We removed low-quality cells (high mitochondrial fraction and low number of genes) using a study-specific threshold. For cell type annotation, we removed genes expressed in fewer than 20 cells in each study and normalized the expression in each study to 1 × 10^6^ total counts, excluding highly expressed genes, and subsequently applied a log transformation. We merged datasets across studies using Ensembl IDs and retaining the genes expressed in all studies. We used merged data across studies, followed by the identification of highly variable genes, *z*-normalization and the computation of top PCA components. We clustered the data and plotted known pancreatic islet cell type markers to annotate cell types cluster-wise.

For integration, we separated the datasets into reference and query, as described in Supplementary Table 15. From the reference data, we removed immune cell types and their doublets. We removed genes expressed in fewer than 20 cells in the reference data. We used gene sets from PanglaoDB^107^ release from March 2020 and Reactome^78^ v4.0 and mapped them to mouse genes using Ensembl^108^ V103 orthologues. We used only gene sets with at least three genes and at most 200 genes. We excluded genes that were not present in these gene sets. With expiMap, we integrated the reference datasets using samples as batches and projected query samples. We also performed matched integrations with Seurat^109^, Symphony^18^ and scVI^40^. We evaluated different integrations, as described in the integration evaluation section. We used reference query split as batches and excluded non-healthy query samples as they were not expected to be integrated into the healthy reference owning to biological differences. For the downstream interpretation analysis, we used directed expiMap scores.

We used multiple methods to evaluate PanglaoDB cell type scores. We plotted the PanglaoDB cell type scores of expected pancreatic cell types on query UMAPs and visually compared the results to cell type annotation. We used the PanglaoDB gene set scores as features for the annotation transfer from reference to query with weighted *k*-nearest neighbour (kNN), as described in ref. ^15^. We evaluated the annotation transfer with F1 score and by visual evaluation of prediction accuracy and certainty on UMAP.

For gene-level analyses on integrated data we normalized expression with Scanpy using functions normalize_total and log1p.

#### Integration benchmark datasets

We leverage datasets from five different tissues including PBMCs (*n* = 161,764) (ref. ^109^), heart (*n* = 18,641) (ref. ^11^), lung (*n* = 65,662) (ref. ^13^), colon (*n* = 34,772) (ref. ^110^) and liver (*n* = 113,063) (ref. ^111^). All datasets, except heart, were obtained from the Sfaira database^112^, which includes cell type labels. Heart was obtained from the scVI package. For the expiMap training for each dataset, we used the Reactome pathway database, selected only pathways that contain more than 12 genes and filtered out all genes that are not present in any pathway, and then we selected 2,000 HVG for training. For the other models, we used the same lists of genes.

#### Mouse endocrinogenesis

We used the developmental dataset from mouse endocrinogenesis (*n* = 25,919) (ref. ^86^). The raw dataset is available at the GEO under accession number GSE132188. Cell type labels were obtained from an adata object provided by the authors of scVelo^113^. We used the Reactome pathway database version 7.5.1 for annotations^78^ from MsigDB^103,104^. Days 14.5 and 15.5 were used as a reference, and days 12.5 and 13.5 as a query. For the reference dataset, we removed all pathways with fewer than 13 genes. The genes that were not in the GPs database were filtered out, reducing the total number of genes from approximately 28,000 to 10,000. Then 4,000 highly variable genes were selected for training. For the query, we used the genes obtained after pre-processing the reference dataset. RNA velocities were calculated using scVelo.

### Methods for the query to reference benchmarks

scVI: we used the setup from the scarches tutorial for query to reference mapping with scVI (https://scarches.readthedocs.io/en/latest/scvi_surgery_pipeline.html).

Symphony: we used the parameters recommended in the repository (https://github.com/immunogenomics/symphony).

Seurat: we adapted the Seurat reference mapping tutorial (https://satijalab.org/seurat/articles/integration_mapping.html), but used supervised principal component analysis(sPCA) instead of PCA.

### Statistics and reproducibility

The details for pre-processing of the datasets used for the model training are provided in the section ‘Datasets and pre-processing’. If not indicated otherwise in that section or in the legends, no data were excluded from training and analysis. The hyperparameters chosen for model training for all experiments are listed in Supplementary Note 12: hyperparameters. The details of statistical tests employed for differential testing of GPs are provided in the sections ‘Differential testing for GPs’ and Supplementary Note 1: comparison with limma-fry. Metrics for integration used in the paper are described in the section ‘Metrics for integration and evaluation’. Robustness of query to reference mapping for different query dataset sizes is analysed in Supplementary Note 3: robustness of the model under different data query dataset sizes. Reproducibility and robustness of newly learned GPs are discussed in Supplementary Note 6: disentanglement and robustness of newly learned GPs.

### Protocol

A step-by-step protocol for installing the software, training the model and downstream analysis can be found on Nature Protocol Exchange^114^.

### Reporting summary

Further information on research design is available in the Nature Portfolio Reporting Summary linked to this article.

## Online content

Any methods, additional references, Nature Portfolio reporting summaries, source data, extended data, supplementary information, acknowledgements, peer review information; details of author contributions and competing interests; and statements of data and code availability are available at 10.1038/s41556-022-01072-x.

## Supplementary information

Supplementary Information Supplementary Notes 1–12 and Figs. 1–10.

Reporting Summary

Supplementary Tables Supplementary Tables 1–15. Each tab is a Supplementary table, and descriptions and captions are provided in the tabs.

Supplementary Data 1 expiMap latent scores.

Supplementary Data 2 expiMap latent scores for cells of each cell type listed in the figure; Bayes factors for IFN-β stimulated versus rest for each cell type in the figure.

Supplementary Data 3 expiMap latent scores for cells of each cell type listed in the figure; Bayes factors for IFN-β stimulated versus rest for each cell type in the figure.

Supplementary Data 4 UMAP coordinates for plots in the figure.

Supplementary Data 5 UMAP coordinates of the full model and the model with removed top IFN-β GP with condition, study and cell type labels.

Supplementary Data 6 Results from CellChat.

Supplementary Data 7 UMAP coordinates of expiMap latent space, annotations and annotation transfer scores and uncertainty.

Supplementary Data 8 UMAP coordinates of the expiMap latent space, cell type markers and expiMap latent scores for cell types.

Supplementary Data 9 expiMap latent scores of GPs listed in the figure.

Supplementary Data 10 Bayes factors for testing each cell type versus rest.

## Data Availability

The Immune healthy atlas, PBMC IFN-β, PBMC COVID-19, mouse endocrinogenesis datasets and the heart dataset used for the integration benchmark are public, referenced and downloadable at https://github.com/theislab/expiMap_reproducibility. The Pancreas datasets are publicly available and can be accessed with the following GEO codes: STZ (GSE128565), Fltp_P16 (GSE161966), NOD (GSE144471), spikein_drug (GSE147203/GSE142465 (GSM4228185–GSM4228199)) and NOD_elimination (GSE117770). The PBMCs, lung and colon liver datasets used in the integration benchmark are public, referenced and can be obtained from the sfaira database^112^ (https://theislab.github.io/sfaira-portal/). The data supporting the findings of this study can be reproduced using codes and notebooks available at https://github.com/theislab/expiMap_reproducibility. All other data supporting the findings of this study are available from the corresponding author on reasonable request. Source data are provided with this paper.

## Source data

Source Data Fig. 2 UMAP coordinates of the latent space for Fig. 2a, Bayes factors for the test of IFN-β-stimulated cells versus control for Fig. 2b and expiMap latent scores for GPs in Fig. 2c–e.
Source Data Fig. 3 UMAP coordinates of the latent space for Fig. 3a, values of control integration metrics in Fig. 2b and reference building metrics values for Fig. 2c.
Source Data Fig. 4 expiMap latent scores for new GPs for Fig. 4a,d–f, B-cell node decoder weights for Fig. 4b, node 1 and node 2 decoder weights for Fig. 4c and UMAP coordinates of the latent space and log-normalized expression of TMSB4X for Fig. 4g–i.
Source Data Fig. 5 UMAP coordinates for Fig. 5b,c, expiMap latent scores for Fig. 5c,d and results from CellChat for Fig. 5f.
Source Data Fig. 6 UMAP coordinates of the latent space for Fig. 6b–d, membership matrix of genes for GPs/terms (0 means that a gene is not present in a GP, 1 means gene is present) for Fig. 6e and expiMap latent scores for Fig. 6f–h.
Source Data Extended Data Fig. 1 Bayes factors for differential GP analysis between cell types (one versus all test) for cell types in the query data.
Source Data Extended Data Fig. 2 Bayes factors from expiMap and false discovery rate from fry.
Source Data Extended Data Fig. 3 Pearson correlation coefficient values between mean log-normalized expression and decoder weights for top genes for Extended Data Fig. 3a, mean log-normalized expression and decoder weights for GPs in Extended Data Fig. 3c,d, normalized entropy of decoder weights for Extended Data Fig. 3e and decoder weights for GPs in Extended Data Fig. 3f,g.
Source Data Extended Data Fig. 4 UMAP coordinates of the latent spaces for downsampled query dataset projected to the reference for Extended Data Fig. 4a, control cells integration metrics values for Extended Data Fig. 4b and Bayes factors for differential GP test between IFN-β-stimulated and control cells for the downsampled query projected to the reference for Extended Data Fig. 4c.
Source Data Extended Data Fig. 5 UMAP coordinates of the latent space for IFN-β dataset for Extended Data Fig. 5a, Bayes factors for differential GP test between IFN-β-stimulated and control cells for Extended Data Fig. 5b, expiMap latent scores for GPs in Extended Data Fig. 5d and Bayes factors for the differential GP test of CD14^+^ monocytes IFN-β-stimulated versus control for Extended Data Fig. 5e.
Source Data Extended Data Fig. 6 Reference integration metrics values for Extended Data Fig. 6a, and integration metrics for downsampled data for Extended Data Fig. 6b.
Source Data Extended Data Fig. 7 Gene membership overlap data for GPs for Extended Data Fig. 7a,b, Bayes scores for the differential GP tests of IFN-β cells versus control in Extended Data Fig. 7c, B cells versus rest in Extended Data Fig. 7d, myeloid cells versus rest in Extended Data Fig. 7e and gene recovery results of deleted genes with soft membership in the IFN-β dataset for Extended Data Fig. 7f,g.
Source Data Extended Data Fig. 8 Log-normalized expression values of genes in the figure.
Source Data Extended Data Fig. 9 UMAP coordinates of the latent spaces for Extended Data Fig. 9a, values for query to reference projection (integration) metrics for Extended Data Fig. 9b, PAGA results for Extended Data Fig. 9c and latent scores of the GPs in Extended Data Fig. 9d.
Source Data Extended Data Fig. 10 UMAP coordinates of the expiMap latent space and latent scores of the GPs in the figure.

## Code availability

The software is available as a part of https://scarches.readthedocs.io/en/latest/. The code to reproduce the results is available at https://github.com/theislab/expiMap_reproducibility.
