# Supplementary material for: Biologically informed deep learning to query gene programs in single-cell atlases
Source: Nat Cell Biol. 2023 Feb 2;25(2):337–50. doi: 10.1038/s41556-022-01072-x (PMC9928587; doi:10.1038/s41556-022-01072-x)
Supplement: Supplementary file 1 — Supplementary Notes 1–12 and Figs. 1–10. [file 41556_2022_1072_MOESM1_ESM.pdf]

nature cell biology

Technical Report

https://doi.org/10.1038/s41556-022-01072-x

Biologically informed deep learning to query gene programs in single-cell atlases

In the format provided by the authors and unedited

Supplementary Information

Supplementary Note 1: comparison with limma-fry

We compared the Bayes factors from the expiMap model to the enrichment results (FDR values) from limma-fry [1, 2] applied to the PBMC IFN-β data using identical terms selected from Reactome.

We considered the following comparisons in the PBMC IFN-β data for the gene set enrichment analysis: terms enriched at the global level in IFN-β stimulated cells compared to control unstimulated cells; terms enriched in B cells, CD14+ Monocytes and CD16+ Monocytes where each of these populations was separately compared to all other cell-types; and terms enriched in CD14+ Monocyte population in IFN-β stimulated cells compared with the control (unstimulated) cells. In each of these comparisons, we added "study" as a covariate in the design matrix of the linear model in the limma-fry framework. The threshold for the absolute natural logarithm of the Bayes scores from expiMap was set to 2.3, which corresponds to a strong evidence for the enrichment of a term in one group of cells compared to another group of cells in a Bayesian hypothesis testing framework. We refer to the results obtained at thresholds larger than this nominated threshold as the "expiMap test results", and shall call such gene programs "differential GPs" in the comparison of interest in this work. The threshold criteria for the limma-fry results was set to a mixed FDR (that is, direction-independent enrichment) value of 0.05. We chose to perform GSEA using the limma-fry pipeline to account for the complex experimental design of the integrated data, which would not otherwise have been possible with rank-based enrichment tests.

We observed that the enrichment results generally agree for both expiMap and the limma-fry framework for GSEA. However, expiMap tends to select differential GPs that are more specialized compared to GPs enriched by fry-based GSEA, where the increased variance of gene expression measurements on the integrated atlas due to biological and technical variability can impede the detection of relevant biological signals. In **Extended Data Fig. 2a**, where IFN-β treated cells are compared with control (untreated) cells, the terms INTERFERON_SIGNALING and INTERFERON_ALPHA_BETA_SIGNALING were found to be enriched in IFN-βstimulated cells in both the expiMap and GSEA results. The limma-fry framework for GSEA identified the enrichment of CYTOKINE_SIGNALING_IN_IMMUNE_SYSTEM and IMMUNE_SYSTEM in IFN-β cells, which are considered broader and less specialized terms than the ones detected by expiMap. The terms enriched in stimulated CD14+ Monocytes were largely similar in the expiMap test and fry results (**Extended Data Fig. 2b**); however, expiMap detected the important pathway METABOLISM_OF_CARBOHYDRATES, which was missed in the fry results. We also observed that fry tends to assign significant scores to the general GPs with larger numbers of genes. For B cells and CD16+ Monocytes (**Extended Data Fig. 2c-d**), fry detects only the general terms, such as ADAPTIVE_IMMUNE_SYSTEM and IMMUNE_SYSTEM (and additionally HEMOSTASIS for CD16+ Monocytes), whereas the expiMap test identified smaller-sized and more specialized GPs for both cell types. For example, for B cells, the expiMap test identifies enriched terms such as SIGNALING_BY_B_CELL_RECEPTOR_BCR and MHC_CLASS_II_ANTIGEN_PRESENTATION (**Extended Data Fig. 2c**). The enriched Reactome GPs were almost identical in the cell type specific test for CD14+ Monocytes (**Extended Data Fig. 2e**) in the expiMap and fry approaches. In addition to an enhanced capability for detection of specialized cellular and molecular programs, expiMap removes the need to repeat differential gene expression and gene set enrichment testing for every single comparison, thereby resulting in a shorter computational time and faster data analysis.

Supplementary Note 2: Dependence between the expression levels of genes and their importance scores

We extracted top important genes according to the gene importance score for the model of the IFN-β dataset mapped onto the healthy immune reference (**Fig 2.**) and calculated the Pearson correlation between gene importance scores and mean log-normalized expression for different numbers of top scored genes within each GP (**Extended Data Fig. 3a**). Overall, we observed a correlation between importance scores and the mean log-normalized expression value. These correlations tend to decrease when we include more genes with lower scores, meaning there is some dependence between importance scores and mean expression but not necessarily linear. This is expected as the reconstruction of cell expression is done with linear weights in the decoder (from which the gene importance scores are obtained) and nonlinearly derived latent scores from the encoder (see the description of the model). We highlighted three different GPs with high positive, high negative, and close to zero correlation to further demonstrate different types of mean/score relationships (**Extended Data Fig. 3b-d**).

We also sought to see if the GPs are dominated by only a few genes or a collection of genes (see **Extended Data Fig. 3e-g**). To assess this, we leveraged normalized entropy (see **Methods**, section **Metrics for integration and evaluation**) of gene importance scores for the top 50 genes (**Extended Data Fig. 3e**). As an example, we compared one low entropy GP (**Extended Data Fig. 3f**, entropy = 0.799) and high entropy (**Extended Data Fig. 3g**, entropy = 0.988), demonstrating GPs with higher entropy are not dominated by few genes. In contrast, in low entropy GPs, the GP score is dominated by fewer genes with high scores. Overall, we observed most GPs had high entropy values implying the heterogeneity of many GPs are not explained by a few genes but by a collection of them.

Supplementary Note 3: Robustness of the model under different data query dataset sizes

In order to evaluate the sensitivity of expiMap to query dataset size, we downsampled the IFN-β up to 10% of the original size (**Extended Data Fig. 4**) and repeated the analogous query to reference mapping as was done for **Fig. 2**. We saw that the model was indeed robust to different dataset sizes. We observed similar integration scores assessing the integration of query control cells into the reference. Our results also implicated that the same GPs explaining control and IFN-β stimulated samples' differences can be recovered consistently across different dataset sizes (**Extended Data Fig. 4c**).

Supplementary Note 4: Comparing reference mapping and non reference mapping analysis for the PBMC IFN-β dataset

We applied expiMap on the IFN-β dataset alone and repeated the analysis as in **Fig. 2**. We noticed that the results were similar to reference mapping. The overall latent space is still able to differentiate control from IFN-β stimulated samples (see **Extended Data Fig. 5a** and **Fig. 2a**). Using the expiMap Bayes test, we observed that the top two GPs that explain the difference between control and IFN-β stimulated samples are similar to what was obtained with the reference mapping (**Extended Data Fig. 5b-c**; **Fig 2b-c**). The top GPs from query data alone are CYTOKINE_SIGNALING_IN_IMMUNE_SYSTEM and INTERFERON_SIGNALING. The first one is a superset of the second GP. Moreover, while one of the GPs (INTERFERON_SIGNALING) is the same for both analyses, the one found in reference mapping (INTERFERON_ALPHA_BETA_SIGNALING) is more specific than CYTOKINE_SIGNALING_IN_IMMUNE_SYSTEM which is indeed the superset of both GPs (INTERFERON_SIGNALING and INTERFERON_ALPHA_BETA_

SIGNALING).

We also compared cell-type specific analysis as shown in **Fig. 2d**. The expiMap test for B cells enriched (i.e. give absolute log Bayes score >= 2.3) (**Supplementary Fig. 10** for B cells) MHC_CLASS_II_ANTIGEN_PRESENTATION which is identical to reference mapping results (**Fig. 2d**, **Extended Data Fig. 1, Supplementary Fig. 1** for B cells). However, the query analysis alone did not enrich SIGNALING_BY_THE_B_CELL_RECEPTOR_BCR and instead found ANTIGEN_ACTIVATES_B_CELL_RECEPTOR_LEADING_TO_GENERATION_OF_SECOND _MESSENGERS and which is similar to SIGNALING_BY_THE_B_CELL_RECEPTOR_BCR as quantified using gene overlap (20 genes out of 37 genes).

For CD14+ Monocytes IFN-β dataset only training gave TRANSMEMBRANE_TRANSPORT_OF_ SMALL_MOLECULES as the top scored GP (**Supplementary Fig. 10**). CD14+ Monocytes also enriched this GP in the query to reference mapping example (**Extended Data Fig. 1**), but it was not the top-scored GP. For CD16+ Monocytes IMMUNOREGULATORY_INTERACTIONS_ BETWEEN_A_LYMPHOID_AND_A_NON_LYMPHOID_CELL was the top scored GP for the IFN-β dataset alone, but this GP was not enriched by CD16+ Monocytes in the query to reference mapping example (**Extended Data Fig. 1**). This is why the cell type separation scatter plots in **Extended Data Fig. 5d** feature GPs different from those in **Fig. 2d**.

While trying to reproduce results from **Fig. 2e**, we observed the enrichment of interferon and GPCR-related GPs (**Extended Data Fig. 5e**. However, METABOLISM_OF_CARBOHYDRATES and important GP for our analysis of the specificity of interferon response in CD14+ Monocytes were not enriched in query-alone results. It was ranked 24th among the top 30 GPs in differential GP analysis in query data alone compared to being among the top 5 in the reference mapping analysis.

Overall, while the two pipelines were similar, but in some cases the reference mapping could enrich more specific GPs.

Supplementary Note 5: Comparison of integration performance

The improvements achieved by reference mapping with expiMap compared with scVI (**Fig. 3**) as one of the top performers in atlas-level integration benchmarks [3] motivated us to investigate this performance gain further. While both scVI and expiMap are variational autoencoders, scVI implements a nonlinear encoder and decoder whereas expiMap uses a linear and lower capacity decoder to enable interpretability. We hypothesized that the additional domain knowledge in expiMap combined with a linear decoder helps to find better posterior distributions in the encoder by improving a well-known problem called the "amortization gap" [4], which is defined as the difference attributable to amortizing variational parameters over all the training data compared with the estimations for individual training samples. This leads to suboptimal variational approximation by the encoder in latent variable variational models [4]. This problem concerns the suboptimal posterior distribution learned. Thus, expiMap would find a richer representation within the family of all possible solutions compared with the less optimal representation found by scVI among more complex families of solutions enabled by additional non-linearities in the model. To test this, we modified the scVI encoder to employ a non-amortized formulation (see the section **Non-amortized scVI**), in which the parameters of the variational distribution are optimized for each cell individually. We trained expiMap, scVI, and non-amortized scVI to construct references with multiple atlases across five tissues obtained from Sfaira [5]. We observed that non-amortized versions of scVI consistently achieved superior or equal performance in data integration compared with expiMap, while remaining better than the default (amortized) scVI, which corroborated our hypothesis (**Fig. 3c**). We further performed similar benchmarking to evaluate our model against linear-decoded variational autoencoder (LDVAE) [6], a variation of scVI with a linear decoder. We found that LDVAE had similar performance to amortized scVI, yet poorer performance than expiMap and non-amortized scVI, demonstrating the importance of including domain knowledge in expiMap (see **Extended**

Data Fig. 6).

Supplementary Note 6: Disentanglement and robustness of newly learned GPs

The de novo GPs leaned by the model for **Fig 4.** are disentangled from previous GPs as we have evaluated using gene overlap analysis in **Fig. 4c** and can capture novel variations in the query data. We also measured Pearson correlation coefficient values between the scores of the new GPs for all cells (**Supplementary Table 6**). The correlations of the scores of the new GPs between each other are primarily low, demonstrating independence. However, high correlations exist between the new GPs and some reference GPs. For example, in addition to new unconstrained Node 1 that enriches for Myeloids (see **Extended Data Fig. 7e** and **Fig. 4d**), there exist other reference GPs that also enrich for Myeloids such as the top two ones (see **Extended Data Fig. 7e**) including TRANSMEMBRANE_TRANSPORT_OF_SMALL_MOLECULES (correlation 0.82 with the scores of Node 1, gene overlap with top 50 genes of node 1 = 3) and METABOLISM_OF_LIPIDS_AND_LIPOPROTEINS (correlation 0.73, gene overlap with top 50 genes of node 1 = 1). They are correlated with Node 1 but they include different genes. Thus, while the correlation might still exist because reference GPs partially explain the variation, they are still limited to prior knowledge. The new GPs can learn the novel source of variations not present in the reference GP. Therefore, although we regularize the new GPs, the complete disentanglement of new GPs from reference might not be entirely possible and desired when the reference GPs partially explain the variation in the query. Still, regularization can help to learn a new set of genes explaining new or existing variations and impose independence among newly discovered GPs.

We also analyzed the performance of new GPs for different scenarios consisting of different query downsampling sizes, different hyperparameters' values, and recovery of different cell types removed from the reference. Overall we observed the robust performance of expiMap across different scenarios and hyperparameters (**Supplementary Tables 7-12**). Moreover, we removed each cell type reported in **Supplementary Table 7** from the reference and ran the model training 20 times for each combination of hyperparameter values and removed cell type. Here, we consider the enrichment of a cell type successful if the built-in Bayesian test for the cell type of interest yields a Bayes score greater than 2.3 for an unconstrained GPs and that unconstrained GP should also be ranked first among all GPs in the test. The results show that for most model runs, all cell types except NK cells are stably recovered by new unconstrained GPs. The failure to recover NK cells could be attributed to the small sample size of these cells in the query dataset.

Next, we analyzed the consistency for the recovery of B cells GP and IFN-β stimulation GP with the exact scenario of cell-type and GP removal as **Fig. 4** but with different hyperparameters and query sample sizes as reported in **Supplementary Tables 8-9**. The number of successful recoveries was evaluated by enrichment of a GP using the expiMap Bayes test for different query sizes and values of L1 regularization for the soft mask (alpha_l1). Specifically, we observed that the new constrained GP for B cell receptor is not enriched for B cells when L1 regularization is low or the sample size is small (see **Supplementary Table 8**). Conversely, increasing the value of L1 regularization (alpha_l1) improves results. Similar results were observed for IFN-β stimulation GP (see **Supplementary Table 9**), but sample size did not affect the results since there was a strong signal across all cell types for this GP.

Further, we evaluated the recovery of GPs related to IFN-β and B cells by allocating different numbers of new unconstrained GPs. This scenario differs from the previous experiment, where we included a B cell receptor-constrained GP. We observed (see **Supplementary Tables 10-11**) that adding more new unconstrained GPs leads to recovery of both IFN-β and B cells GPs and increases GP recovery stability. We also analyzed the recovery of IFN-β stimulation by new unconstrained GPs when the number of IFN-β stimulated cells is reduced (see **Supplementary Table 12**). We observed that IFN-β stimulation GP is recovered for all dataset sizes. These results show that the

model is stable under different sample sizes of IFN-β stimulated population.

Supplementary Note 7: Refinement of pre-defined GPs

As we demonstrated for B cells (**Fig. 4b**), expiMap can enrich predefined and potentially incomplete GPs. We further examined this feature by incrementally removing the most influential genes from general interferon signaling GP-trained, IFN-β-treated cells, and control cells from the data of Kang et al. (IFN-β dataset), while also monotonically increasing the L1 sparsity. Lower L1 values encourage the model to add more genes to the predefined GP than higher L1 values, restricting the model to the predefined features. We observe that the model robustly recovers deleted genes with different ranges of L1 values (see **Extended Data Fig. 7f**); however, for lower L1 values, the model can also incorrectly add genes that may not be in the original program (see **Extended Data Fig. 7g**).

Supplementary Note 8: Pancreas data integration

The data is comprised of young healthy mice on postnatal day 16 (dataset name: Fltp_P16, reference) [7], healthy young mice from a non-obese diabetic model before the onset of type 1 diabetes (T1D) at 5 weeks of age (dataset name: NOD, reference) [8], healthy adult mice and those exposed to chemically-induced stress (dataset name: spikein_drug, reference) [9], and healthy adult mice and those exposed to chemically-induced (streptozotocin-induced) type 2 diabetes (T2D) with and without therapy with different combinations of insulin, GLP1, and estrogen (dataset name: STZ, query) [10].

Our analysis showed that expiMap integration preserves cell subtype variability. Namely, expiMap-based UMAP of beta cells shows the gradual separation of diseased, differently treated, and healthy query cells (**Fig. 6c**) according to known treatment efficacy: GLP-1-estrogen+insulin was the most effective [10]. Healthy beta cells from the reference and query exhibit greater overlap. The same pattern is quantitatively supported by the PAGA graph, which indicated the strongest connection of reference cells with the query control, followed by insulin-treated T2D-model samples, and weaker connections with other T2D-model samples (**Extended Data Fig. 9c**). To evaluate if expiMap has captured the diabetes-associated beta cell loss of identity, dedifferentiation, and transdifferentiation to other endocrine cell types [10, 11], we investigated the relevant cell type GPs (**Extended Data Fig. 9d**). Indeed, in T2D-model beta cells, we observed lower scores for beta cell identity markers in conjunction with higher scores for markers of pancreatic progenitors and enteroendocrine cells (an umbrella term that also encompasses pancreatic endocrine cells). This shows that expiMap can capture heterogeneity within relatively homogenous populations, such as a particular cell type, and thus helps us to interpret why batches do not fully overlap within the embedding. Our analysis thus indicates that expiMap produces high-quality integrations, which can be directly interpreted at the molecular level.

Supplementary Note 9: Pancreas differential GP analysis

When comparing healthy control and T2D beta cells we observed differences in energy metabolism and protein synthesis, the unfolded protein response (UPR), cell-matrix interactions, and cell-cell interactions, including Notch signaling and immune communication. Genes most strongly contributed to the activation of the enriched GP are reported in **Supplementary Table 14**. Some of these differences were already reported in the original study: the diabetes-associated increase in oxidative phosphorylation, electron transport chain activity, and endoplasmic reticulum stress, which is related to the UPR [10]. The remaining differences were not highlighted in the original study, although other studies observed a diabetes-related increase in insulin protein synthesis [12], Notch signaling

[13], disrupted islet architecture [14], and immune infiltration into islets [15]. Together, these results demonstrate that expiMap enrichment could identify diabetes-associated molecular changes in the T2D-model cells.

Supplementary Note 10: UPR and N-linked glycosylation analysis

The observed correlation between the UPR and N-linked glycosylation across various beta cell states from different studies may implicate a shared regulatory mechanism between the UPR and N-linked glycosylation in beta cells. Indeed, it has been previously shown that the UPR regulator XBP1 affects N-glycan structures [16]; however, to our knowledge, we are the first to report this correlation in pancreatic beta cells. It was suggested that these changes in N-glycans on the cell surface might also be involved in cellular signaling and the immune response [16]. Thus, we checked if immune-related GPs differentially active in T2D-model beta cells correlate with N-linked glycosylation, as it is known that immune infiltration plays a role in T2D and may be caused by metabolic stress and changed epitopes on beta cells [15, 17]. Again, we observed a strong correlation between N-linked glycosylation and the innate immune system GP scores (absolute correlation coefficient of 0.77) across datasets (**Fig. 6h**), but the correlation with other enriched immune-related GPs was lower (**Supplementary Fig. 9a**).

As the GPs are not intrinsically directed, we assessed the direction of correlation of GPs N-linked glycosylation and innate immune system with their genes (**Supplementary Fig. 9b**). Most influential genes from the GP innate immune system were negatively correlated with their GP (**Supplementary Fig. 9b**) and correlation of individual genes with the highest absolute weights between the two GPs was either highly positive or around zero (**Supplementary Fig. 9c**). These correlations of individual genes were much lower than the correlation on the GP-level (**Fig. 6h**), indicating the benefit of looking at GPs rather than individual genes, as they are more strongly affected by noise, such as due to expression dropout [18].

We further assessed the contribution of individual genes to the GP score. Genes with the highest absolute weights within a given GP generally exhibited high correlation with the GP (**Supplementary Fig. 9b**). Importantly, the GP scores were driven by multiple genes, as indicated by the high correlation of individual GP scores with several member genes and their high gene weights (**Supplementary Fig. 9b**). However, genes expressed in more cells had, in general, higher GP correlation. Thus GPs that had mainly relatively sparsely expressed genes were more affected by individual highly expressed genes, as also indicated by their gene weights (**Supplementary Fig. 9b**).

Supplementary Note 11: Mouse endocrinogenesis

To further demonstrate the utility of expiMap beyond discrete systems, we have applied expiMap on a developmental dataset from mouse endocrinogenesis [19] across four time points (**Extended Data Fig. 10a**). This dataset represents a continuous process to assess expiMap's utility and applicability further to infer developmental stages using GPs. We trained a reference model on embryonic days (E)14.5 and (E)15.5 and mapped earlier time points as a query. The query dataset integrated well into the reference, and our analysis of GPs illuminated major compartments (**Extended Data Fig. 10b-g**) of the cycling population of ductal and endocrine progenitors and cell-cycle exit and differentiation trajectory toward terminal alpha, beta, delta, and epsilon fates [20]. Our GP-based analysis is further corroborated by performing velocity analysis highlighting similar populations as previously reported by Bergen et al. [20] These results demonstrate that the expiMap GP activity analysis captures a complex differentiation process.

Supplementary Note 12: Hyperparameters

This section describes the hyperparameters used for the training models in different scenarios.

Experiment	Condition label
Immune Atlas (reference) + PBMC IFN-β (query)	study
Immune Atlas (reference) + PBMC IFN-β & COVID (query)	study
Pancreas	study_sample
PBMC IFN-β (deleted genes recovery)	study
PBMCs (Integration evaluation)	donor
Heart (Integration evaluation)	cell_source
Lung (Integration evaluation)	tech_sample
Colon (Integration evaluation)	Source
Liver (Integration evaluation)	orig.ident
Mouse endocrinogenesis	day

Hyperparameter Data 1 | The label type used for each experiment as conditions for expiMap models.

Experiment	soft mask	group lasso	new GPs
Immune Atlas (reference) + PBMC IFN-β (query)	No	Yes	No
Immune Atlas (reference) + PBMC IFN-β (query)	Yes (query)	Yes	Yes (3 in query)
Immune Atlas (reference) + PBMC IFN-β & COVID (query)	No	Yes	No
Pancreas	No	Yes	No
PBMC IFN-β (deleted genes recovery)	Yes	Yes	No
Integration evaluation	No	Yes	No
Mouse endocrinogenesis	No	Yes	No

Hyperparameter Data 2 | Usage of features for each experiment.

Name	Operation	NoF/Kernel Dim.	Dropout	LN	Activation	Input
**Inputs**						
data	-	#Genes	×	×	-	-
conditions	-	#Conditions	×	×	-	-
**Encoder**						
Layer_1	FC	256	0.05	✓	ReLU	[data, condition labels]
Layer_2	FC	256	0.05	✓	ReLU	Layer_1
Layer_3	FC	256	0.05	✓	ReLU	Layer_2
mean	FC	#Gene Programs	×	×	Linear	Layer_3
var	FC	#Gene Programs	×	×	Linear	Layer_3
latent	Multivariate Normal	#Gene Programs	×	×	-	[mean, var]
**Decoder**						
Layer_1	FC	#Genes	×	×	softmax	[latent, condition labels]
predicted count mean	Multiplication	#Genes	×	×	-	[Layer_1, library scale]
**Hyperparameters**						
Loss	NB					
Optimizer	Adam					
Learning Rate	0.001					
epsilon	0.01					
Batch Size	128					
# of Epochs	max. 400					
alpha (group lasso weight)	0.7					
alpha_kl (KL term weight)	0.5 (reference)	0.1 (query)				
alpha_epoch_anneal (epochs for alpha_kl annealing)	100					

Hyperparameter Data 3 | expiMap detailed architecture for the Immune Atlas (reference) + PBMC IFN-β (query) experiment (**Fig. 2**).

Name	Operation	NoF/Kernel Dim.	Dropout	LN	Activation	Input
**Inputs**						
data	-	#Genes	×	×	-	-
conditions	-	#Conditions	×	×	-	-
**Encoder**						
Layer_1	FC	300	0.05	✓	ReLU	[data, condition labels]
Layer_2	FC	300	0.05	✓	ReLU	Layer_1
Layer_3	FC	300	0.05	✓	ReLU	Layer_2
mean	FC	#Gene Programs	×	×	Linear	Layer_3
var	FC	#Gene Programs	×	×	Linear	Layer_3
latent	Multivariate Normal	#Gene Programs	×	×	-	[mean, var]
**Decoder**						
Layer_1	FC	#Genes	×	×	softmax	[latent, condition labels]
predicted count mean	Multiplication	#Genes	×	×	-	[Layer_1, library scale]
**Hyperparameters**						
Loss	NB					
Optimizer	Adam					
Learning Rate	0.001					
epsilon	0.01					
Batch Size	128					
# of Epochs	max. 200 (reference)	150 (query)				
alpha (group lasso weight)	0.7					
gamma_ext (L1 regularization weight for new unconstraind nodes)	-	0.7 (query)				
gamma_epoch_anneal (epochs for gamma_ext annealing)	-	50 (query)				
alpha_kl (KL term weight)	0.5 (reference)	0.1 (query)				
alpha_epoch_anneal (epochs for alpha_kl annealing)	100 (reference)	50 (query)				
beta (HSIC weight for new unconstrained nodes)	-	3 (query)				
alpha_l1 (soft mask weight for new constrained nodes)	-	0.9 (query)				
3 unconstrained and 1 constrained new GPs in query						

Hyperparameter Data 4 | expiMap detailed architecture for the Immune Atlas (reference) + PBMC IFN-β (query) learning new GPs experiment (**Fig. 4**). Also for **supplementary tables 7-12** with differences in hyperparameters indicated in these tables.

Name	Operation	NoF/Kernel Dim.	Dropout	LN	Activation	Input
**I**nputs						
data	-	#Genes	×	×	-	-
conditions	-	#Conditions	×	×	-	-
**E**ncoder						
Layer_1	FC	256	0.05	✓	ReLU	[data, **c**ondition labels]
Layer_2	FC	256	0.05	✓	ReLU	Layer_1
Layer_3	FC	256	0.05	✓	ReLU	Layer_2
mean	FC	#Gene Programs	×	×	Linear	Layer_3
var	FC	#Gene Programs	×	×	Linear	Layer_3
latent	Multivariate Normal	#Gene Programs	×	×	-	[mean, var]
**L**ibrary Encoder						
Layer_1	FC	128	0.05	✓	ReLU	[data, **c**ondition labels]
mean	FC	1	×	×	Linear	Layer_1
var	FC	1	×	×	Linear	Layer_1
sizefactors	Normal	1	×	×	-	[mean, var]
**D**ecoder						
Layer_1	FC	#Genes	×	×	softmax	[latent, **c**ondition labels]
predicted count mean	Multiplication	#Genes	×	×	-	[Layer_1, sizefactors]
**H**yperparameters						
Loss	NB					
Optimizer	Adam					
Learning Rate	0.001					
epsilon	0.01					
Batch Size	128					
# of Epochs	max. 400 (reference)	max. 200 (query)				
alpha (group lasso weight)	0.7					
alpha_kl (KL term weight)	0.5 (reference)	0.1 (query)				
alpha_epoch_anneal (epochs for alpha_kl annealing)	100 (reference)	50 (query)				

Hyperparameter Data 5 | expiMap detailed architecture for the Immune Atlas (reference) + PBMC IFN-β & COVID (query) experiment (**Fig. 5**).

Name	Operation	NoF/Kernel Dim.	Dropout	LN	Activation	Input
**I**nputs						
data	-	#Genes	×	×	-	-
conditions	-	#Conditions	×	×	-	-
**E**ncoder						
Layer_1	FC	830	0.05	✓	ReLU	[data, **c**ondition labels]
Layer_2	FC	830	0.05	✓	ReLU	Layer_1
Layer_3	FC	830	0.05	✓	ReLU	Layer_2
mean	FC	#Gene Programs	×	×	Linear	Layer_3
var	FC	#Gene Programs	×	×	Linear	Layer_3
latent	Multivariate Normal	#Gene Programs	×	×	-	[mean, var]
**D**ecoder						
predicted count mean	FC	#Genes	×	×	softplus	[latent, **c**ondition labels]
**H**yperparameters						
Loss	NB					
Optimizer	Adam					
Learning Rate	0.001					
epsilon	0.01					
Batch Size	128					
# of Epochs	max. 500					
omega (used for different group lasso weights for different GPs)	0 for PanglaoDB GPs	1 for Reactome GPs				
alpha_kl (KL term weight)	0.1					
alpha_epoch_anneal (epochs for alpha_kl annealing)	100 (referene)	-				

Hyperparameter Data 6 | expiMap detailed architecture for the Pancreas experiment (**Fig. 6**).

Name	Operation	NoF/Kernel Dim.	Dropout	LN	Activation	Input
**Inputs**						
data	-	#Genes	×	×	-	-
conditions	-	#Conditions	×	×	-	-
**Encoder**						
Layer_1	FC	256	0.05	√	ReLU	[data, condition labels]
Layer_2	FC	256	0.05	√	ReLU	Layer_1
Layer_3	FC	256	0.05	√	ReLU	Layer_2
mean	FC	#Gene Programs	×	×	Linear	Layer_3
var	FC	#Gene Programs	×	×	Linear	Layer_3
latent	Multivariate Normal	#Gene Programs	×	×	-	[mean, var]
**Decoder**						
Layer_1	FC	#Genes	×	×	softmax	[latent, condition labels]
predicted count mean	Multiplication	#Genes	×	×	-	[Layer_1, library scale]
**Hyperparameters**						
Loss	NB					
Optimizer	Adam					
Learning Rate	0.001					
epsilon	0.01					
Batch Size	128					
# of Epochs	max. 200					
alpha (group lasso weight)	0.7					
alpha_kl (KL term weight)	0.06					
alpha_epoch_anneal (epochs for alpha_kl annealing)	100					
alpha_l1 (soft mask weight)	0.4, 0.3, 0.2, 0.1, 0.06					

Hyperparameter Data 7 | expiMap detailed architecture for the PBMC IFN-β deleted genes recovery experiment (**Extended Data Fig. 7f-g**).

Name	Operation	NoF/Kernel Dim.	Dropout	LN	Activation	Input
**Inputs**						
data	-	#Genes	×	×	-	-
conditions	-	#Conditions	×	×	-	-
**Encoder**						
Layer_1	FC	256	0.05	√	ReLU	[data, condition labels]
Layer_2	FC	256	0.05	√	ReLU	Layer_1
Layer_3	FC	256	0.05	√	ReLU	Layer_2
mean	FC	#Gene Programs	×	×	Linear	Layer_3
var	FC	#Gene Programs	×	×	Linear	Layer_3
latent	Multivariate Normal	#Gene Programs	×	×	-	[mean, var]
**Decoder**						
Layer_1	FC	#Genes	×	×	softmax	[latent, condition labels]
predicted count mean	Multiplication	#Genes	×	×	-	[Layer_1, library scale]
**Hyperparameters**						
Loss	NB					
Optimizer	Adam					
Learning Rate	0.001					
epsilon	0.01					
Batch Size	128					
# of Epochs	max. 500					
alpha (group lasso weight)	0.7					
alpha_kl (KL term weight)	0.1					
alpha_epoch_anneal (epochs for alpha_kl annealing)	100					

Hyperparameter Data 8 | expiMap detailed architecture for the integration experiment (**Fig. 3c**, **Extended Data Fig. 6a**).

Name	Operation	NoF/Kernel Dim.	Dropout	BN	Activation	Input
**Inputs**						
data	-	#Genes	×	×	-	-
conditions	-	#Conditions	×	×	-	-
**Encoder**						
mean	Parameter	#Cells × 10	×	×	-	-
var	Parameter	#Cells × 10	×	×	-	-
latent	Multivariate Normal	10	×	×	-	[mean, var]
**Decoder**						
Layer_1	FC	128	×	√	ReLU	[latent, condition labels]
px_r_decoder	FC	#Genes	×	×	Linear	Layer_1
px_dropout_decoder	FC	#Genes	×	×	Linear	Layer_1
px_scale_decoder	FC	#Genes	×	×	softmax	Layer_1
predicted count mean	Multiplication	#Genes	×	×	-	[px_scale_decoder, library scale]
**Hyperparameters**						
Loss	ZINB					
Optimizer	Adam					
Learning Rate	0.05					
epsilon	0.01					
Batch Size	256					
# of Epochs	600					
Weight Decay	1e-6					
mean init	PCA					

Hyperparameter Data 9 | Non-amortized scVI detailed architecture for the integration experiment (**Fig. 3c**, **Extended Data Fig. 6a**).

Name	Operation	NoF/Kernel Dim.	Dropout	BN	Activation	Input
Inputs						
data	-	#Genes	×	×	-	-
conditions	-	#Conditions	×	×	-	-
Encoder						
Layer_1	FC	128	0.1	✓	ReLU	data
mean	FC	10	×	×	Linear	Layer_1
var	FC	10	×	×	Linear	Layer_1
latent	Multivariate Normal	10	×	×	-	[mean, var]
Decoder						
Layer_1	FC	128	×	✓	ReLU	[latent, condition labels]
px_r_decoder	FC	#Genes	×	×	Linear	Layer_1
px_dropout_decoder	FC	#Genes	×	×	Linear	Layer_1
px_scale_decoder	FC	#Genes	×	×	softmax	Layer_1
predicted count mean	Multiplication	#Genes	×	×	-	[px_scale_decoder, library scale]
Hyperparameters						
Loss	ZINB					
Optimizer	Adam					
Learning Rate	0.001					
epsilon	0.01					
Batch Size	128					
# of Epochs	max. 500					
Weight Decay	1e-6					

Hyperparameter Data 10 | scVI detailed architecture for the integration experiment (**Fig. 3c**, **Extended Data Fig. 6a**).

Name	Operation	NoF/Kernel Dim.	Dropout	BN	Activation	Input
Inputs						
data	-	#Genes	×	×	-	-
conditions	-	#Conditions	×	×	-	-
Encoder						
Layer_1	FC	128	0.1	✓	ReLU	data
mean	FC	10	×	×	Linear	Layer_1
var	FC	10	×	×	Linear	Layer_1
latent	Multivariate Normal	10	×	×	-	[mean, var]
Decoder						
px_dropout_decoder	FC	#Genes	×	✓	Linear	[latent, condition labels]
factor_regressor	FC	#Genes	×	✓	softmax	[latent, condition labels]
predicted count mean	Multiplication	#Genes	×	×	-	[factor_regressor, library scale]
Hyperparameters						
Loss	NB					
Optimizer	Adam					
Learning Rate	0.001					
epsilon	0.01					
Batch Size	128					
# of Epochs	max. 500					
Weight Decay	1e-6					

Hyperparameter Data 11 | Linear scVI (LDVAE) detailed architecture for the integration experiment (**Extended Data Fig. 6a**).

Name	Operation	NoF/Kernel Dim.	Dropout	LN	Activation	Input
Inputs						
data	-	#Genes	×	×	-	-
conditions	-	#Conditions	×	×	-	-
Encoder						
Layer_1	FC	128	0.1	✓	ReLU	[data, condition labels]
Layer_2	FC	128	0.1	✓	ReLU	Layer_1
mean	FC	10	×	×	Linear	Layer_2
var	FC	10	×	×	Linear	Layer_2
latent	Multivariate Normal	10	×	×	-	[mean, var]
Decoder						
Layer_1	FC	128	×	✓	ReLU	[latent, condition labels]
Layer_2	FC	128	×	✓	ReLU	Layer_1
px_r_decoder	FC	#Genes	×	×	Linear	Layer_2
px_dropout_decoder	FC	#Genes	×	×	Linear	Layer_2
px_scale_decoder	FC	#Genes	×	×	softmax	Layer_2
predicted count mean	Multiplication	#Genes	×	×	-	[px_scale_decoder, library scale]
Hyperparameters						
Loss	ZINB					
Optimizer	Adam					
Learning Rate	0.001					
epsilon	0.01					
Batch Size	128					
# of Epochs	max. 500					
Weight Decay	1e-6					

Hyperparameter Data 12 | scVI detailed architecture for the query to reference projection (**Fig. 3a-b** and **Supplementary Fig. 4**, **Extended Data Fig. 9b**).

Name	Operation	NoF/Kernel Dim.	Dropout	LN	Activation	Input
**Inputs**						
data	-	#Genes	×	×	-	-
conditions	-	#Conditions	×	×	-	-
**Encoder**						
Layer_1	FC	1000	0.05	√	ReLU	[data, condition labels]
Layer_2	FC	600	0.05	√	ReLU	Layer_1
Layer_3	FC	600	0.05	√	ReLU	Layer_2
mean	FC	#Gene Programs	×	×	Linear	Layer_3
var	FC	#Gene Programs	×	×	Linear	Layer_3
latent	Multivariate Normal	#Gene Programs	×	×	-	[mean, var]
**Decoder**						
Layer_1	FC	#Genes	×	×	softmax	[latent, condition labels]
predicted count mean	Multiplication	#Genes	×	×	-	[Layer_1, library scale]
**Hyperparameters**						
Loss	NB					
Optimizer	Adam					
Learning Rate	0.001					
epsilon	0.01					
Batch Size	128					
# of Epochs	max. 400					
alpha (group lasso weight)	0.7					
alpha_kl (KL term weight)	0.2 (reference)	0.1 (query)				
alpha_epoch_anneal (epochs for alpha_kl annealing)	100					

Hyperparameter Data 13 | expiMap detailed architecture for Mouse endocrinogenesis experiment (**Extended Data Fig. 10**).

Name	Operation	NoF/Kernel Dim.	Dropout	LN	Activation	Input
**Inputs**						
data	-	#Genes	×	×	-	-
conditions	-	#Conditions	×	×	-	-
**Encoder**						
Layer_1	FC	300	0.05	√	ReLU	[data, condition labels]
Layer_2	FC	300	0.05	√	ReLU	Layer_1
Layer_3	FC	300	0.05	√	ReLU	Layer_2
mean	FC	#Gene Programs	×	×	Linear	Layer_3
var	FC	#Gene Programs	×	×	Linear	Layer_3
latent	Multivariate Normal	#Gene Programs	×	×	-	[mean, var]
**Decoder**						
Layer_1	FC	#Genes	×	×	softmax	[latent, condition labels]
predicted count mean	Multiplication	#Genes	×	×	-	[Layer_1, library scale]
**Hyperparameters**						
Loss	NB					
Optimizer	Adam					
Learning Rate	0.001					
epsilon	0.01					
Batch Size	128					
# of Epochs	max. 200					
alpha (group lasso weight)	0.3					
alpha_kl (KL term weight)	0.25					
alpha_epoch_anneal (epochs for alpha_kl annealing)	100					

Hyperparameter Data 14 | expiMap detailed architecture for only IFN-β dataset training (**Extended Data Fig. 5**).

Name	Operation	NoF/Kernel Dim.	Dropout	LN	Activation	Input
**Inputs**						
data	-	#Genes	×	×	-	-
conditions	-	#Conditions	×	×	-	-
**Encoder**						
Layer_1	FC	256	0.05	√	ReLU	[data, condition labels]
Layer_2	FC	256	0.05	√	ReLU	Layer_1
Layer_3	FC	256	0.05	√	ReLU	Layer_2
mean	FC	#Gene Programs	×	×	Linear	Layer_3
var	FC	#Gene Programs	×	×	Linear	Layer_3
latent	Multivariate Normal	#Gene Programs	×	×	-	[mean, var]
**Decoder**						
Layer_1	FC	#Genes	×	×	softmax	[latent, condition labels]
predicted count mean	Multiplication	#Genes	×	×	-	[Layer_1, library scale]
**Hyperparameters**						
Loss	NB					
Optimizer	Adam					
Learning Rate	0.001					
epsilon	0.01					
Batch Size	128					
# of Epochs	max. 400 (reference)	190 (query fraction 0.5)	400 (0.25)	620 (0.1)		
alpha (group lasso weight)	0.7					
alpha_kl (KL term weight)	0.5 (reference)	0.1 (query)				
alpha_epoch_anneal (epochs for alpha_kl annealing)	100					

Hyperparameter Data 15 | expiMap detailed architecture for the Immune Atlas (reference) + downsampled PBMC IFN-β (query) experiment (**Extended Data Fig. 4**).

References

[1] Chen, Y., Lun, A. T. & Smyth, G. K. From reads to genes to pathways: differential expression analysis of rna-seq experiments using rsubread and the edger quasi-likelihood pipeline. F1000Research **5** (2016).

[2] Ritchie, M. E. et al. limma powers differential expression analyses for rna-sequencing and microarray studies. Nucleic acids research **43**, e47–e47 (2015).

[3] Lücken, M. et al. Benchmarking atlas-level data integration in single-cell genomics. Nature Methods **19** (2022).

[4] Cremer, C., Li, X. & Duvenaud, D. Inference suboptimality in variational autoencoders. arXiv preprint arXiv:1801.03558 (2018).

[5] Fischer, D. et al. Sfaira accelerates data and model reuse in single cell genomics. Genome Biology **22** (2021).

[6] Svensson, V., Gayoso, A., Yosef, N. & Pachter, L. Interpretable factor models of single-cell rna-seq via variational autoencoders. Bioinformatics (Oxford, England) **36** (2020).

[7] Salinno, C. et al. Cd81 marks immature and dedifferentiated pancreatic β-cells. Molecular Metabolism **49**, 101188 (2021).

[8] Lee, H. et al. Beta cell dedifferentiation induced by ire1α deletion prevents type 1 diabetes. Cell Metabolism **31** (2020).

[9] Marquina-Sanchez, B. et al. Single-cell rna-seq with spike-in cells enables accurate quantification of cell-specific drug effects in pancreatic islets. Genome Biology **21** (2020).

[10] Sachs, S. et al. Targeted pharmacological therapy restores β-cell function for diabetes remission. Nature Metabolism **2**, 192–209 (2020).

[11] Moin, A. & Butler, A. Alterations in beta cell identity in type 1 and type 2 diabetes. Current Diabetes Reports **19** (2019).

[12] Boland, B., Rhodes, C. & Grimsby, J. The dynamic plasticity of insulin production in β-cells. Molecular Metabolism **6** (2017).

[13] Billiard, F. et al. Delta-like ligand-4-notch signaling inhibition regulates pancreatic islet function and insulin secretion. Cell Reports **22**, 895–904 (2018).

[14] Kilimnik, G. et al. Altered islet composition and disproportionate loss of large islets in patients with type 2 diabetes. PloS one **6**, e27445 (2011).

[15] Böni-Schnetzler, M. & Zeman-Meier, D. Islet inflammation in type 2 diabetes. Seminars in Immunopathology **41** (2019).

[16] Wong, M. et al. Xbp1s activation can globally remodel n-glycan structure distribution patterns. Proceedings of the National Academy of Sciences **115**, 201805425 (2018).

[17] Roep, B., Thomaidou, S., Tienhoven, R. & Zaldumbide, A. Type 1 diabetes mellitus as a disease of the β-cell (do not blame the immune system?). Nature Reviews Endocrinology **17** (2020).

[18] Xu, H., Hu, Y., Aouizerat, B., Yan, C. & Xu, K. A novel graph-based k-partitioning approach improves the detection of gene-gene correlations by single-cell rna sequencing. BMC Genomics **23** (2022).

[19] Bastidas-Ponce, A. et al. Comprehensive single cell mRNA profiling reveals a detailed roadmap for pancreatic endocrinogenesis. Development **146** (2019). Publisher: Oxford University Press for The Company of Biologists Limited Section: RESEARCH ARTICLE.

[20] Bergen, V., Lange, M., Peidli, S., Wolf, F. A. & Theis, F. J. Generalizing RNA velocity to transient cell states through dynamical modeling. Nature Biotechnology **38**, 1408–1414 (2020). Number: 12 Publisher: Nature Publishing Group.

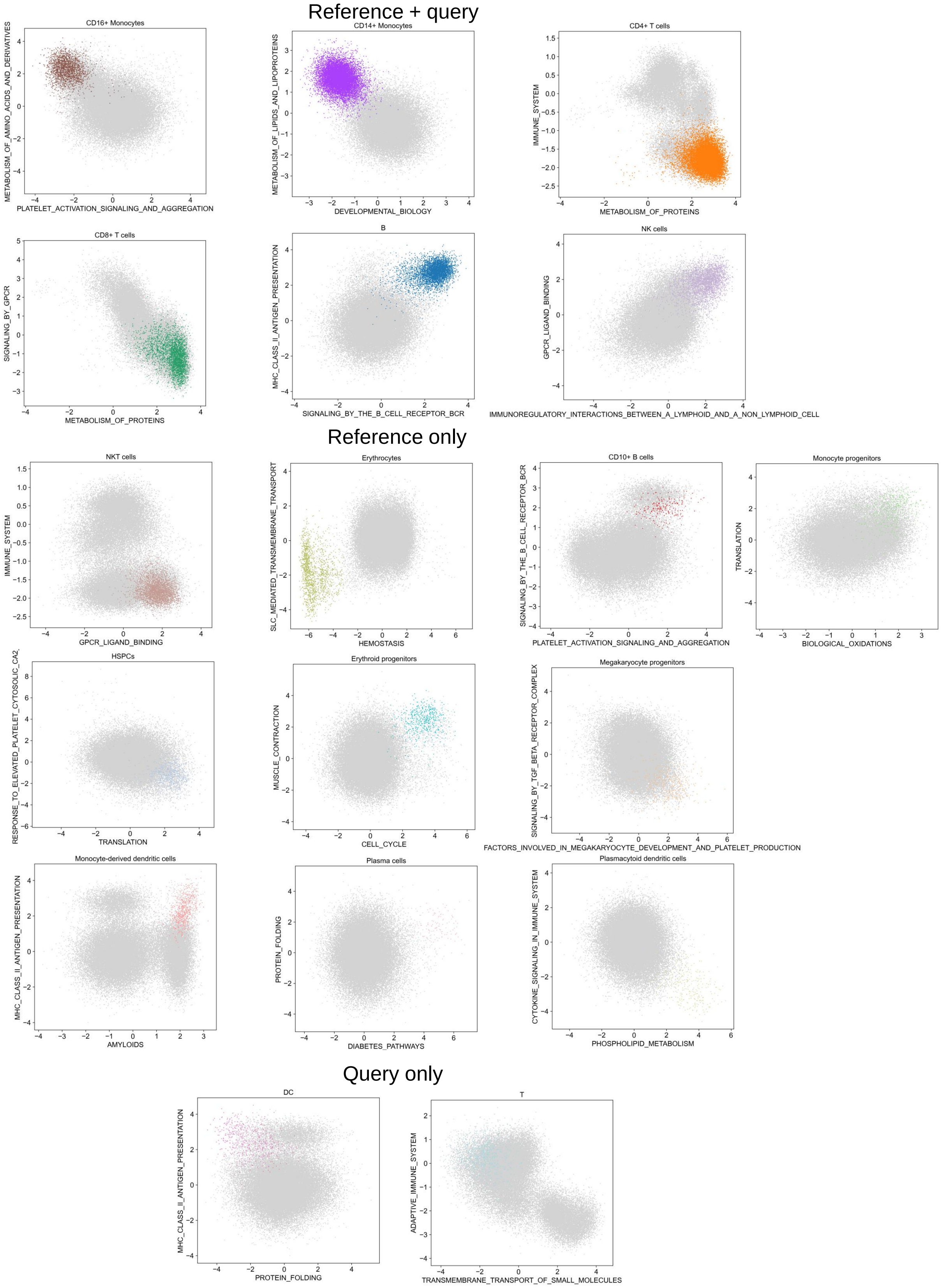

Supplementary Figure 1 | Differential GP analysis results for cell states. Two-dimensional visualization of the top two GPs resulting from the cell type differential analysis results for cell types in the query, the reference, and shared among both. The colors highlight the cell types in the title of each panel while other cells are colored gray.

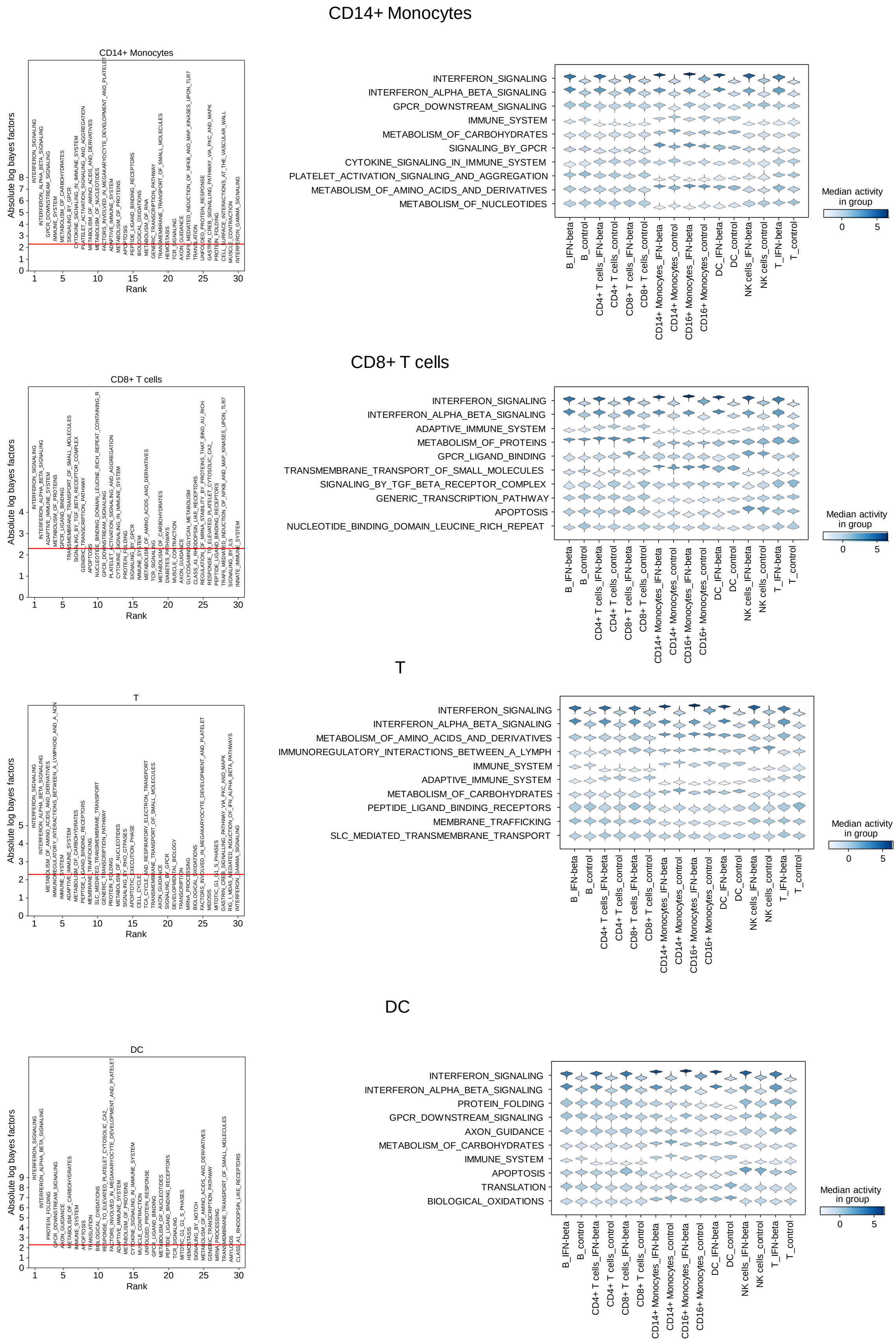

Supplementary Figure 2 | Cell type-specific differential GP analysis for cell states. Differential GP analysis results between IFN-β and control for CD14+ Monocytes, CD8+ T cells, T cells, DC. The x-axis shows the ranking of GPs; the y-axis denotes the significance (absolute log-Bayes factor) of each GP. The violin plots demonstrate the activity of those GPs across other cell types.

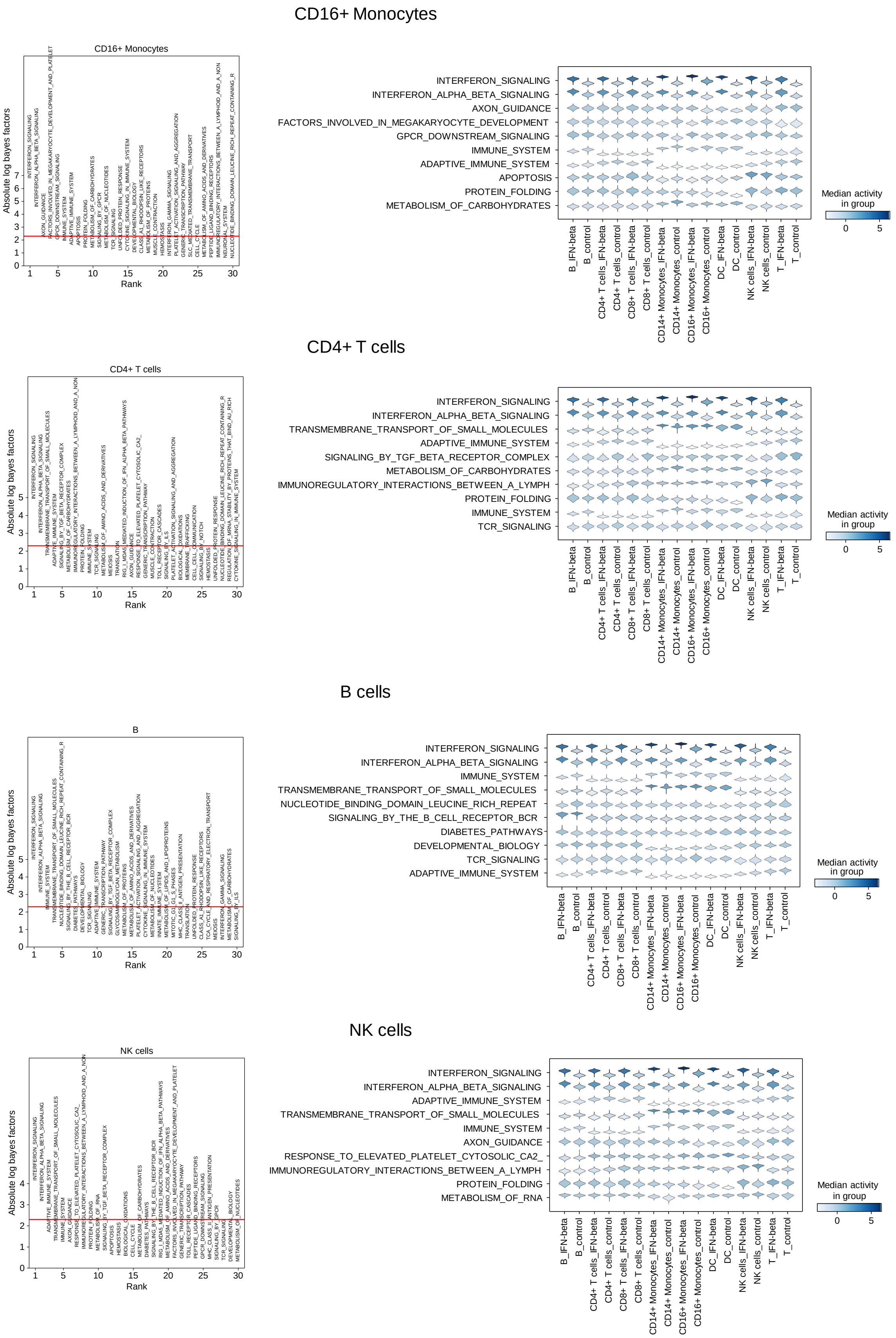

Supplementary Figure 3 | Cell type-specific differential GP analysis for cell states. Differential GP analysis results between IFN-β and control for CD16+ Monocytes, CD4+ T cells, B cells, NK cells. The x-axis shows the ranking of GPs; the y-axis denotes the significance (absolute log-Bayes factor) of each GP. The violin plots demonstrate the activity of those GPs across other cell types.

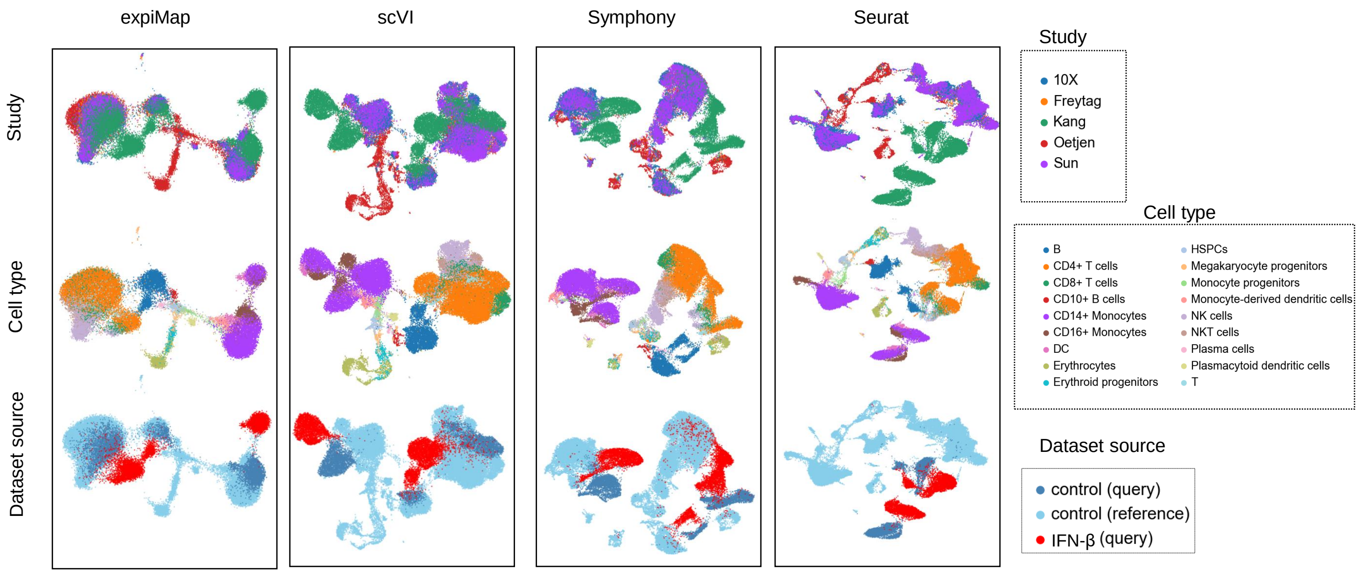

Supplementary Figure 4 | Benchmarking reference mapping methods. UMAP representation of integration accuracy of mapping IFN-β data onto the healthy atlas across studies, cell types, and dataset source for different models.

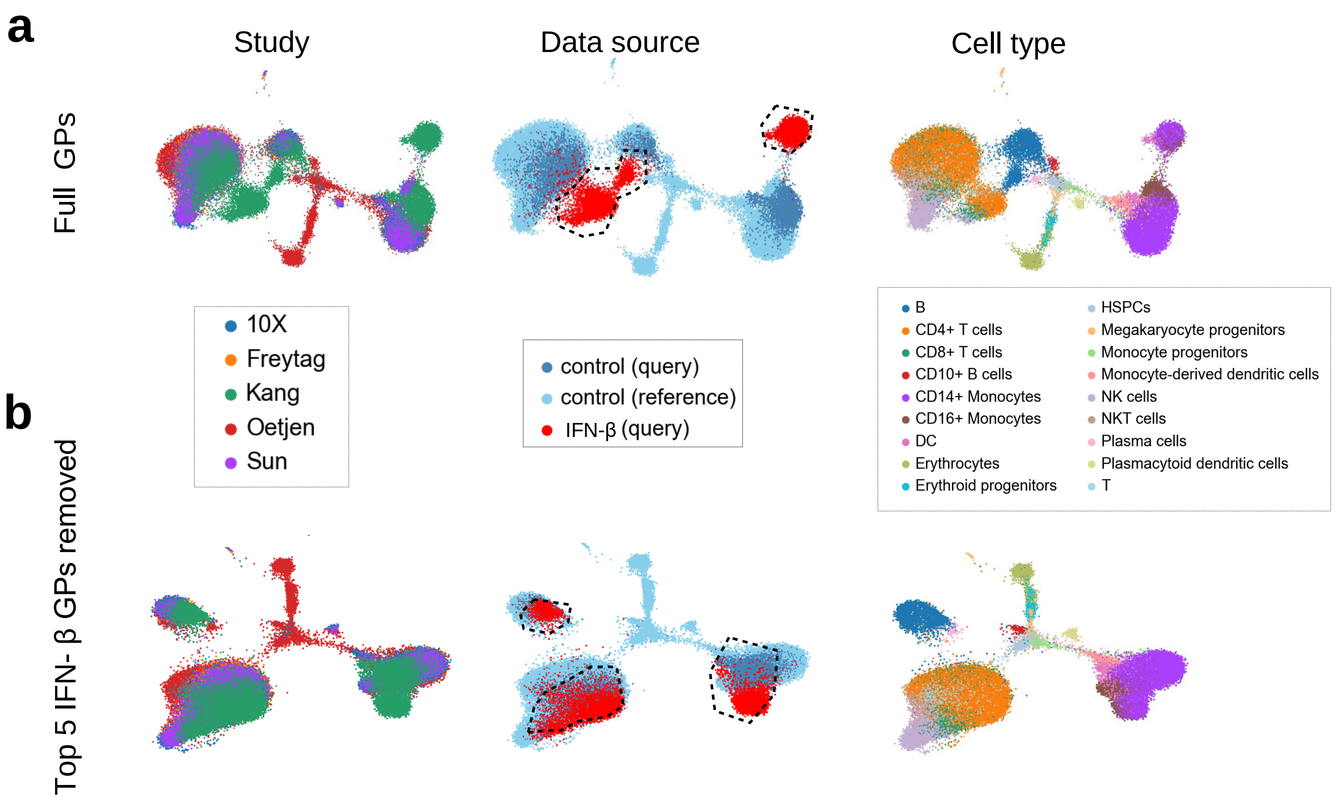

Supplementary Figure 5 | Assessing the quality of reference mapping by removing GPs. (a) Integration reference and query representation across studies, data source, and cell types when expiMap is trained with all available GPs. The highlighted populations are IFN-β-stimulated cells separated from the control samples. **(b)** Same model as **(a)** but the top five IFN-β stimulation-related pathways were removed from training and the model is unaware of them. The highlighted populations are IFN-β-stimulated cells that the model incorrectly merged into control cells, removing perturbation heterogeneity in the query data.

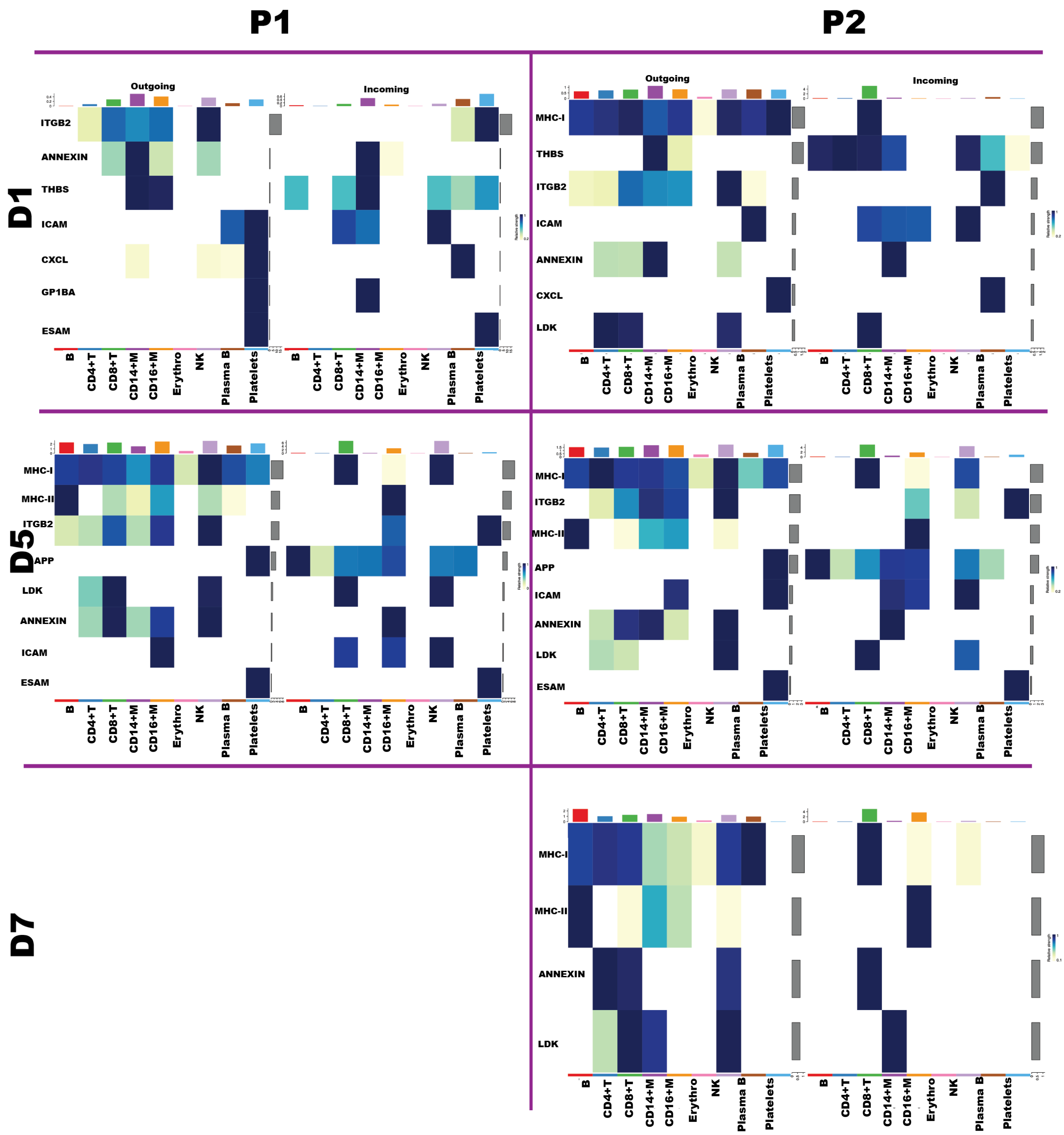

Supplementary Figure 6 | Cell–cell communication pathways in PBMCs from two patients with COVID. The heat maps show the activated cell communication circuits in two patients treated with tocilizumab during the course of admission into the ICU with COVID infection. Each column provides information for each patient and each row provides data for each day of treatment. B = B cells, CD4+ T = CD4+ T cells, CD8+ T = CD8+ T cells, CD14+ M = CD14+ monocytes, CD16+ M = CD16+ monocytes, Erythro = Erythrocytes, Plasma B = Plasma B cells, Platelets.

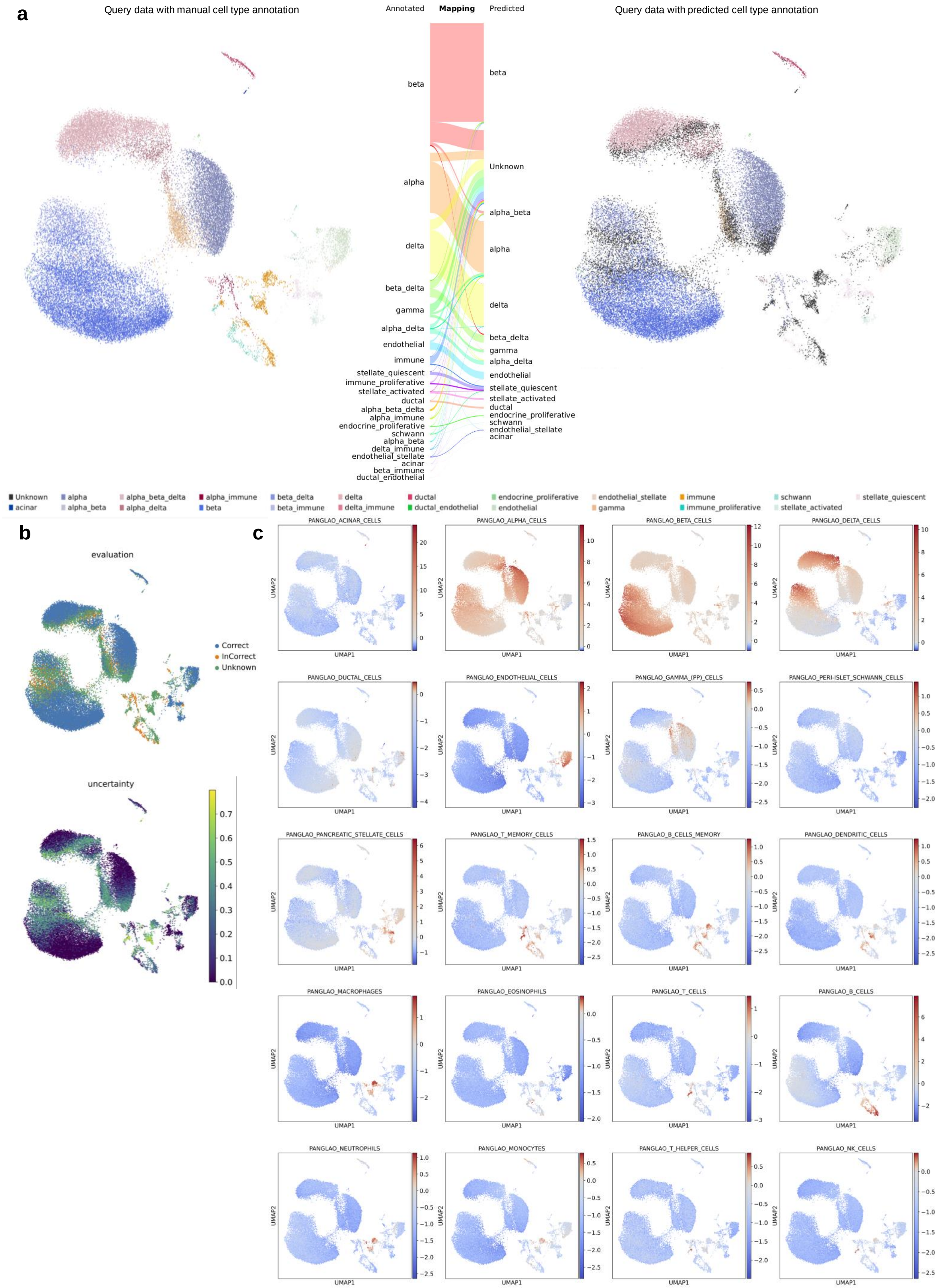

Supplementary Figure 7 | Cell type annotation based on expiMap with automatic annotation transfer and the use of cell type-specific gene set scores. **(a)** Correspondence between manual and transferred annotations in query. UMAPs were calculated using expiMap terms from PanglaoDB. **(b)** Annotation transfer success and uncertainty in query. **(c)** expiMap scores of cell types known to be present in the pancreas can be used for manual cell type annotation.

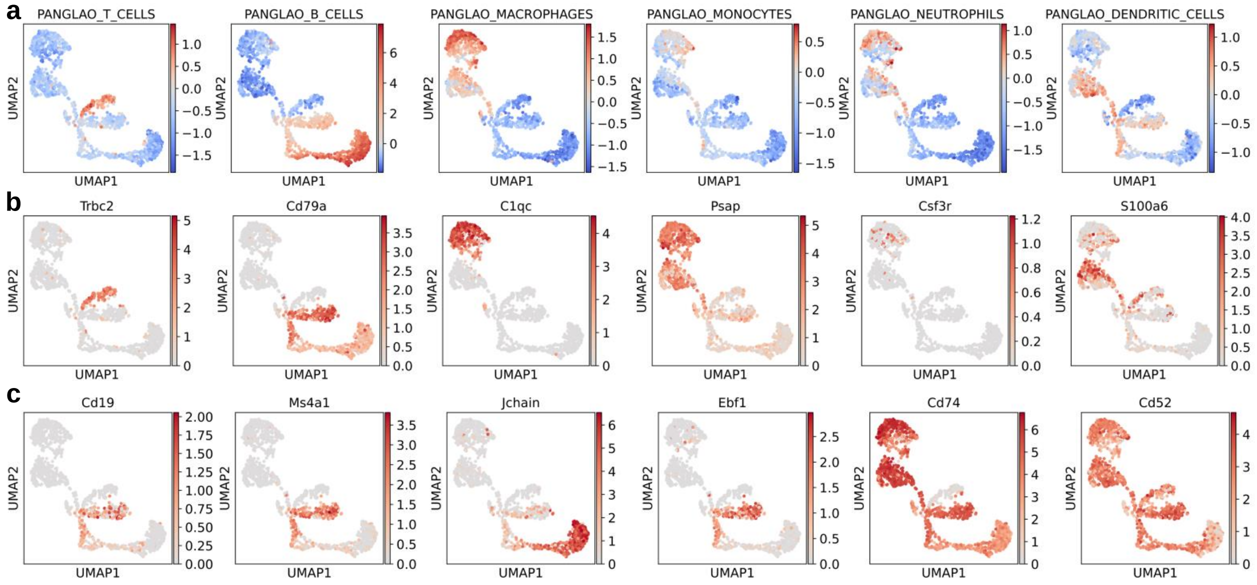

Supplementary Figure 8 | Comparison of immune cell type expiMap scores and manually curated cell type markers on an immune-only subset of the pancreatic query dataset. (a) PanglaoDB-based immune cell type scores from expiMap. **(b)** Expression of known immune cell type markers, corresponding to cell types as reported in **(a)** (aligned vertically). **(c)** Expression of additional known non-activated B cell (*Cd19*, *Ms4a1*), activated plasma B cell (*Jchain*), and top PanglaoDB B cell (*Ebf1*, *Cd74*, *Cd52*) markers.

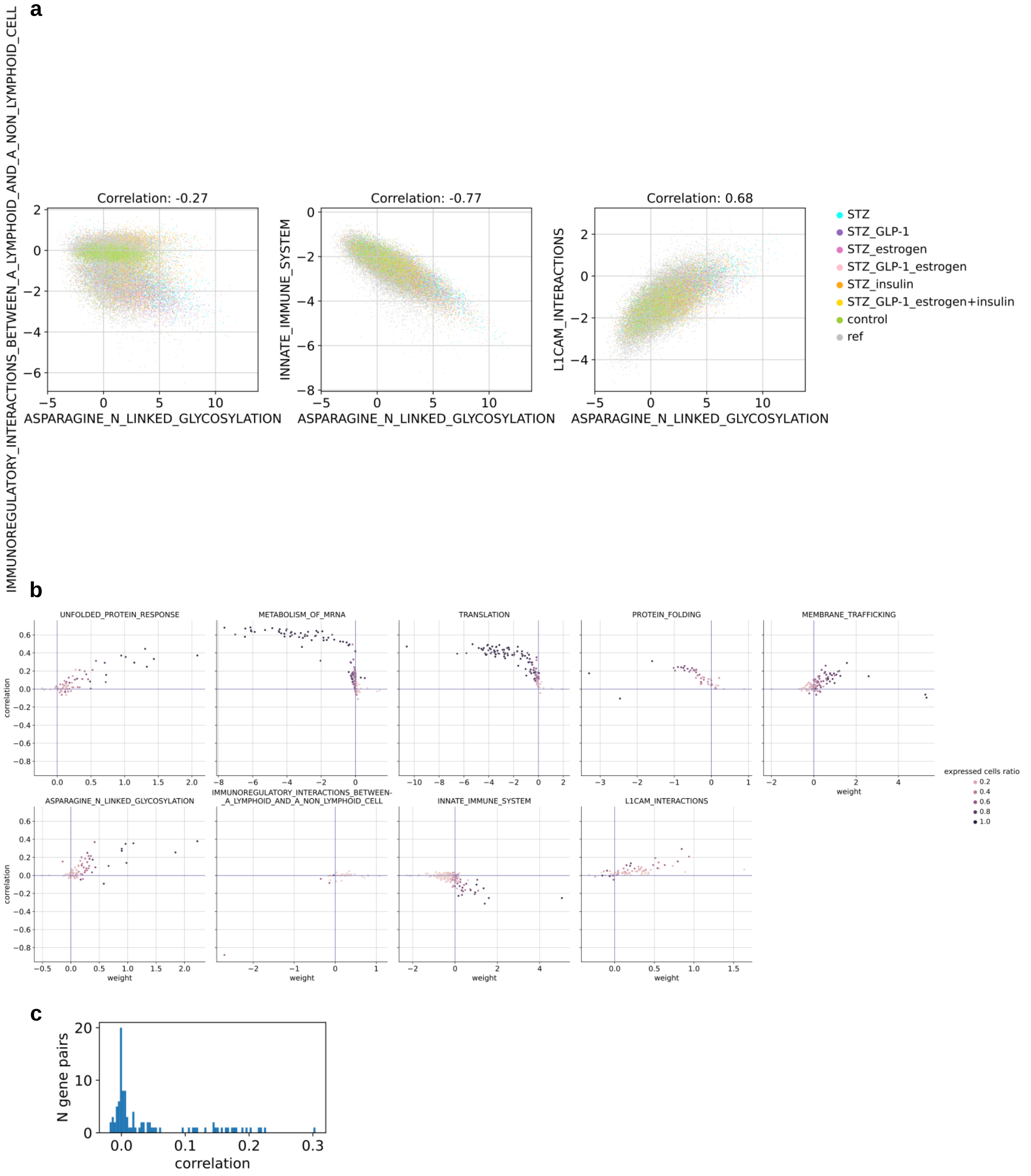

Supplementary Figure 9 | Extended analysis of GP correlations within pancreatic beta cells. **(a)** Comparison of beta cell scores of immune-related terms differentially active in T2D-model beta cells and asparagine N-linked glycosylation term. Ref: reference datasets, other samples are from the query dataset; STZ: streptozotocin T2D-model; STZ_GLP-1: streptozotocin T2D-model treated with GLP-1; STZ_estrogen: streptozotocin T2D-model treated with estrogen; STZ_GLP-1_estrogen: streptozotocin T2D-model treated with GLP-1-estrogen conjugate; STZ_insulin: streptozotocin T2D-model treated with insulin; STZ_GLP-1_estrogen+insulin: streptozotocin T2D-model treated with GLP-1-estrogen conjugate and insulin; control: healthy control. **(b)** Correlation of individual GP genes (dots) with their GPs within beta cells. Also shown is the relationship with GP gene weights and gene expression given as a ratio of cells expressing the gene. **(c)** Distribution of expression correlations between 10 genes with the highest absolute GP weights from the GPs asparagine N-linked glycosylation and innate immune system.

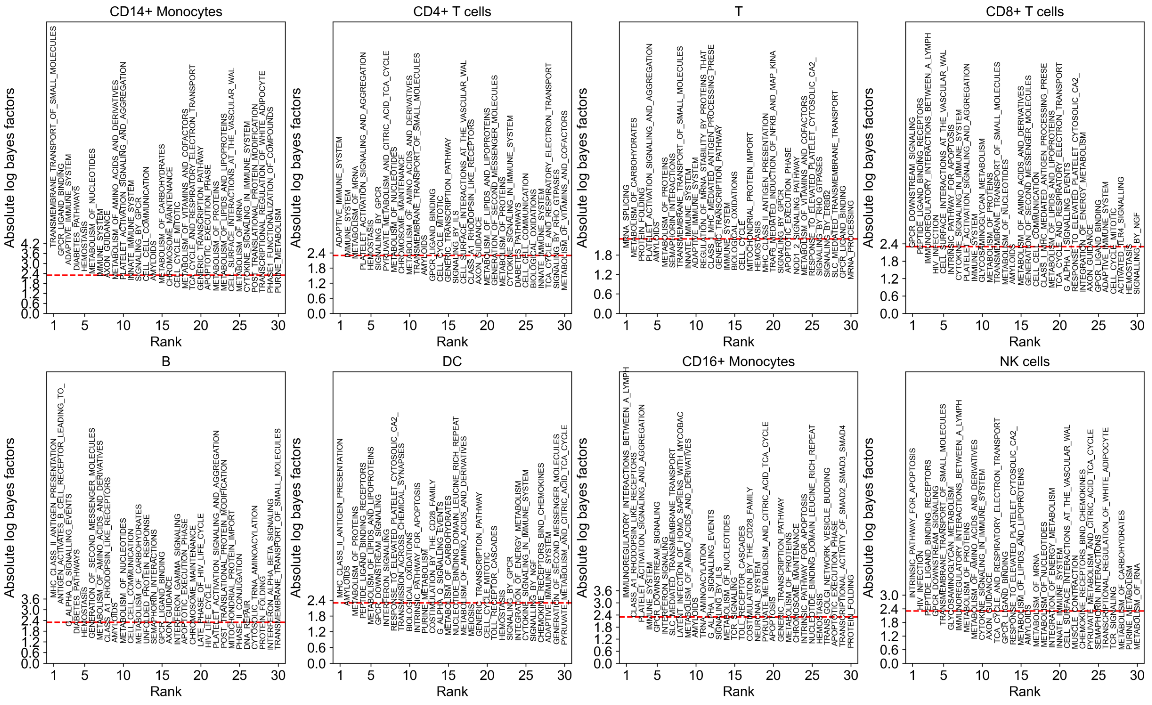

Supplementary Figure 10 | Differential GP analysis results for cell types from the IFN-β dataset only. (a) Differential GP analysis results between cell types (one vs all test) for cell types in the query data. The x-axis is the ranking of GPs; the y-axis denotes the significance (absolute log-Bayes factor) of each GP.
