# Supplementary material for: Biologically informed deep learning to query gene programs in single-cell atlases
Source: Nat Cell Biol. 2023 Feb 2;25(2):337–50. doi: 10.1038/s41556-022-01072-x (PMC9928587; doi:10.1038/s41556-022-01072-x)
Supplement: Supplementary file 2 — Reporting Summary [file 41556_2022_1072_MOESM2_ESM.pdf]

nature portfolio

Corresponding author(s): Fabian Theis

Last updated by author(s): Nov 21, 2022

Reporting Summary

Nature Portfolio wishes to improve the reproducibility of the work that we publish. This form provides structure for consistency and transparency in reporting. For further information on Nature Portfolio policies, see our Editorial Policies and the Editorial Policy Checklist.

Statistics

For all statistical analyses, confirm that the following items are present in the figure legend, table legend, main text, or Methods section.

n/a	Confirmed	
☐	☒	The exact sample size (*n*) for each experimental group/condition, given as a discrete number and unit of measurement
☒	☐	A statement on whether measurements were taken from distinct samples or whether the same sample was measured repeatedly
☐	☒	The statistical test(s) used AND whether they are one- or two-sided<br>*Only common tests should be described solely by name; describe more complex techniques in the Methods section.*
☐	☒	A description of all covariates tested
☐	☒	A description of any assumptions or corrections, such as tests of normality and adjustment for multiple comparisons
☐	☒	A full description of the statistical parameters including central tendency (e.g. means) or other basic estimates (e.g. regression coefficient) AND variation (e.g. standard deviation) or associated estimates of uncertainty (e.g. confidence intervals)
☐	☒	For null hypothesis testing, the test statistic (e.g. *F*, *t*, *r*) with confidence intervals, effect sizes, degrees of freedom and *P* value noted<br>*Give P values as exact values whenever suitable.*
☐	☒	For Bayesian analysis, information on the choice of priors and Markov chain Monte Carlo settings
☒	☐	For hierarchical and complex designs, identification of the appropriate level for tests and full reporting of outcomes
☐	☒	Estimates of effect sizes (e.g. Cohen's *d*, Pearson's *r*), indicating how they were calculated

Our web collection on statistics for biologists contains articles on many of the points above.

Software and code

Policy information about availability of computer code

Data collection	The software for expiMap is available from https://github.com/theislab/scarches<br>The development branch used for the paper https://github.com/theislab/scarches/tree/soft_new_mask
Data analysis	The data supporting the findings of this study can be reproduced using codes and notebooks available at https://github.com/theislab/expiMap_reproducibility.<br>The packages and software used for analysis:<br>Python 3.8, scanpy 1.8.1, scikit-learn 0.24.1, scipy 1.6.1, scvi-tools 0.14.2<br>R 4.1.0, Seurat 4.0.3, symphomy 0.1.0, CellChat 1.5.0, Limma 3.46.0, edgeR 3.32.1

For manuscripts utilizing custom algorithms or software that are central to the research but not yet described in published literature, software must be made available to editors and reviewers. We strongly encourage code deposition in a community repository (e.g. GitHub). See the Nature Portfolio guidelines for submitting code & software for further information.

Data

Policy information about availability of data

All manuscripts must include a data availability statement. This statement should provide the following information, where applicable:

- Accession codes, unique identifiers, or web links for publicly available datasets
- A description of any restrictions on data availability
- For clinical datasets or third party data, please ensure that the statement adheres to our policy

The Immune healthy atlas, PBMC IFN-β, PBMC COVID-19, Mouse endocrinogenesis datasets and the heart dataset used for the integration benchmark are public, referenced and downloadable at https://github.com/theislab/expiMap_reproducibility. The Pancreas datasets are publicly available and can be accessed with the following GEO codes: STZ (GSE128565), Fltp_P16 (GSE161966), NOD (GSE144471), spikein_drug (GSE147203/GSE142465 (GSM4228185 - GSM4228199)), NOD_elimination (GSE117770). The PBMCs, lung, colon liver datasets used in the integration benchmark are public, referenced and can be obtained from the sfaira database https://theislab.github.io/sfaira-portal/. The data supporting the findings of this study can be reproduced using codes and notebooks available at https://github.com/theislab/expiMap_reproducibility. All other data supporting the findings of this study are available from the corresponding author on reasonable request.

Human research participants

Policy information about studies involving human research participants and Sex and Gender in Research.

Reporting on sex and gender	Not applicable here
Population characteristics	Not applicable here
Recruitment	Not applicable here
Ethics oversight	Not applicable here

Note that full information on the approval of the study protocol must also be provided in the manuscript.

Field-specific reporting

Please select the one below that is the best fit for your research. If you are not sure, read the appropriate sections before making your selection.

☒ Life sciences ☐ Behavioural & social sciences ☐ Ecological, evolutionary & environmental sciences

For a reference copy of the document with all sections, see nature.com/documents/nr-reporting-summary-flat.pdf

Life sciences study design

All studies must disclose on these points even when the disclosure is negative.

Sample size	All datasets used in this paper are publicly available and not generated for this study. The sample size for each study is reported in each figure.
Data exclusions	For pancreas datasets we removed low-quality cells (high mitochondrial fraction, low number of genes) using a study-specific thresholds. For the other datasets we have not excluded any observations from original studies. We also removed genes not in gene sets annotations and selected higly variable genes according to the procedure in scanpy software. The detailed explanation of the preprocessing for each dataset is provided in Methods, section Datasets and preprocessing.
Replication	This is not relevant for out study since we did not perform any an wet-lab experiment for this paper. The replication of computational experiments can be found in https://github.com/theislab/expiMap_reproducibility.
Randomization	This is not relevant for out study since we did not perform any an wet-lab experiment for this paper.
Blinding	This is not relevant for out study since we did not perform any an wet-lab experiment for this paper.

Reporting for specific materials, systems and methods

We require information from authors about some types of materials, experimental systems and methods used in many studies. Here, indicate whether each material, system or method listed is relevant to your study. If you are not sure if a list item applies to your research, read the appropriate section before selecting a response.

Materials & experimental systems

n/a	Involved in the study
☒	☐ Antibodies
☒	☐ Eukaryotic cell lines
☒	☐ Palaeontology and archaeology
☒	☐ Animals and other organisms
☒	☐ Clinical data
☒	☐ Dual use research of concern

Methods

n/a	Involved in the study
☒	☐ ChIP-seq
☒	☐ Flow cytometry
☒	☐ MRI-based neuroimaging
